# An Evaluation of Healthy Hydration Recommendations for 93 Countries with Sugary Beverage Tax Legislation Globally, 2000–2023

**DOI:** 10.3390/nu16142264

**Published:** 2024-07-13

**Authors:** Nicole Leary, Molly K. Parker, Sofía Rincón Gallardo Patiño, Vivica I. Kraak

**Affiliations:** 1 Department of Human Nutrition, Foods, and Exercise, Virginia Polytechnic Institute and State University (Virginia Tech), Blacksburg, VA 24061, USA; pmolly95@vt.edu (M.K.P.); vivica51@vt.edu (V.I.K.); 2 Cardiovascular Health Program and Food & Nutrition Portfolio, Global Health Advocacy Incubator, Washington, DC 20005, USA; srincon@advocacyincubator.org

**Keywords:** sugary beverage taxes, sugary beverages, water, food-based dietary guidelines, healthy beverage guidelines, healthy hydration, policy coherence

## Abstract

Adequate water intake is essential for human health. Sugary beverage taxes are a best buy policy to reduce obesity and diet-related non-communicable diseases. Food-based dietary guidelines (FBDGs) promote healthy dietary patterns. The study purpose was to evaluate national FBDGs for countries with sugary beverage tax legislation (2000–2023) to promote water and discourage sugary beverage consumption. We developed a coding framework to conduct a content analysis of FBDG documents, and used six indicators to identify messages and images to assign healthy hydration recommendation (HHR) scores from 0–12 to compare FBDGs across countries and six regions. Results showed 93 countries with sugary beverage tax legislation of which 58 countries (62%) had published FBDGs (1998–2023). Of 58 FBDGs reviewed, 48 (83%) had complementary recommendations that encouraged water and discouraged sugary beverages. Of 93 countries, 13 (14%) had the highest HHR scores (11–12); 22, (24%) had high HHR scores (9–10); 20 (21%) had medium HHR scores (4–8); 3 (3%) had low HHR scores (0–3); and 35 (38%) countries had no FBDGs. To reduce health risks for populations, governments must ensure policy coherence to optimize sugary beverage tax impacts by developing FBDGs that encourage water and discourage sugary beverages complementary to national policies.

## 1. Introduction

Adequate and safe water consumption is essential for healthy hydration to optimize human health [1]. Sugary beverages are broadly defined as beverages low in nutritional quality that contain free or added sugars, such as carbonated and non-carbonated soft drinks (i.e., soda), fruit drinks, sports drinks, and energy drinks [2,3]. These types of beverages are major sources of excessive added sugars in diets [2]. The global ready-to-drink, non-alcoholic sugary beverage market was worth nearly $750 billion United States dollars (USD) in 2023 and is projected to exceed USD $1.2 trillion by 2032 [3,4]. Regular consumption of sugary beverage products is associated with low diet quality and contributes to unhealthy weight gain, obesity, and other health risks including: insulin resistance and type 2 diabetes, cardiovascular disease, dental caries and cancer among children, adolescents, and adults [2,5,6,7,8,9,10,11].

### 1.1. Healthy Beverage Guidelines to Reduce Sugary Beverage Health Risks

In 2019, the World Health Organization (WHO) and Food and Agricultural Organization (FAO) of the United Nations recommended that governments designate and promote water as the healthy default beverage to support sustainable healthy diets [12]. The WHO also recommended that individuals consume less than 10% of total energy from free or added sugars daily, which is less than 25–50 g/day or ~6 teaspoons of added sugars for children and 10–12 teaspoons of added sugars for adults based on average daily energy intake [13]. The WHO also noted additional benefits when free or added sugars are less than 5% of total energy daily [13].

The FAO defines food-based dietary guidelines (FBDGs) as “context-specific advice and principles on healthy diets and lifestyles, which are rooted on sound evidence, and respond to a country’s public health and nutrition priorities, food production and consumption patterns, sociocultural influences, food composition data, and accessibility, among other factors” [14]. National FBDGs are used to inform food, nutrition, and agricultural policies and programs to improve diet quality, food systems, and health [14].

Two published reviews of national FBDG documents examined healthy beverage recommendations and messages that encouraged water as the default beverage and discouraged sugary beverages [15,16]. Herforth et al. 2019 [15] examined national FBDGs for countries across six regions (1986–2017) and reported that 56.4% recommended water through food guides and 46% recommended that populations limit or avoid sugary beverage intake through key messages. James-Martin et al. 2022 [16] used an environmental sustainability lens to review how the national FBDGs promoted the 16 FAO and WHO guidelines for sustainable healthy diets [12]. These investigators reported that only 27% (*n* = 10) of national FBDGs mentioned drinking water, 11% (*n* = 4) mentioned why water was important and how to access it, and 3% (*n* = 1) provided information on the quantity of water to drink daily [16].

### 1.2. Policies and Legislation to Reduce Sugary Beverage Health Risks

Expert bodies recommend sugary beverage taxes as a cost-effective best buy policy to prevent non-communicable disease (NCD) risks for populations and encouraged member states to enact legislation to support a national sugary beverage tax [17,18,19]. In 2022, the WHO published guidelines for government policy development, implementation, monitoring, and evaluation of sugary beverage taxes [20]. By 2024, nearly 117 countries and sub-national jurisdictions or territories, representing about half (57%) of the world’s population, had introduced or enacted legislation to discourage sugary beverages using a revenue generating excise tax or sugar reformulation levy [19,21,22].

Sugary beverage taxes vary depending on their purpose and design, such as: (1) excise taxes (i.e., levied on the manufacturer or at import on health-damaging products), (2) import taxes (i.e., collected at the port of entry for domestic consumption), (3) value added tax (i.e., levied on a percentage of the product’s value), or (4) sales tax (i.e., levied on a percentage of the retail value) [21]. Sugary beverage tax design include: (1) ad valorem (i.e., percentage of the value of the product), (2) specific (i.e., fixed amount based on a measurement) and/or (3) a mix of both ad valorem and specific tax structures. Tax rate structures may be uniform (i.e., single rate on all products) or tiered (i.e., multiple rates on all products). The beverage products taxed can vary, and taxes can be targeted (i.e., apply to sugary beverages at a higher rate than unsweetened bottled water) versus untargeted (i.e., apply to unsweetened bottled water at the same or a higher rate than sugary beverages) [21].

Evaluations of sugary beverage excise tax or levy legislation across regions, countries, and jurisdictions have shown that most have increased the price of targeted beverages (i.e., soda and carbonated drinks) and reduced sales by 15 percent [21,23,24,25]. Additionally, sugary beverage taxes have been determined to increase water purchases among some countries and jurisdictions [26,27]. However, the impact of sugary beverage taxes and levies on the cognitive, diet, and health outcomes of diverse populations vary across high-, middle- and low-income country contexts [2,21,23,24,25,28]. No single policy has effectively encouraged individuals and populations to select water as the healthy default to support healthy hydration and discouraged sugary beverage availability, access, and intake over the long-term [29,30].

### 1.3. Policy Coherence for Healthy Hydration to Reduce Sugary Beverage Health Risks

Policymakers must aim to achieve policy coherence and align policy efforts to ensure various public health, nutrition, and food policies strengthen each other. [31]. Policy coherence aims to maximize opportunities; expose trade-offs; and minimize inconsistencies, inefficiencies, and contradictions that may reduce or undermine the collective impact of policies to achieve goals and objectives [31]. The FAO’s food systems-based dietary guidelines encourage member states to promote the coherence of policies, strategies, and programs to encourage sustainable healthy diets through national, sub-national, and local food systems [32].

Policy coherence is important for governments to transition to more resilient, healthy, equitable, and sustainable diets and agri-food systems at different scales [33]. Recent studies have examined policy coherence to encourage prudent water management practices in response to climate change [33], taxation of sugar as a commodity to meet climate and sustainability goals [34], strengthen food security and disaster risk reduction for countries [35], and for governments to implement supportive international trade and nutrition policies [36]. Policy coherence is also important to strengthen the climate change mitigation potential of national FBDGs to address health and ecological issues across the food life cycle, food groups, and dietary patterns [37]. Water security impacts human health by ensuring healthy hydration, hygiene, and sanitation; protecting resilience and biodiversity; supporting livelihoods and businesses for economic prosperity; and ensuring that people and animals have regular access to clean water [38]. Limited research has examined how policy coherence may support a portfolio of synergistic strategies to promote water and discourage sugary beverage availability, affordability, marketing, and consumption among diverse target populations across various settings and sectors [39,40].

### 1.4. Study Purpose

While there has been substantial progress adopting and implementing sugary beverage taxes globally, policymakers must implement complementary policies to enable access to free and safe drinking water for healthy hydration. No study has documented the healthy hydration recommendations for countries or jurisdictions that have enacted national or sub-national sugary beverage tax or levy legislation aligned with other policies. The study purpose was to evaluate national FBDGs for healthy hydration in countries with sugary beverage tax legislation (2000–2023) across the WHO’s six regions. We examined the content of the text and visual images used in technical and graphic FBDG documents that encourage water as the healthy default and discourage sugary beverage consumption to reduce the associated health risks for populations. The findings are discussed within the context of policy, systems, and environmental (PSE) change strategies. We also examine how governments and other food system stakeholders could strengthen policy coherence for healthy hydration by developing and implementing comprehensive national dietary guidelines linked to other policies, including legislation and laws, to optimize water availability, access, promotion and use and reduce sugary beverage health risks for populations. 

## 2. Materials and Methods

This study used a mixed-methods research design to ensure credibility, verifiability, and validity. First, we developed four research questions described below. Second, we designed a search strategy that involved a stepped review of several evidence sources to support data triangulation. Third, we developed a coding framework to conduct a qualitative content analysis of the FBDG documents selected. Fourth, the co-investigators independently reviewed the evidence sources to maximize investigator triangulation. Finally, we determined a healthy hydration recommendation (HHR) score (i.e., high, medium, and low) for the countries with sugary beverage tax legislation.

### 2.1. Research Questions

This study was guided by four research questions (RQs):

RQ1: How many and which countries across the WHO’s six regions enacted and/or updated their sugary beverage tax or levy legislation between 2000 and 2023?
RQ2: What are the most current published versions of the national technical and graphic FBDG documents for countries with sugary beverage tax legislation?
RQ3: Which countries have issued healthy hydration recommendations to promote water and reduce, replace, or avoid sugary beverage intake and how do the text and graphic messages of the healthy beverage recommendations for countries compare and contrast in the national FBDGs?
RQ4: What is the healthy hydration recommendation (HHR) score for countries with FBDG documents and sugary beverage tax legislation and how do the scores compare across the WHO’s six regions?

### 2.2. Search Strategy, Evidence Selection and Extraction

To address RQ1, we identified countries that enacted or updated a targeted sugary beverage tax or levy between 2000 and 2023 and classified them within the WHO’s six regional offices (i.e., Africa, Eastern Mediterranean, Europe, Americas, Southeast Asia, and Western Pacific) to map the regional healthy hydration scores and inform policy coherence [41]. Countries with targeted sugary beverage taxes were included in this study due to the nature of the tax promoting water consumption and discouraging sugary beverages by applying to sugary beverages at a higher rate than unsweetened bottled water [21]. Countries were excluded from the study if they had untargeted taxes applied at the same rate for sugary beverages and water or had repealed sugary beverage tax legislation within the search period. The findings were triangulated across several evidence sources including: Hattersley and Mandeville 2023 [21]; The World Bank’s Global Sugary Beverage Tax Database [22]; and University of North Carolina at Chapel Hill’s Global Food Research Program’s Sweetened Soft Drinks Tax Map [42].

To address RQ2 and RQ3, we searched the FAO website that provides government-endorsed, national technical and graphic FBDGs by country and region [43]. For countries that were not included on the FAO’s online database, a supplemental search was conducted in PubMed and Google Scholar through 31 December 2023 using the following search terms: (“food-based dietary guidelines” OR “FBDG”) AND (“country name”).

We created an evidence table to list the current healthy beverage recommendations for consuming water and reducing or avoiding sugary beverages to support a healthy weight and reduce obesity and diet-related NCD risks. This study focused on the promotion of water. While unflavored and unsweetened milk is recognized as a healthy beverage and included in several national FBDGs [15,44], this study excluded fluid milk from cows or other animals, plant-source dairy and non-dairy milk products, or beverages with non-sugar artificial or high-intensity sweeteners. The rationale for this exclusion is that many countries include fluid milk products in the protein category of FBDGs. There is currently a lack of scientific consensus and guidance for whether beverages that contain non-sugar additives (i.e., artificial, natural and sugar alcohols) as a substitute for added sugars in low- or no-calorie products in the marketplace are considered healthy. A 2023 WHO report did not recommend non-sugar sweeteners for individuals to manage weight due to the potential adverse effects of long-term use [45].

We examined the methodology of several published papers that had analyzed the textual messages and/or graphic content of national FBDG documents [16,46,47,48,49,50,51]. As described by Kois et al. 2022 [50], FBDGs are available in three formats (i.e., detailed technical guidelines, short guides, and the FAO translated summaries). We first examined the detailed technical guidelines when available, followed by the short user-friendly guides aimed at individuals or the lay public. If the national FBDG documents were not available, we used the FAO’s translated key messages from the agency’s website or used secondary sources that described the content of the FBDG document using published studies and grey literature reports. When no FBDG documents were found, we confirmed this finding by searching the nutrition profiles for these countries using the online Global Nutrition Report 2022 database [52].

To address RQ3, the FBDG documents were downloaded and categorized by the WHO’s six regions (i.e., Africa, Eastern Mediterranean, Europe, Americas, Southeast Asia, and Western Pacific). The rational for categorizing the countries using the WHO regions rather than the FAO regions is because the WHO is the lead UN agency that provides technical assistance and best practices for diet and health-related policies and programs to member states across six global regions. Using the WHO six regions provides a structure that is recognized globally which can support effective communication, policymaking, and help address the impact of policies in different settings.

Thereafter, we conducted a keyword search to identify healthy beverage recommendations using the search function for “water”, “hydration”, “sugar-sweetened beverage”, “sugary beverages”, “beverages”, and “drink”. For the FBDGs published in Spanish, S.R.G.P. conducted the keyword search in the Spanish language using the search function for “agua”, “hidratación”, “bebida”, “azucarada”, “bebida endulzada”, and “bebida”. The terms “refresco” and “soda” were also searched among the FBDGs published in Spanish. For the FBDGs not published in English or Spanish, one investigator (N.L.) used Google translate to identify the healthy beverage recommendations by translating the terms “water”, “hydration”, “sugar-sweetened beverage”, “sugary beverages”, “beverages” and “drink”. The FAO website and translated key messages were referenced for countries with technical FBDG documents that were not published in English and did not allow for a keyword search. 

**Figure 1:**
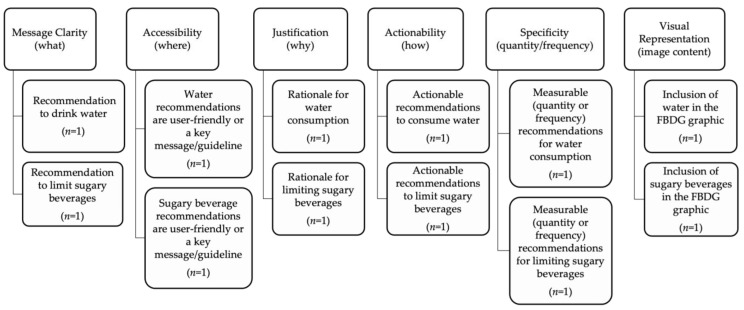
Coding framework to assess the FBDG healthy beverage recommendations by country and WHO region.

After an initial review of the technical and graphic FBDG documents, the co-investigators developed a coding framework, adapted from previously published studies [16,46,47,48,49,50,51], to conduct a comprehensive qualitative content analysis of FBDG documents. Figure 1 shows the six criteria used in the coding framework including: message clarity (what), accessibility for consumers to view messages (where), justification (why), actionability (how), specificity (quantity/frequency), and visual representation (image content). Supplementary Table S1 provides more details on the coding process. Three co-investigators (N.L., M.K.P., and S.R.G.P.) extracted the healthy beverage recommendations verbatim, and categorized them according to the coding framework and master codebook to encourage water and discourage sugary beverage consumption (Supplementary Tables S4–S9). Given the length and breadth of the FBDG technical documents, the investigators used thematic coding and their professional judgement to categorize the FBDG content based on the predetermined coding framework indicators and themes. Recommendations that did not pertain directly to healthy hydration were not included in the evidence table. The recommendations were selected examples from FBDGs and were not comprehensive of every relevant recommendation. The co-investigators independently reviewed, coded, and categorized the FBDGs and met to resolve any different interpretations of the evidence. The senior co-investigator (V.I.K.) provided input to reconcile differences for the interpretation of evidence before synthesizing the results.

To address RQ3 and RQ4, we used a qualitative content analysis to compare keywords, followed by the interpretation of the textual and graphic image content from the selected FBDG documents across countries and regions using the coding framework (Figure 1) [53]. The national FBDG beverage recommendations were scored based on six indicators used to examine the FBDG documents (Supplementary Table S1). Each FBDG had a potential HHR score of two points (*n* = 2) for message clarity (what), accessibility of key messages (where), justification (why), actionability (how), specificity (quantity/frequency), and visual representation (image content). The top possible HHR score was 12 if a country’s FBDG comprehensively promoted healthy hydration by encouraging water and discouraging sugary beverage intake. Across the coding framework (i.e., what, where, why, how, quantity/frequency, and visual representation), one point was designated for promoting healthy hydration and one point for discouraging sugary beverages.

For message clarity (what), one point was designated if the message clearly promoted water and one point was designated if the message clearly discouraged sugary beverages. For the accessibility (where), one point was designated if the textual content promoting water was bolded and included in a text box, or clearly highlighted as a key message or guideline for consumers to easily access, and one point was designated if the textual content discouraging sugary beverages was bolded and included in a text box, or clearly highlighted as a key message or guideline. The co-investigators used their professional judgment when assessing whether the messages were user-friendly and accessible to the public.

For the justification (why), one point was designated if a rationale was provided as to why the population should increase water consumption and one point was designated if the rationale to decrease sugary beverage intake was included. For the actionability (how), one point was designated if actionable messages were provided promoting water consumption and one point was designated if actionable messages described decreasing sugary beverage consumption. For specificity (quantity or frequency), one point was designated if there was a measurable recommendation for water and one point was designated if there was a measurable recommendation for sugary beverages. The co-investigators also reviewed the FBDG documents for measurable broader total dietary intake of added sugar recommendations, and these were included during the coding and message extraction process because they could be applied specifically to sugary beverages. For the visual content, one point was designated if the graphic FBDG showed and encouraged water, and one point was designated if the image showed and discouraged sugary beverages.

The country-level HHR score was calculated by adding the total points determined by the coding framework based on the codebook. Countries with 0–3 were assigned a low HHR score; 4–8 were assigned a medium HHR score; and 9–12 were assigned a high HHR score. The regional HHR score was determined by calculating the average score among the countries within each of the six WHO regions. The results are synthesized as a narrative summary.

## 3. Results

### 3.1. Countries with Sugary Beverage Taxes or Levies across the Six WHO Regions

The results showed that 93 countries had enacted or updated targeted, national, or sub-national sugary beverage tax or levy legislation between 2000 and 2023. Countries were excluded from the evaluation if they had repealed their sugary beverage tax or were not listed in the databases review; and 28 countries were excluded due to untargeted sugary beverage taxes (i.e., Argentina, Belize, Brazil, Burundi, Cameroon, Chad, Costa Rica, Egypt, The Gambia, Guinea-Bissau, Kenya, Lao People’s Democratic Republic, Liberia, Madagascar, Mauritania, Micronesia, Netherlands, Nicaragua, Palau, Paraguay, Rwanda, Senegal, South Sudan, Suriname, Uganda, United Republic of Tanzania, Uruguay, and Zimbabwe) [22]. Countries were also excluded if there was no evidence of a tax through a supplemental Google search by “country name” and “sugary beverage tax” implemented or updated prior to 2000.

### 3.2. National Technical and Graphic FBDG Documents Available across the WHO Regions

Ninety-three countries had enacted or updated national or sub-national targeted sugary beverage taxes or levy legislation between 2000 and 2023. Of these countries and jurisdictions, 62% (*n* = 58) had national technical FBDGs and 56% (*n* = 52) had graphic FBDGs published between 1998 and 2023. Table 1 outlines the countries across the six WHO regions, the implementation dates of the most current sugary beverage tax or levy legislation between 2000 and 2023, and the technical FBDG identified through this review. Supplementary Table S2 provides additional details regarding the sugary beverage tax for each country.

The WHO African Region is comprised of 47 member states or countries [54]. Among the 21 countries in the WHO African Region that enacted or updated sugary beverage tax or levy legislation since 2000, eight countries (38%) have published FBDGs, including: Benin, Ethiopia, Gabon, Ghana, Nigeria, Seychelles, South Africa, and Zambia [55,56,57,58,59,60,61,62]. The WHO Eastern Mediterranean Region is comprised of nearly 745 million people who live in 21 member states or countries [63]. Among the eight countries in the WHO Eastern Mediterranean Region that have enacted or updated sugary beverage tax or levy legislation since 2000, six countries issued FBDGs (75%), including Bahrain, Oman, Pakistan, Qatar, Saudi Arabia, and United Arab Emirates (UAE) [64,65,66,67,68,69]. Additionally, the 2012 Arab Food Dome offers dietary guidelines for the people living in the Arab-speaking Gulf region to prevent diet-related disease risks [70,71].

The WHO Europe Region is comprised of 53 countries [72]. Among the 20 countries in the WHO Europe Region that enacted or updated sugary beverages taxes or levy legislation since 2000, 13 countries (65%) have FBDGs including: Belgium, Croatia, Finland, France, Hungary, Ireland, Latvia, Poland, Portugal, Romania, Spain, Türkiye, and the United Kingdom (UK) [73,74,75,76,77,78,79,80,81,82,83,84,85,86,87,88,89,90]. The Pan American Health Organization (PAHO)/WHO Americas Region is comprised of 35 countries [91]. Among the 18 countries within the PAHO/WHO Americas Region that enacted or updated a sugary beverage tax or levy legislation since 2000, all countries (100%) have FBDGs including Barbados, Bermuda, Bolivia, Canada, Chile, Colombia, Dominica, Ecuador, El Salvador, Grenada, Guatemala, Honduras, Mexico, Panama, Peru, Saint Kitts and Nevis, Saint Vincent and the Grenadines, and the United States (US) [92,93,94,95,96,97,98,99,100,101,102,103,104,105,106,107,108,109].

The WHO Southeast Asia Region is comprised of 11 countries and is home to over a quarter of the world’s population [110]. Among the seven countries within the WHO Southeast Asia Region that enacted or updated a sugary beverage tax or levy legislation since 2000, six countries (86%) have FBDGs including Bangladesh, India, Maldives, Nepal, Sri Lanka, and Thailand [111,112,113,114,115,116,117]. The Western Pacific region is home to approximately 1.9 billion people across 37 countries and territories [118]. Among the 19 countries within the WHO Western Pacific Region that have enacted or updated a sugary beverage tax or levy legislation since 2000, seven countries (37%) have FBDGs including Brunei, Cambodia, Fiji, Malaysia, Marshall Islands, the Philippines, and Tuvalu [119,120,121,122,123,124,125]. Additionally, the Pacific Guidelines for Healthy Living, released in 2018, provided guidelines for healthy living to all Pacific countries and included a graphic image [126]. While the FBDGs for many Western Pacific countries were not identified through this study, the FAO published a technical support document in 2022 that highlighted actions taken to develop and update FBDGs in Fiji, Marshall Islands, Samoa, Solomon Islands, Tonga, and Vanuatu [127]. This document concluded that the FBDGs for these countries are partially completed and based on the Pacific Guidelines for Healthy Living. An additional 2018 report by The Pacific Monitoring Alliance for NCD Action, highlighted that several countries (i.e., Cook Islands, French Polynesia, Kiribati, New Caledonia, Niue, Samoa, Tonga, and Vanuatu) have FBDGs [128] that were not identified by our evidence search process. 

### 3.3. Healthy Beverage Recommendations in FBDGs for Countries across the WHO Regions

Among the 58 FBDGs analyzed, 56 (97%) included healthy beverage recommendations to encourage water consumption, discourage sugary beverages, or a combination of both messages. A total of 48 of the reviewed FBDGs (83%) included healthy beverage guidelines that encouraged water and discouraged sugary beverages through a combination of complementary recommendations and messages. The clarity, accessibility, justification, actionability, specificity, and visual representation of the messages varied across countries. A total of 54 countries included clear messages encouraging water consumption and 50 countries included clear messages discouraging sugary beverage consumption. Supplementary Table S3 shows the graphic FBDGs for countries across the six WHO regions. Supplementary Tables S4–S9 describe the extracted messages, HHR score for each country, and content from the analyzed technical and graphic FBDGs for countries across the WHO regions.

Among the African countries with sugary beverage tax legislation that have FBDGs, all promoted healthy hydration with water recommendations including: Benin, Ethiopia, Gabon, Ghana, Nigeria, Seychelles, South Africa, and Zambia [55,56,57,58,59,60,61,62]. Among these countries, Gabon lacked messages that discouraged sugary beverages [57]. Of the published FBDGs for the WHO Eastern Mediterranean Region, six countries promoted water consumption including Bahrain, Oman, Pakistan, Qatar, Saudi Arabia and UAE [64,65,66,67,68,69]. Saudi Arabia, Qatar, and Pakistan included recommendations that discouraged sugary beverages. Of the FBDGs for the WHO Europe Region, 11 countries promoted healthy hydration through recommendations to consume water and decrease sugary beverage consumption including Belgium, Finland, France, Hungary, Ireland, Latvia, Poland, Portugal, Spain, Türkiye, and the UK [73,75,76,77,78,79,80,81,82,83,84,85,86,88,89,90]. Croatia’s FBDGs were published in 2002 and did not include recommendations to support healthy hydration through water consumption and reducing sugary beverages [74]. While the FBDGs for Romania had recommendations to consume water, there were no messages that discouraged sugary beverages [87].

Among the countries with FBDGs for the PAHO/WHO Americas Region, 18 countries (i.e., Barbados, Bermuda, Bolivia, Canada, Chile, Colombia, Dominica, Ecuador, El Salvador, Grenada, Guatemala, Honduras, Mexico, Panama, Peru, Saint Kitts and Nevis, Saint Vincent and the Grenadines, and the United States) promoted healthy hydration through either specific recommendations, key messages, or images to consume water and/or reduce consumption of sugary beverages [92,93,94,95,96,97,98,99,100,101,102,103,104,105,106,107,108,109]. Of the FBDGs identified among the WHO Southeast Asia Region, five countries, Bangladesh, India, Maldives, Nepal, and Sri Lanka, promoted healthy hydration with recommendations to consume water and included messages discouraging sugary beverage consumption [111,112,113,114,115]. Among the FBDGs examined for the WHO Western Pacific Region, Brunei, Fiji, Malaysia, Marshall Islands, the Philippines, and Tuvalu promoted healthy hydration with recommendations to consume water and reduce sugary beverage consumption [120,121,122,123,124,125], while Cambodia’s FBDGs only discouraged sugary beverages [119].

#### Healthy Hydration Recommendation Scores by Country and Region

Among the 93 countries that had enacted sugary beverage taxes or levy legislation to discourage consumption of sugary beverages, *n* = 13 (14%) had the highest HHR scores (11–12); *n* = 22 (24%) had high HHR scores (9–10); *n* = 20 (21%) had medium HHR scores (4–8); *n* = 3 (3%) had low HHR scores (0–3); and *n* = 35 (38%) had no FBDGs. Of the countries with low HHR scores, one country (i.e., Gabon) scored a 3 due to weak recommendations; two countries (i.e., Croatia and Thailand) scored a 0–1 due to not including explicit healthy beverage guidelines within the national FBDGs; and 35 countries had not published technical or graphic FBDG documents. 

**Figure 2:**
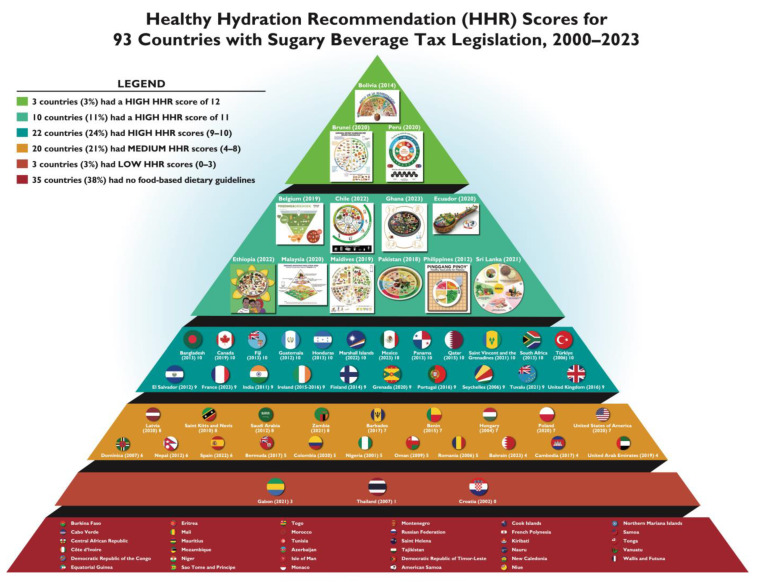
Healthy hydration recommendation (HHR) scores for 93 countries with sugary beverage tax legislation, 2000–2023.

The countries that had enacted sugary beverage tax or levy legislation across the WHO six regions had hydration recommendations that varied in relation to the clarity, consumer accessibility, justification, actionability, specificity, and visual representation of the messages encouraging water and discouraging sugary beverages. When calculating the average score by region, the WHO/PAHO Americas Region scored the highest HHR score demonstrated by a 9 out of 12 possible points, followed by the Southeast Asia Region (6.9 out of 12), Eastern Mediterranean Region (5.3 out of 12), European Region (5 out of 12), Western Pacific Region (3.5 out of 12), and Africa Region (3.1 out of 12). Table 2 shows the HHR scores for countries with sugary beverage tax legislation across the six WHO regions. Table 3 summarizes the highest HHR scores (9–12) for 35 countries that enacted sugary beverage tax or levy legislation across six WHO regions. Figure 2 displays the HHR scores for 93 countries with sugary beverage tax legislation, 2000–2023.

## 4. Discussion

To our knowledge, this is the first published study that conducted an in-depth content analysis of HHR for countries with national FBDGs using a coding framework as an assessment tool. We used this evidence to evaluate the HHR scores for countries across the six WHO regions that enacted or updated their sugary beverage taxes or legislation (2000–2023). While 93 countries had enacted national or sub-national legislation to reduce sugary beverage consumption through targeted taxes and legislation, only 58 countries had national technical FBDGs, and 52 had published graphic FBDG documents (1998–2023) identified through our evidence search process. This demonstrates a lack of policy coherence among some countries with sugary beverage tax legislation. Among the reviewed FBDGs, 83% had complementary HHR that encouraged water and discouraged sugary beverages.

The impact of sugary beverage taxes could be greater when enacted with complementary policies to encourage healthy hydration and decrease consumption of sugary beverages [18]. This study used a novel approach to document the policy coherence among two policy tools, sugary beverages taxes or levy legislation, and national FBDGs hydration recommendations. This study provides a first step to evaluate the policy coherence for healthy hydration across the WHO regions. Additional policy tools must be evaluated and documented to fully assess the policy coherence among countries to promote healthy hydration.

### 4.1. FBDGs to Promote Healthy Hydration and Reduce Sugary Beverage Health Risks

Of the FBDG documents reviewed for this study, the level of clarity, accessibility, justification, actionability, specificity, and visual representation for HHR scores varied across countries and regions. While water and/or sugary beverages were addressed across the majority of the reviewed FBDGs, only three healthy beverage recommendations (i.e., Bolivia, Brunei, and Peru) included fully comprehensive guidelines holistically emphasizing what, where, why, how, quantity/frequency, and visual representation for both the encouragement of water and discouragement of sugary beverages. These results align with evidence concluding FBDGs must provide specific information (i.e., what, where, why, how, quantity/frequency, and visual representation) to promote aspects of healthy and sustainable diets, including promoting water as the healthy default beverage [16,46,47,48,50].

Among countries with high HHR scores (i.e., nine through twelve), healthy hydration guidelines commonly lacked a visual representation discouraging sugary beverages through the graphic FBDG. However, across all 93 countries that enacted sugary beverage or tax legislation, water was frequently included in graphic FBDGs. Among the FBDGs reviewed, 38 (73%) of the 52 graphic FBDGs identified through our evidence search process included a visual representation of water encouraging healthy hydration practices. A 2017 review of 79 graphic FBDG visual representations align these results [46]. While sugary beverages were not assessed through van’t Evre et al. 2017 [46] review, the investigators found 48% of graphic FBDG included non-caloric beverages (i.e., water, coffee, or tea). While the effectiveness of images on plate models and graphic FBDGs is unclear, this research contributes to the growing literature on the use of graphic FBDGs and plate models to promote healthy dietary patterns, such as increasing water consumption [47].

Specificity of a maximum amount of added sugar and/or sugary beverages were excluded from some technical FBDGs, including those that had high HHR scores. Among the reviewed FBDGs, several countries included specific amounts (quantity and frequency) of added sugar and/or sugary beverages. The recommendations were often in combination with other free or added sugar foods to represent an upper limit for daily consumption. Added sugar recommendations from Bangladesh, Bolivia, Canada, Ethiopia, Finland, Latvia, Malaysia, Maldives, Marshall Islands, Oman, Pakistan, Peru, South Africa, Sri Lanka, Thailand, Tuvalu, the UK, the US, and Zambia were aligned with the WHO recommendations on sugar intake for adults and children to consume less than 10% of total energy from free or added sugars daily [56,61,62,65,66,75,82,90,94,95,106,109,111,113,115,116,122,123,125]. These findings align with Koios et al. 2022 [50] content analysis of national FBDGs, which demonstrated that FBDGs often recommend to “eat less” sugar, however, the specificity of the message and inclusion of quantities and frequencies of consumption was not assessed. The results of this study expands on current research evaluating FBDGs by assessing the measurable recommendations (i.e., specificity) of the messages.

Few healthy hydration guidelines provided recommendations on the specific quantity of sugary beverages to limit or exclude from a healthy dietary pattern. However, South Africa’s FBDG stated that “adults and children should limit the consumption of SSBs to one tin per day, or the equivalent amount of added sugar from other foods” [61]. The Dietary Guidelines for Healthy Eating Brunei Darussalam states to “avoid or limit drinks with high sugar (i.e., containing more than 6 g of sugar per 100 mL)” [120]. The Dietary Guidelines for Belgian Adult Population encouraged consumers to “avoid sugary drinks with an energy content over 50 kcal per 227 mL serving or 22 kcal/100 mL, or that contain more than 5% sugar” [73]. The UK’s Eatwell Guide emphasized that fruit juices and smoothies should be considered as a source of added sugars and individuals should limit their consumption to no more than 150 mL per day [90]. These countries’ FBDGs demonstrate the specific messages that are further supported by legislation and policy discouraging the purchase and consumption of sugary beverages.

Some national FBDGs included healthy beverage recommendations for only a portion of the population. For instance, while the FBDGs for Nigeria—A Guide to Healthy Eating recommended that people avoid sugary beverages such as soft drinks, cocoa-based beverages and chocolate drinks, these guidelines were specific to individuals with NCDs such as cardiovascular disease, type 2 diabetes and obesity [59]. Similarly, the Dietary Guidelines for Americans (DGA) 2020–2025 clearly discouraged sugary beverages for infants and toddlers under two years of age (i.e., sugar-sweetened beverages, regular soda, juice drinks [not 100% fruit juice]; sport drinks, and flavored water with sugar should not be given to children younger than age two) [109]. However, less specific messaging was provided for older children, teens and adults by stating that “added sugars—less than 10 percent of calories per day starting at age 2” and to “limit foods and beverages higher in added sugars”. [109]. Additional research is needed to understand if specific recommendations targeted towards vulnerable populations are an effective strategy to promote healthy dietary patterns.

### 4.2. Healthy Hydration Recommendations among the WHO Regions

This study determined countries within the PAHO/WHO Americas region to have the highest HHR scores to promote healthy hydration compared to other regions demonstrated by an average score of 9 out of 12. This average represented the highest HHR scores compared to the other WHO regions and falls within a high score based on our pre-defined coding framework.

The HHR score among the WHO Africa and Western Pacific Region countries was determined to be low demonstrated by a score of 3.1 and 3.5 out of 12, respectively. Among the 21 countries in the WHO African Region that enacted or updated sugary beverage tax or levy legislation since 2000, eight countries (38%) have published FBDGs [55,56,57,58,59,60,61,62]. Among the 19 countries within the WHO Western Pacific Region that enacted a sugary beverages tax or levy legislation, seven countries (37%) had published FBDGs [119,120,121,122,123,124,125]. Because countries without published FBDGs were included in the HHR score, the WHO Africa and Western Pacific Regions resulted in a lower HHR score compared to the other regions. Moreover, this study did not assess country’s current public health and economic status which could influence the HHR scores and results of this study.

This evaluation showed that the regions in which most countries that enacted sugary beverage legislation had published FBDGs had higher HHR scores (Table 3). These results could be explained by the varying focus on strategies to mitigate obesity and NCD risks across regions due to current public health agendas and priorities. National governments should synergize sugary beverage tax legislation with FBDGs that provide clear, accessible, justified, actionable, specific, and visual messages to encourage healthy hydration.

### 4.3. Implications for Promoting Healthy Hydration to Reduce Sugary Beverage Health Risks

Policy coherence may have three dimensions including: horizontal (i.e., across settings and sectors), vertical (i.e., across levels of governance), and temporal (i.e., across time frames) [129,130]. Within the context of food systems, policy coherence must be evaluated for synergies and conflicts among the policy objectives, policy tools or instruments (hard or soft), implementation practices across settings and sectors, and outcomes and impacts [131]. Policy coherence evaluations could also examine whether and how decision-makers align policies, programs and practices for the production, storage, distribution, processing, packaging, retailing and marketing of foods and beverages consistent with national FBDG recommendations [132]. Many countries have developed FBDGs to improve the population’s diet and health; however, few countries have used FBDGs consistently to inform comprehensive food and nutrition policies, or have effectively implemented their FBDG recommendations [32,133].

To achieve *horizontal policy coherence*, policymakers must take a food systems approach to ensure that national FBDGs guide legislation that aligns with healthy hydration policies, programs and practices across settings and sectors to encourage water availability, access and use; and discourage sugary beverage availability, affordability, marketing and consumption to diverse populations [31,129,130]. This study showed that 56 countries had sugary beverage tax or levy legislation and healthy hydration messages in published FBDGs; however, 35 countries had no national FBDGs and therefore, no healthy hydration messages. It is unclear whether the 56 countries had used the FBDGs to inform the development or implementation of the sugary beverage tax legislation. That question was beyond the scope of this study.

To achieve *vertical policy coherence*, policymakers must coordinate and harmonize efforts at national, sub-national jurisdictional (state or province), and local levels [129,130]. Among the 93 countries that had enacted sugary beverage tax legislation, Canada and the U.S. were the only countries that lacked vertical policy coherence. This is because while their FBDG recommendations encouraged water and discouraged sugary beverage consumption, these countries did not have national sugary beverage tax legislation that applied to all states and provinces. Canada’s government-endorsed FBDGs resulted in a high HHR score (10 of 12) by including key messages that encouraged water and discouraged sugary beverages [95]. Only two Canadian jurisdictions, British Colombia and Newfoundland and Labrador, had enacted sugary beverage taxes [22,42].

The DGA 2020–2025 resulted in a medium HHR score (7 of 12) due to lacking explicit language that was accessible, actionable, specific, and visual messages that encouraged water as the healthy default beverage despite the inclusion of clear, accessible, actionable, and specific messages that discouraged consuming sugary beverages [109]. While the DGA 2020–2025 recommended limiting sugary beverages through a key message and guideline, the default beverage in the graphic FBDG is milk not water. Moreover, the U.S. government has not enacted national legislation to discourage sugary beverage availability, affordability, marketing, and consumption. FBDGs are an important public policy tool, and governments should align their dietary recommendations with a portfolio of supportive policies and synergistic strategies from national to local levels to promote population health.

To achieve *temporal policy coherence*, policymakers must develop and implement actions across government departments and agencies to create synergies that achieve objectives to maximize opportunities, minimize inconsistencies and use resources to support the collective impact of policies for populations over time [31,129,130]. The results from this study suggested a lack of temporal policy coherence among countries with FBDGs published almost two decades ago compared to more recent FBDG recommendations. For instance, Thailand received a healthy hydration score of one. Although Thailand’s FBDGs did not include any specific language encouraging water or discouraging sugary beverages there was an added sugar recommendation that could be applied to beverages [116]. Similarly, Croatia’s Dietary Guidelines were published in 2002 and lacked specific language about healthy hydration [74]. Croatia and Thailand had implemented national sugary beverage tax legislation in 2020 and 2017, respectively [21,22,42].

In addition to discouraging sugary beverages through technical and graphic FBDGs, governments should also address the health risks of diet drinks sweetened with non-sugar sweeteners (NSS) and alcoholic drinks. An analysis of recommendations in the national FBDG documents for NSS and alcoholic drinks was outside of the scope of this study. In 2023, the WHO published a statement that no amount of alcohol intake is safe for human health based on a series of papers published in the Lancet Public Health [134]. The WHO also published a statement in 2023 that the health risks associated with the frequent consumption of NSS beverages were unclear, and issued provisional recommendation that discouraged the use of NSS additives for weight management, and encouraged people to reduce the overall sweetness of their diet starting early in life [135,136]. Governments must consider the types of NSS additives in beverages available in the marketplace and the target population when developing healthy beverage guidelines across the life course. Finally, the growth of non-dairy beverages and functional beverages require that governments consider these new beverage categories when developing HHR. Future research should examine the extent that these beverage categories are addressed and discouraged across graphic and technical FBDG documents.

Governments and food system stakeholders must implement PSE change strategies to promote healthy hydration and water consumption. A 2023 WHO global report on the use of sugary beverage taxes identified nine countries that have earmarked sugary beverage excise tax revenue toward public health programs including: Azerbaijan, France, Hungary, the Philippines, Panama, Zimbabwe, Nicaragua, Poland, and Portugal [137]. Zimbabwe and Nicaragua’s sugary beverage taxes were identified as untargeted through our evidence search process [21,22]. Among the countries with high HHR scores identified through our evaluation, only France, the Philippines, Portugal, and Panama were identified having used earmarked funds for public health programs [137]. Future research should evaluate the sugary beverage tax policy design and earmarking of revenue among countries with sugary beverage legislation and strong healthy beverage recommendations to document policy coherence.

A 2024 WHO report reinforced the recommendation that member states should use fiscal policies broadly, including sugary beverage taxes combined with subsidies to encourage healthy food and beverages, to promote healthy diets [138]. Aside from national sugary beverage tax legislation, governments can support healthy hydration with PSE strategies that expand access to safe drinking water; front-of-package warning labels to discourage sugary beverages; media campaigns promoting water as the healthy default beverage; remove sugary beverages in schools; and enact mandatory advertisement and marketing regulations to children and adolescents. Future research should evaluate the policy coherence among countries and jurisdictions that have enacted sugary beverage legislation and government policies and programs to reduce sugary beverage intake to promote healthy hydration for populations worldwide.

### 4.4. Study Strengths and Limitations

A strength of this study was that it used a novel evaluation process to evaluate the HHR using six criteria (i.e., message clarity [what], accessibility [where], justification [why], actionability [how], specificity [quantity/frequency], and visual content [image]). The evaluation examined the strength of the HHR scores both within and among countries and jurisdictions, which addressed encouraging water and discouraging sugary beverages through FBDG healthy beverage recommendations to support sugary beverage tax and levy legislation. These results provided valuable insights for decisionmakers to inform policy development, monitoring and evaluation across the WHO regions and countries by providing practical and specific actions to be taken to ensure policy coherence. Another strength was the use of investigator triangulation to confirm the interpretation of the evidence to determine a HHR score.

There were several study limitations. While FBDG messages published in English and Spanish were extracted verbatim by the coinvestigators, we had to rely on FAO translated messages or Google translate and a keyword search for the remaining languages (i.e., Benin, France, Hungary, Latvia, Poland, Portugal, and Romania). Moreover, one document was not published in English, did not allow for a keyword search, and was not available via the FAO website (i.e., Croatia). In addition, the FBDG documents for Gabon and Nepal were not available via the FAO website and the co-investigators had to rely on the key messages displayed through the FAO website. This could result in an inability to capture all the messages in these FBDGs, and linguistic differences and cultural dietary variations may result in omissions in data search or translations of healthy hydration messages. Additionally, while Finland’s FBDGs were published in 2014, we relied on the Nordic Nutrition Recommendations for the comprehensive review due to it being available in the English language and it being used to inform the Finnish Nutrition Recommendations.

Certain indicators in the coding framework could be considered subjective (i.e., the actionability of the message or the accessibility of the message) representing a study limitation. While we used a scale of 0–12 to score the HHR, the presentation of some messages may lead to increased dissemination and adoption of the HHR among populations. However, we used our professional judgement to categorize FBDGs content based on the predetermined coding framework. Multiple investigators independently evaluated the messages and met to resolve different interpretations of the evidence.

While findings were triangulated across several evidence sources, this research did not include all sugary beverage taxes globally. Future research should expand on the policy coherence among countries with sugary beverage tax legislation by documenting government-endorsed FBDGs and healthy beverage recommendations for countries with sugary beverage taxes updated or enacted after 2023. Regional and country public health conditions, priorities, and economic status may impact the importance, necessity, and support to update FBDGs to prevent population consumption of sugary beverages. While this study did not assess individual country’s current public health and economic status, future research should consider the variability between countries resources and support to update FBDGs and HHR.

## 5. Conclusions

Governments must ensure policy coherence to reduce sugary beverage health risks by aligning government-endorsed FBDGs and best buy policies, such as sugary beverage tax and levy legislation. Moreover, governments should enact complementary policies, beyond fiscal policies such as taxation, to enable access to free, safe drinking water to achieve healthy hydration in their nations. Sugary beverage tax and levy legislation are an effective policy approach that is increasing, globally. However, no one policy approach will mitigate sugary beverage consumption and related health risks. Integrated coherent policy solutions are needed to establish healthy hydration patterns and decrease consumption of sugary beverages. While water and/or sugary beverages were addressed across the majority of the reviewed FBDGs, only three healthy beverage recommendations (i.e., Bolivia, Brunei, and Peru) included fully comprehensive guidelines according to our coding framework. Comprehensive FBDGs that include clear, accessible, justified, actionable, specific, and visual messages can be used to promote healthy hydration and guide various policy implementation to decrease sugary beverage consumption. Countries with strong HHR scores identified through this study can guide future development of FBDGs to promote healthy hydration and reduce sugary beverage health risks for populations.

## Figures and Tables

**Table 1:** National FBDGs available across countries with targeted sugary beverage taxes or levies across the six WHO regions.

WHO Region Countries with Targeted Sugary Beverage Tax Legislation Enacted or Updated since 2000	SSB Tax or Levy (Year Enacted and/or Updated)	Technical FBDG (Year)
**WHO Africa Region (*n* = 21 countries)**		
Benin	SSB tax (2011, updated 2021)	Benin’s Dietary Guidelines (2015) *
Burkina Faso	SSB tax (1995, updated 2023)	N/A
Cabo Verde	SSB tax (2019)	N/A
Central African Republic	SSB tax (2019)	N/A
Côte d’Ivoire	SSB tax (2018)	N/A
Democratic Republic of the Congo	SSB tax (2018)	N/A
Ethiopia	SSB tax (2003, updated 2020)	Ethiopia: Food-Based Dietary Guidelines (2022)
Equatorial Guinea	SSB tax (2020)	N/A
Eritrea	SSB tax (2001)	N/A
Gabon	SSB tax (2013)	National Dietary Guidelines and Recommendations for Healthy Diets—Gabon (2021) **
Ghana	SSB tax (2014, updated 2023)	Ghana: National Food-Based Dietary Guidelines (2023)
Mali	SSB tax (2005)	N/A
Mauritius	SSB tax (2013, updated 2022)	N/A
Mozambique	SSB tax (2017)	N/A
Niger	SSB tax (2015)	N/A
Nigeria	SSB tax (2021)	Food-Based Dietary Guidelines for Nigeria—A Guide to Healthy Eating (2001)
Sao Tome and Principe	SSB tax (1976, updated 2017)	N/A
Seychelles	SSB tax (2019)	The Seychelles Dietary Guidelines (2006)
South Africa	SSB levy (2018)	Food-Based Dietary Guidelines for South Africa (2013)
Togo	SSB tax (2019)	N/A
Zambia	SSB tax (2018)	Zambia Food-Based Dietary Guidelines Technical Recommendations (2021)
**WHO Eastern Mediterranean Region (*n* = 8 countries)**		
Bahrain	SSB tax (2017)	The Bahraini Food Based Dietary Guidelines: A Holistic Perspective to Health and Wellbeing (2023)
Morocco	SSB tax (2019)	N/A
Oman	SSB tax (2019, updated 2020)	The Omani Guide to Healthy Eating (2009)
Pakistan	SSB tax (2005, updated 2023)	Pakistan Dietary Guidelines for Better Nutrition (2018)
Qatar	SSB tax (2019)	Qatar Dietary Guidelines (2015)
Saudi Arabia	SSB tax (2017, updated 2019)	Dietary Guidelines for Saudis: The Healthy Food Palm (2012)
Tunisia	SSB tax (2018)	N/A
United Arab Emirates	SSB tax (2017, updated 2019)	United Arab Emirates Dietary Guidelines (2019)
**WHO Regional Office for Europe (*n* = 20 countries)**		
Azerbaijan	SSB tax (2019)	N/A
Belgium	SSB tax (2009, updated 2016)	Dietary Guidelines for Belgian Adult Population (2019)
Croatia	SSB tax (1994, updated 2020)	Dietary Guidelines for Adults (2002) **
Finland	SSB tax (1940, updated 2011)	Finnish Nutrition Recommendations (2014) based on the Nordic Nutrition Recommendations (2012)
France	SSB tax (2012, updated 2018)	50 Tips for Eating Better and Moving More (2023) *
Hungary	SSB tax (2011, updated 2022)	Dietary Guidelines for the Adult Population in Hungary (2004) *
Ireland	SSB tax (2018, updated 2019)	Healthy Food for Life—the Healthy Eating Guidelines (2015–2016)
Isle of Man	SSB levy (2019)	N/A
Latvia	SSB tax (2000, updated 2022)	Dietary Guidelines/Healthy Eating Recommendations for Adults (2020) *
Monaco	SSB tax (2012, updated 2018)	N/A
Montenegro	SSB tax (2001)	N/A
Poland	SSB tax (2021)	Healthy Eating Recommendations: Plate of Healthy Eating (2020) *
Portugal	SSB tax (2017, updated 2018)	Food Wheel Guide: A Guide for Daily Food Choices! (2016) *
Romania	SSB tax (2023)	Guidelines for a Healthy Diet (2006) *
Russian Federation	SSB tax (2023)	N/A
Spain	SSB tax (2021)	Healthy and Sustainable Dietary Recommendations Supplemented with Physical Activity Recommendations for the Spanish Population (2022)
	Catalonia, Spain; SSB tax (2017)	
Saint Helena	SSB tax (2014, updated 2018)	N/A
Tajikistan	SSB tax (2018)	N/A
Türkiye	SSB tax (2002, updated 2017)	Dietary Guidelines for Turkey (2006)
United Kingdom	SSB levy (2018)	Eatwell Guide (2016)
**Pan American Health Organization (PAHO)/WHO Americas Region (*n* = 18 countries)**		
Barbados	SSB tax (2015, updated 2022)	Food-Based Dietary Guidelines for Barbados (2017)
Bermuda	SSB tax (2018, updated 2023)	Eat Well Bermuda (2017)
Bolivia	SSB tax (2016, updated 2022)	Food-Based Dietary Guidelines for the Bolivian Population (2014) ***
Canada	British Columbia, Canada; SSB tax (2021)	Canada’s Dietary Guidelines (2019)
	Newfoundland and Labrador, Canada; SSB tax (2022)	
Chile	SSB tax (2014)	Food Guidelines for Chile (2022) ***
Colombia	SSB tax (2023)	Food-Based Dietary Guidelines for the Colombian Population Over 2 Years of Age (2020) ***
Dominica	SSB tax (2015)	Dominica Food-Based Dietary Guidelines (2007)
Ecuador	SSB tax (2016)	The Technical Document of the Food-Based Dietary Guidelines of Food of Ecuador (2020) ***
El Salvador	SSB tax (2010)	Dietary Guidelines for Salvadorian Families (2012) ***
Grenada	SSB tax (2023)	Healthy Choices for Healthy Living—Guidelines for Grenadians (2020)
Guatemala	SSB tax (2002)	Dietary Guidelines for Guatemala Recommendations for Healthy Eating (2012) ***
Honduras	SSB tax (2020)	Dietary Guidelines for Honduras Tips for Healthy Eating (2013) ***
Mexico	SSB tax (2014)	Food Guides for the Mexican Population (2023) ***
Panama	SSB tax (1995, updated 2019)	Dietary Guidelines for Panama (2013) ***
Peru	SSB tax (1999, updated 2021)	Dietary Guidelines for the Peruvian Population (2020) ***
Saint Kitts and Nevis	SSB tax (2010)	Food Based Dietary Guidelines for St. Kitts and Nevis (2010)
Saint Vincent and the Grenadines	SSB tax (2007)	Food Based Dietary Guidelines of St. Vincent and the Grenadines (2021)
USA (8 jurisdictions)	Albany, CA; SSB tax (2017)	Dietary Guidelines for Americans (2020)
	Berkeley, CA; SSB tax (2016)	
	Oakland, CA; SSB tax (2017)	
	San Francisco, CA; SSB tax (2018)	
	Seattle, WA; SSB tax (2018)	
	Boulder, CO; SSB tax (2017)	
	Navajo Nation; SSB tax (2015, updated 2020)	
	Philadelphia, PA; SSB tax (2017)	
**WHO Southeast Asia Region (*n* = 7 countries)**		
Bangladesh	SSB tax (2012)	Dietary Guidelines for Bangladesh (2013)
India	SSB tax (2017)	Dietary Guidelines for Indians—A Manual (2011)
Maldives	SSB tax (2017, updated 2020)	Food Based Dietary Guidelines for Maldives (2019)
Nepal	SSB tax (2002, updated 2022)	Food-Based Dietary Guidelines for Nepalese (2012) **
Sri Lanka	SSB tax (2018, updated 2020)	Food Based Dietary Guidelines for Sri Lankans—Practitioner’s Handbook (2021)
Thailand	SSB tax (2017)	Food-Based Dietary Guideline for Thai (1998, Second Edition 2007)
Democratic Republic of Timor-Leste	SSB tax (2023)	N/A
**WHO Western Pacific Region (*n* = 19 countries)**		
American Samoa	SSB tax (2001)	N/A
Brunei (Brunei Darussalam)	SSB tax (2017, updated 2023)	Dietary Guidelines for Healthy Eating Brunei Darussalam (2020)
Cambodia	SSB tax (2003, updated 2023)	Development of Recommended Dietary Allowance and Food-Based Dietary Guidelines for School-Aged Children in Cambodia (2017)
Cook Islands	SSB tax (2008, updated 2014)	N/A
Fiji	SSB tax (1986, updated 2023)	Food and Health Guidelines for Fiji (2013)
French Polynesia	SSB tax (2004, updated 2020)	N/A
Kiribati	SSB tax (2014)	N/A
Malaysia	SSB tax (2019, updated 2023)	Malaysian Dietary Guidelines (2020)
Marshall Islands	SSB tax (1989, updated 2016)	RMI Guidelines for Healthy Living (2022)
Nauru	SSB tax (2007)	N/A
New Caledonia	SSB tax (2017, updated 2021)	N/A
Niue	SSB tax (1969, updated 2016)	N/A
Northern Mariana Islands	SSB tax (1995, updated 2011)	N/A
Philippines	SSB tax (2018)	Nutritional Guidelines for Filipinos (2012)
Samoa	SSB tax (1984, updated 2018)	N/A
Tonga	SSB tax (2013, updated 2018)	N/A
Tuvalu	SSB tax (2009, updated 2020)	Tuvalu Guidelines for a Healthy Diet and Lifestyle (2021)
Vanuatu	SSB tax (2002, updated 2012, excise implemented 2015)	N/A
Wallis and Futuna	SSB tax (2017)	N/A

Note: Number of countries across the WHO Region in bold. N/A: none available; SSB: sugar-sweetened beverage. Sources: Hattersley and Mandeville 2023 [21], World Bank’s Global Sugary Beverage Tax Database [22], Global Food Research Program’s Sweetened Soft Drinks Tax Maps (November 2023) [42]. * FBDG technical document searched using Google translation of keywords: “water”, “hydration”, “sugar-sweetened beverage”, “sugary beverages”, “beverages” and “drink” and translated key messages and/or guidelines from the FAO website were reviewed. ** FBDG technical document did not allow for keyword searches or was unavailable. Translated key messages and guidelines extracted from FAO website. *** FBDG technical document searched using Spanish translation of keywords: “water”, “hydration”, “sugar-sweetened beverage”, “sugary beverages”, “beverages” and “drink” (i.e., “agua”, “hidratación”, “bebida”, “azucarada”, “bebida endulzada” and “bebida”). The terms “refresco” and “soda” were also searched among the FBDGs published in Spanish.

**Table 2:** Healthy hydration recommendation (HHR) scores for countries with sugary beverage tax legislation across the six WHO regions.

	African Region Country (Score) (FBDG Publication Year)	Eastern Mediterranean Region Country (Score) (FBDG Publication Year)	European Region Country (Score) (FBDG Publication Year)	PAHO/Americas Region Country (Score) (FBDG Publication Year)	Southeast Asia Region Country (Score) (FBDG Publication Year)	Western Pacific Region Country (Score) (FBDG Publication Year)
**Highest HHR Scores (11–12)** (*n* = 13)	Ethiopia **(11)** (2022) Ghana **(11)** (2023)	Pakistan **(11)** (2018)	Belgium **(11)** (2019)	Bolivia **(12)** (2014) Peru **(12)** (2020) Chile **(11)** (2022) Ecuador **(11)** (2020)	Maldives **(11)** (2019) Sri Lanka **(11)** (2021)	Brunei **(12)** (2020) Malaysia **(11)** (2020) Philippines **(11)** (2012)
**High HHR Scores (9–10)** (*n* = 22)	South Africa **(10)** (2013) Seychelles **(9)** (2006)	Qatar **(10)** (2015)	Türkiye **(10)** (2006) France **(9)** (2023) Ireland **(9)** (2015–2016) Portugal **(9)** (2016) United Kingdom **(9)** (2016) Finland **(9)** (2014)	Mexico **(10)** (2023) Panama **(10)** (2013) Saint Vincent and the Grenadines **(10)** (2021) Honduras **(10)** (2013) Canada **(10)** (2019) Guatemala **(10)** (2012) Grenada **(9)** (2020) El Salvador **(9)** (2012)	Bangladesh **(10)** (2013) India **(9)** (2011)	Fiji **(10)** (2013) Marshall Islands **(10)** (2022) Tuvalu **(9)** (2021)
**Medium HHR Scores (4–8)** (*n* = 20)	Zambia **(8)** (2021) Benin **(7)** (2015) Nigeria **(5)** (2001)	Saudi Arabia **(8)** (2012) Oman **(5)** (2009) United Arab Emirates **(4)** (2019) Bahrain **(4)** (2023)	Latvia **(8)** (2020) Hungary **(7)** (2004) Poland **(7)** (2020) Spain **(6)** (2022) Romania **(5)** (2006)	Saint Kitts and Nevis **(8)** (2010) Barbados **(7)** (2017) United States of America **(7)** (2020) Dominica **(6)** (2007) Colombia **(5)** (2020) Bermuda **(5)** (2017)	Nepal **(6)** (2012)	Cambodia **(4)** (2017)
**Lowest HHR Scores (0–3)** (*n* = 3)	Gabon **(3)** (2021)		Croatia **(0)** (2002)		Thailand **(1)** (2007)	
**No FBDG identified** (*n* = 35)	Burkina Faso **(0)** Cabo Verde **(0)** Central African Republic **(0)** Côte d’Ivoire **(0)** Democratic Republic of the Congo **(0)** Equatorial Guinea **(0)** Eritrea **(0)** Mali **(0)** Mauritius **(0)** Mozambique **(0)** Niger **(0)** Sao Tome and Principe **(0)** Togo **(0)**	Morocco **(0)** Tunisia **(0)**	Azerbaijan **(0)** Isle of Man **(0)** Monaco **(0)** Montenegro **(0)** Russian Federation **(0)** Saint Helena **(0)** Tajikistan **(0)**		Democratic Republic of Timor-Leste **(0)**	American Samoa **(0)** Cook Islands **(0)** French Polynesia **(0)** Kiribati **(0)** Nauru **(0)** New Caledonia **(0)** Niue **(0)** Northern Mariana Islands **(0)** Samoa **(0)** Tonga **(0)** Vanuatu **(0)** Wallis and Futuna **(0)**

**Note: Country HHR scores in bold.**

**Table 3:** Countries with high or highest HHR scores (9–12) and model recommendations in FBDGs across the six WHO regions.

WHO Regional Office (Number of Countries across WHO Region)	# Countries with Sugary Beverage Tax Legislation * (Years)	# Countries with FBDG Documents (Years)	Countries with High Healthy Hydration Recommendation Scores (HHR = 9–12) Ranked from Highest to Lowest (Year) *n* = 35 Countries HHR 9–12 (2006–2023)	Countries with the High or Highest Healthy Hydration Scores (HHR = 11–12) and Model Recommendations (Year) *n* = 13 Countries (2012–2023)
Africa Region 47 countries	21 (45%) countries (2001–2023)	8 countries (2001–2023)	**4 countries** Ghana (2023) HHR = 11 Ethiopia (2022) HHR = 11 South Africa (2013) HHR = 10 Seychelles (2006) HHR = 9	**Ghana (2023):** “Drink water regularly” (pg. 34); “It is recommended to limit drinking of sugar-sweetened beverages”. (pg. 34) **Ethiopia (2022):** “Drink 8–10 large glasses of clean water daily”. (pg. 44); “Limit intake of sugar, sweets and soft drinks to below 30 g per day”. (pg. 48)
Eastern Mediterranean Region 21 countries	8 (38%) countries (2017–2023)	6 countries (2009–2023)	**2 countries** Pakistan (2018) HHR = 11 Qatar (2015) HHR = 10	**Pakistan (2018):** “Drink plenty of water each day” (pg. 56); “Reduce sugar intake, and limit intake of soft drinks, confectionaries, bakery products and commercial fruit drinks” (pg. 54)
European Region 53 countries	20 (38%) countries (2011–2023)	13 countries (2006–2022)	**7 countries** Belgium (2019) HHR = 11 Türkiye (2006) HHR = 10 France (2023) HHR = 9 Portugal (2016) HHR = 9 Ireland (2015–2016) HHR = 9 United Kingdom (2016) HHR = 9 Finland (2014) HHR = 9	**Belgium (2019):** “Consume as few drinks with added sugars as possible and choose water instead”. (pg. 63)
Americas Region 35 countries	18 (51%) countries (2002–2023)	18 countries (2007–2023)	**12 countries** Bolivia (2014) HHR = 12 Peru (2020) HHR = 12 Chile (2022) HHR = 11 Ecuador (2020) HHR = 11 Mexico (2023) HHR = 10 St. Vincent and the Grenadines (2021) HHR = 10 Canada (2019) HHR = 10 Honduras (2013) HHR = 10 Panama (2013) HHR = 10 Guatemala (2012) HHR = 10 Grenada (2020) HHR = 9 El Salvador (2012) HHR = 9	**Bolivia (2014):** “Drink daily 6–8 glasses of water complementary to the meals”. (pg. 47); “Avoid excessive consumption of sugar, sweets, carbonated drinks, and alcoholic beverages”. (pg. 47) **Peru (2020):** “Stay healthy by drinking 6 to 8 glasses of water a day”. (pg. 33); “Take care of your health; avoid overweight by reducing the consumption of sugars in your meals and drinks”. (pg. 30) **Chile (2022):** “Drink water several times a day, do not replace it with juices or beverages”. (pg. 36) “Beverages, juices, sports drinks, and energy drinks, for the most part, contain sugar, increasing calories and sugars in your diet, promoting obesity, stimulating appetite, and leading to the onset of dental cavities and metabolic diseases such as diabetes and osteoporosis”. (pg. 38) **Ecuador (2020):** “Let’s drink eight glasses of safe water daily for proper body function”. (pg. 154); “Let’s protect our health: let’s avoid the consumption of processed products, fast food, and sugary drinks”. (pg. 154)
Southeast Asia 11 countries	7 (64%) countries (2012–2023)	6 countries (1998–2021)	**4 countries** Sri Lanka (2021) HHR = 11 Maldives (2019) HHR = 11 Bangladesh (2013) HHR = 10 India (2011) HHR = 9	**Sri Lanka (2021):** “Water is the healthiest drink: Drink 8 to 10 glasses (1.5–2.0 L) throughout the day”. (pg. 59); “Limit sugary drinks, biscuits, cakes, sweets and sweeteners”. (pg. 55) **Maldives (2019):** “Drink plenty of water and choose water over sugary drinks” (pg. 13)
Western Pacific 37 countries	19 (51%) countries (2001–2023)	7 countries (2012–2022)	**6 countries** Brunei (2020) HHR = 12 Malaysia (2020) HHR = 11 Philippines (2012) HHR = 11 Fiji (2013) HHR = 10 Marshall Islands (2022) HHR = 10 Tuvalu (2021) HHR = 9	**Brunei (2020):** “Drink at least eight glasses of water a day”. (pg. 102); “Limit intake of sugar-sweetened beverages”. (pg. 102) **Malaysia (2020):** “Drink plenty of water daily”. (pg. 196); “Limit sugar intake in foods and beverages”. (pg. 178) **Philippines (2012):** “Consume safe foods and water to prevent diarrhea and other food and water-borne diseases”. (pg. 73); “Soft drinks are refreshing beverages that provide water and energy, but like coffee and tea should be consumed in moderation”. (pg. 25)

# = Number; * Sugary beverage tax legislation year was the most current or updated for each country by region (2000–2023). High Healthy Hydration Recommendation Scores (HHR) (9–12) were based on six criteria including: (1) message clarity (what), (2) accessibility (where), (3) justification (why), (4) actionability (how), (5) specificity (amount or quantity and frequency), and (6) visual representation or image content in the graphic FBDG documents. Note: the number of countries with High HHR are bolded; the name of countries with HHR scores 11–12 are bolded.

## Data Availability

The data used in this study are openly available in the public domain from the FAO [43], World Bank [22], and WHO [20,41] websites.

## Supplementary Materials

The following supporting information can be downloaded at: https://www.mdpi.com/article/10.3390/nu16142264/s1, Table S1. Coding Scheme Used to Assess the FBDG for Healthy Hydration Recommendations Across the Six WHO Regions. Table S2. Detailed Table of Countries with Sugary Beverage Taxes or Levies Across the Six WHO regions and National FBDGs. Table S3. Graphic FBDG for Countries that Implemented Sugar Beverage Taxes or Levies (1998–2023). Table S4. FBDG Healthy Hydration Recommendations for Countries with Sugary Beverage Tax Legislation in the WHO African Region. Table S5. FBDG Healthy Hydration Recommendations for Countries with Sugary Beverage Tax Legislation in the WHO Eastern Mediterranean Region. Table S6. FBDG Healthy Hydration Recommendations for Countries with Sugary Beverage Tax Legislation in the WHO European Region. Table S7. FBDG Healthy Hydration Recommendations for Countries with Sugary Beverage Tax Legislation in the PAHO/WHO Americas Region. Table S8. FBDG Healthy Hydration Recommendations for Countries with Sugary Beverage Tax Legislation in the WHO Southeast Asia Region. Table S9. FBDG Healthy Hydration Recommendations for Countries with Sugary Beverage Tax Legislation in the WHO Western Pacific Region.
