# Supplementary material for: An Evaluation of Healthy Hydration Recommendations for 93 Countries with Sugary Beverage Tax Legislation Globally, 2000–2023
Source: Nutrients. 2024 Jul 13;16(14):2264. doi: 10.3390/nu16142264 (PMC11279664; doi:10.3390/nu16142264)
Supplement: Supplementary file 1 [file nutrients-16-02264-s001.zip › nutrients-3060040-supplementary.pdf]

An Evaluation of Healthy Hydration Recommendations for 93 Countries with Sugary Beverage Tax Legislation Globally, 2000-2023

Nicole Leary[1,*], Molly K. Parker[1], Sofía Rincón Gallardo Patiño[2] and Vivica I. Kraak[1]

Supplemental Tables 1 - 9

Supp Table 1. Coding Scheme Used to Assess the FBDG for Healthy Hydration Recommendations Across the Six WHO Regions.
Supp Table 2. Detailed Table of Countries with Sugary Beverage Taxes or Levies Across the Six WHO regions and National FBDGs.
Supp Table 3. Graphic FBDG for Countries that Implemented Sugar Beverage Taxes or Levies (1998-2023).
Supp Table 4. FBDG Healthy Hydration Recommendations for Countries with Sugary Beverage Tax Legislation in the WHO African Region.
Supp Table 5. FBDG Healthy Hydration Recommendations for Countries with Sugary Beverage Tax Legislation in the WHO Eastern Mediterranean Region.
Supp Table 6. FBDG Healthy Hydration Recommendations for Countries with Sugary Beverage Tax Legislation in the WHO European Region.
Supp Table 7. FBDG Healthy Hydration Recommendations for Countries with Sugary Beverage Tax Legislation in the PAHO/WHO Americas Region.
Supp Table 8. FBDG Healthy Hydration Recommendations for Countries with Sugary Beverage Tax Legislation in the WHO Southeast Asia Region.
Supp Table 9. FBDG Healthy Hydration Recommendations for Countries with Sugary Beverage Tax Legislation in the WHO Western Pacific Region.

Abbreviations and Acronyms
FAO: Food and Agriculture Organization; FBDG: food-based dietary guidelines; Fl: fluid; L: litre/liter; N/A: not available; Oz: ounce
PAHO: Pan American Health Organization; SSB: sugary sweetened beverages; WHO: World Health Organization

Supplemental Table 1. Coding Scheme Used to Assess the FBDG for Healthy Hydration Recommendations Across the Six WHO Regions.

Category	Code	Variable	Possible Values	Points
**Message Clarity**	**What**	Do the FBDGs include healthy beverage recommendations to drink water?	No, there is no mention of water in the FBDGs	0
			Yes, the FBDGs encourage water consumption	1
	**What**	Do the FBDGs include healthy beverage recommendations to avoid sugary beverages?	No, there is no discouragement of sugary beverages in the FBDGs	0
			Yes, the FBDGs discourage sugary beverage consumption	1
**Accessibility**	**Where**	Do the FBDGs include healthy beverage recommendations to drink water presented in user-friendly ways (i.e., highlighted, bolded, and in text box) or as one of the key messages/guidelines?	No, there is no mention of water in the FBDGs<br><br>No, the water recommendations are not presented in user-friendly ways (i.e., highlighted, bolded, and in text box)<br><br>No, the FBDGs do not include healthy beverage recommendations to drink water as one of the key messages/guidelines	0
			Yes, the FBDGs include healthy beverage recommendations to drink water presented in user-friendly ways (i.e., highlighted, bolded, and in text box)<br><br>Yes, the FBDGs include healthy beverage recommendations to drink water as one of the key messages/guidelines	1
	**Where**	Do the FBDGs include healthy beverage recommendations to limit sugary beverages in user-friendly ways (i.e., highlighted, bolded, and in text box) or as one of the key messages/guidelines?	No, there is no mention of sugary beverages in the FBDGs<br><br>No, the sugary beverage recommendations are not presented in user-friendly ways (i.e., highlighted, bolded, and in text box)	0

			No, the FBDGs do not include healthy beverage recommendations to limit sugary beverages as one of the key messages/guidelines	
			Yes, the FBDGs include healthy beverage recommendations to limit sugary beverages presented in user-friendly ways (i.e., highlighted, bolded or in text box)<br><br>Yes, the FBDGs include healthy beverage recommendations to limit sugary beverages as one of the key messages/guidelines	1
**Justification**	**Why**	Do the FBDGs include a rationale for water consumption?	No, there is no mention of water in the FBDGs<br><br>No, the FBDGs do not include a rationale with reasons to drink water	0
			Yes, the FBDGs include a rationale with reasons to drink water	1
	**Why**	Do the FBDGs include a rationale for limiting sugary beverages?	No, there is no mention of sugary beverages in the FBDGs<br><br>No, the FBDGs do not include a rationale with reasons to limit sugary beverages	0
			Yes, the FBDGs include a rationale with reasons to limit sugary beverages	1
**Actionability**	**How**	Do the FBDGs include actionable recommendations to consume water? Do the recommendations include actionable words (i.e., drink, choose, consume, limit, make, etc.)?	No, there is no mention of water in the FBDGs<br><br>No, the FBDGs do not include actionable recommendations to consume water	0
			Yes, the FBDGs do include actionable recommendations to consume water	1
	**How**	Do the FBDGs include actionable recommendations to limit sugary	No, there is no mention of sugary beverages in the FBDGs	0

		beverages? Do the recommendations include actionable words (i.e., drink, choose, consume, limit, make, etc.)?	No, the FBDGs do not include actionable recommendations to consume water	
			Yes, the FBDGs do include actionable recommendations to consume water	1
**Specificity**	**Quantity/Frequency**	Do the FBDGs include measurable recommendations for water (i.e., 8 glasses per day)?	No, there is no mention of water in the FBDGs<br><br>No, the FBDGs do not include measurable recommendations for water	0
			Yes, the FBDGs include measurable recommendations for water	1
	**Quantity/Frequency**	Do the FBDGs include measurable recommendations for limiting sugary beverages?	No, there is no mention of sugary beverages in the FBDGs<br><br>No, the FBDGs do not include measurable recommendations for sugary beverages	0
			Yes, the FBDGs include measurable recommendations for sugary beverages	1
**Visual Representation**	**FBDG image content**	Does the FBDG graphic image include water?	No, there is no FBDG graphic image<br><br>No, there is no visual representation of water in the FBDG image	0
			Yes, there is a visual representation of water in the FBDG image	1
	**FBDG image content**	Does the FBDG graphic image discourage sugary beverage?	No, there is no FBDG graphic image<br><br>No, there is not a visual discouraging sugary beverages in the FBDG image	0
			Yes, there is a visual discouraging sugary beverages in the FBDG image	1

Supp Table 2. Detailed Table of countries with sugary beverage taxes or levies across the six WHO regions and National FBDGs

**WHO Region** Countries with sugary beverage tax or legislation enacted or updated since 2000	***SSB tax or levy** (year enacted and/or updated; amount in USD)	**Technical FBDG** (year)
**WHO Africa Region (*n*=21 countries)**		
Benin	SSB tax (2011, updated 2021); National 20% ad valorem excise tax on energy drinks, imported fruit juices and mineral waters; 7% ad valorem excise tax on non-alcoholic beverages except plain mineral water	Benin's Dietary Guidelines (2015)
Burkina Faso	SSB tax (1995, updated 2023); National 50% ad valorem excise tax on energy drinks, 15% ad valorem excise tax on other sugary beverages	N/A
Cabo Verde	SSB tax (2019); National 10% ad valorem excise tax on waters containing added sugar excluding juices and milk-based drinks	N/A
Central African Republic	SSB tax (2019); National 10% ad valorem excise tax on all imported non-alcoholic beverages excluding plain water	N/A
Côte d'Ivoire	SSB tax (2018); National 20% ad valorem excise tax on all sugary beverages	N/A
Democratic Republic of the Congo	SSB tax (2018); National 10% ad valorem excise tax on fruit and vegetable juices with or without added sugar, lemonades, and other sugary drinks	N/A
Ethiopia	SSB tax (2003, updated 2020); National 25% ad valorem excise tax on sugary beverages	Ethiopia: Food-Based Dietary Guidelines (2022)
Equatorial Guinea	SSB tax (2020); National specific excise tax of XAF 100/L ($0.16 USD) on caloric non-alcoholic beverages with added sugar, syrup, or energy	N/A
Eritrea	SSB tax (2001); National mixed excise tax of 15% ad valorem or specific tax of Nfa 0.30, whichever is highest, on sugary beverages	N/A
Gabon	SSB tax (2013); National 5% ad valorem excise tax on sugary beverages	National Dietary Guidelines and Recommendations for Healthy Diets – Gabon (2021)
Ghana	SSB tax (2014, updated 2023); National 20% ad valorem excise tax on sugary beverages	Ghana: National Food-Based Dietary Guidelines (2023)

Mali	SSB tax (2005); National 10% ad valorem excise tax on carbonated sweetened waters and 12% ad valorem excise tax on juices	N/A
Mauritius	SSB tax (2013, updated 2022); National specific excise tax of MUR 0.06/g sugar ($0.0013 USD) on sugary beverages	N/A
Mozambique	SSB tax (2017); National specific excise tax of 1 Mt/L ($0.016) on waters containing added sugar	N/A
Niger	SSB tax (2015); National 15% ad valorem excise tax on non-alcoholic beverages excluding plain water	N/A
Nigeria	SSB tax (2021); National specific excise tax of NGN 10/L ($0.02 USD) on non-alcoholic, carbonated and sugary beverages	Food-Based Dietary Guidelines for Nigeria – A Guide to Healthy Eating (2001)
Sao Tome and Principe	SSB tax (1976, updated 2017); National 10-20% ad valorem excise tax on sugary beverages	N/A
Seychelles	SSB tax (2019); National specific excise tax of SCR 4/L ($0.31 USD) on sugary beverages containing >5g sugar per 100ml	The Seychelles Dietary Guidelines (2006)
South Africa	SSB levy (2018); National specific excise tax of ZAR 0.021 over 4 g sugar per 100 ml ($0.001 USD) in sugary beverages	Food-Based Dietary Guidelines for South Africa (2013)
Togo	SSB tax (2019); National 5% ad valorem excise tax on sugary beverages	N/A
Zambia	SSB tax (2018); National specific excise tax of ZMWO 3/L ($0.12 USD) on non-alcoholic sugary beverages	Zambia Food-based Dietary Guidelines Technical Recommendations (2021)
**WHO Eastern Mediterranean Region (*n*=8 countries)**		
Bahrain	SSB tax (2017); National 50% ad valorem excise tax on carbonated soft drinks and 100% ad valorem excise tax on energy drinks	The Bahraini Food Based Dietary Guidelines: A Holistic Perspective to Health and Wellbeing (2023)
Morocco	SSB tax (2019); National specific excise tax of MAD 0.15-0.70/L ($0.015-0.07 USD) varies based on sugary beverage	N/A
Oman	SSB tax (2019, updated 2020); National 50% ad valorem excise tax on ready-to-drink beverages that contain added sugar and 100% ad valorem excise tax on energy drinks	The Omani Guide to Healthy Eating (2009)
Pakistan	SSB tax (2005, updated 2023); National 20% ad valorem excise tax on waters containing added sugar	Pakistan Dietary Guidelines for Better Nutrition (2018)
Qatar	SSB tax (2019); National 50% ad valorem excise tax on carbonated sugary drinks and 100% ad valorem excise tax on energy drinks	Qatar Dietary Guidelines (2015)

Saudi Arabia	SSB tax (2017, updated 2019); National 50% ad valorem excise tax on sugary beverages and 100% ad valorem excise tax on energy drinks	Dietary Guidelines for Saudis: The Healthy Food Palm (2012)
Tunisia	SSB tax (2018); National 25% ad valorem excise tax on waters containing added sugar, fruit and vegetable drinks, coffees and teas and 10% ad valorem excise tax on beverages under cocoa-based preparations	N/A
United Arab Emirates	SSB tax (2017, updated 2019); National 50% ad valorem excise tax on all soft drinks excluding sparkling water and 100% ad valorem excise tax on energy drinks	United Arab Emirates Dietary Guidelines (2019)
**WHO Regional Office for Europe (*n*=20)**		
Azerbaijan	SSB tax (2019); National specific excise tax of ANZ 3/L ($1.76 USD) on energy drinks	N/A
Belgium	SSB tax (2009, updated 2016); National specific excise tax of €0.068/L ($0.07USD) (varies based on sugary beverage)	Dietary Guidelines for Belgian Adult Population (2019)
Croatia	SSB tax (1994, updated 2020); National tiered excise tax €1.33-7.96/hl ($0.014-0.086/L USD)	Dietary Guidelines for Adults (2002)
Finland	SSB tax (1940, updated 2011); National specific excise tax of €0.32/L ($0.34 USD) on sugary soft drinks	Finnish Nutrition Recommendations (2014) based on the Nordic Nutrition Recommendations (2012)
France	SSB tax (2012, updated 2018); National specific excise tax up to €0.24 per L ($0.21 USD) (varies based on sugary beverage)	50 Tips for Eating Better and Moving More (2023)
Hungary	SSB tax (2011, updated 2022); National specific excise tax of HUF 8-390/L ($0.02-1.05 USD) (varies based on sugary beverage)	Dietary Guidelines for the Adult Population in Hungary (2004)
Ireland	SSB tax (2018, updated 2019); National specific excise tax of €0.20-0.30/L ($0.21-0.32 USD) (varies based on sugary beverage)	Healthy Food for Life – the Healthy Eating Guidelines (2015-2016)
Isle of Man	SSB levy (2019); National specific excise tax of €0.18-0.24/L ($0.20-0.37) (varies based on sugary beverage)	N/A
Latvia	SSB tax (2000, updated 2022); National tiered excise tax of €0.074-0.14/L ($0.08-0.15 USD) (varies based on sugar content)	Dietary Guidelines/Healthy Eating Recommendations for Adults (2020)
Monaco	SSB tax (2012, updated 2018); National specific excise tax of €3.0-24.34 per hectolitre (varies based on sugar content) plus €2.07 per hl for each additional kg	N/A
Montenegro	SSB tax (2001); National specific excise tax €10 per hectolitre on carbonated soft drinks with added sugar	N/A

Poland	SSB tax (2021); National specific excise tax of PLN 0.05-0.5/L ($0.01-0.11) (varies based on sugary beverage)	Healthy Eating Recommendations: Plate of Healthy Eating (2020)
Portugal	SSB tax (2017, updated 2018); National tiered excise tax of €0.01-0.20/L ($0.01-0.21 USD) (varies based on sugar content)	Food Wheel Guide: A Guide for Daily Food Choices! (2016)
Romania	SSB tax (2023); National 19% ad valorem value added tax on beverages excluding juices, milk, unsweetened waters	Guidelines for a Healthy Diet (2006)
Russian Federation	SSB tax (2023); National specific excise tax of 7 Rubles/L ($0.074 USD) on drinks sweetened with added sugar > 5g per 100ml excluding juice and milk-based drinks	N/A
Spain	SSB tax (2021); National 21% ad valorem value added tax on sugary beverages	Healthy and Sustainable Dietary Recommendations Supplemented with Physical Activity Recommendations for the Spanish Population (2022)
	Catalonia, Spain; SSB tax (2017): State/province specific excise tax of €0.10-0.15/L ($0.11-0.16 USD) (varies based on sugary beverage content)	
Saint Helena	SSB tax (2014, updated 2018); National specific excise tax of €1.00/L ($1.06 USD) on carbonated beverages, juices, and concentrates containing ≥15 g sugar/L	N/A
Tajikistan	SSB tax (2018); National specific excise tax of €0.09/L ($0.096 USD) on water-based sugary beverages	N/A
Türkiye	SSB tax (2002, updated 2017); National 35% ad valorem excise tax on sugary beverages	Dietary Guidelines for Turkey (2006)
United Kingdom	SSB levy (2018); National specific excise tax of €0.18-0.24/L ($0.19-0.26) (varies based on sugar content)	Eatwell Guide (2016)
**PAHO/WHO Americas Region (*n*=18 countries)**		
Barbados	SSB tax (2015, updated 2022); National 20% ad valorem excise tax on sugary beverages	Food-Based Dietary Guidelines for Barbados (2017)
Bermuda	SSB tax (2018, updated 2023); National 25-75% ad valorem import tax on sugary beverages (varies based on sugar content)	Eat Well Bermuda (2017)
Bolivia	SSB tax (2016, updated 2022); National specific excise tax of Bs. 5.36/L ($0.77 USD) on energy drinks and Bs. 0.48 ($0.069 USD) on other sugary beverages	Food-based Dietary Guidelines for the Bolivian Population (2014)
Canada	British Columbia, Canada; SSB tax (2021); State/providence 7% ad valorem sales tax on carbonated sugary beverages	Canada's Dietary Guidelines (2019)
	Newfoundland and Labrador, Canada; SSB tax (2022); State/providence specific excise tax of CAD$0.20/L ($0.14) on sugary beverages	

Chile	SSB tax (2014); National 10-18% ad valorem excise tax (varies based on sugar content)	Food Guidelines for Chile (2022)
Colombia	SSB tax (2023); National tiered tax Col$0-35 per 100 mL ($0-0.009/L) (varies based on sugary beverage)	Food-Based Dietary Guidelines for the Colombian Population Over 2 Years of Age (2020)
Dominica	SSB tax (2015); National mixed specific and 10% ad valorem excise tax: $0.20/L on carbonated soft drinks, $0.63/L on malt beverages, 10% on energy and other drinks excluding plain water and juices	Dominica Food-Based Dietary Guidelines (2007)
Ecuador	SSB tax (2016); National mixed specific and 10% ad valorem excise tax on sugary beverages <25g sugar - $0.18 per 100g sugar >25g sugar	The Technical Document of the Food-Based Dietary Guidelines of Food of Ecuador (2020)
El Salvador	SSB tax (2010); National mixed specific and 10% ad valorem excise tax and USD$0.20 per L on energy drinks; 10% on carbonated and energy drinks; 5% on sports drinks, and juices	Dietary Guidelines for Salvadorian Families (2012)
Grenada	SSB tax (2023); National 20% ad valorem value added tax on sugary beverages	Healthy Choices for Healthy Living – Guidelines for Grenada (2020)
Guatemala	SSB tax (2002); National specific excise tax of GTQ$ 0.10-0.18 ($0.01-0.023 USD) (varies based on sugary beverage)	Dietary Guidelines for Guatemala Recommendations for healthy eating (2012)
Honduras	SSB tax (2020); National specific excise tax HNL 0.82/L ($0.033 USD) on carbonates and other soft drinks, excluding 100% juices	Dietary Guidelines for Honduras Tips for Healthy Eating (2013)
Mexico	SSB tax (2014); National mixed specific and 25% ad valorem excise tax; 1 peso/L ($0.05) on all sugary beverages; additional 25% on energy drinks	Food Guides for the Mexican Population (2023)
Panama	SSB tax (1995, updated 2019); National 5-7% ad valorem excise tax on sugary beverages excluding dairy, natural fruit juices, and drinks with <7.5 g sugar per 100 ml	Dietary Guidelines for Panama (2013)
Peru	SSB tax (1999, updated 2021); National 12-25% ad valorem excise tax applied to all sweetened beverages excluding drinkable yogurts and 100% juice (varies based on sugary beverage)	Dietary Guidelines for the Peruvian Population (2020)
Saint Kitts and Nevis	SSB tax (2010); National 5% ad valorem excise tax on waters containing added sugar	Food-Based Dietary Guidelines for St. Kitts and Nevis (2010)

Saint Vincent and the Grenadines	SSB tax (2007); National 5% ad valorem excise tax on carbonated beverages excluding plain water and fruit juices; 10% on other beverages containing cocoa	Food Based Dietary Guidelines of St. Vincent and the Grenadines (2021)
USA (8 jurisdictions)	Albany, CA; SSB tax (2017); City level specific excise tax of 1 cent/oz on sugary beverages with added caloric sweeteners	Dietary Guidelines for Americans (2020)
	Berkeley, CA; SSB tax (2016); City level specific excise tax of 1 cent/oz on sugary beverages with added caloric sweeteners	
	Oakland, CA; SSB tax (2017); City level specific excise tax of 1 cent/oz on sugary beverages with added caloric sweeteners	
	San Francisco, CA; SSB tax (2018); City level specific excise tax of 1 cent/oz on sugary beverages with added caloric sweeteners	
	Seattle, WA; SSB tax (2018); City level specific excise tax; Manufacturers making over $5 million: US$1.75 per oz on sugary beverages to which one or more added caloric sweeteners has been added and that contains at least 40 calories per 12 oz serving. Manufacturers making over $2 million but less than $5 million: $.01 per oz with certification from the city. Products from manufacturers making $2K or less in annual income are not taxed with certification from the city.	
	Boulder, CO; SSB tax (2017); City level specific excise tax of 2 cents/oz on sugary beverages with at least 5 g of caloric sweetener per 12 fluid oz	
	Navajo Nation; SSB tax (2015, updated 2020); State/province 2% ad valorem sales tax on all sugary beverages (i.e., sweetened with caloric sweeteners, sweetened with non-caloric sweeteners, carbonated or non-carbonated)	
	Philadelphia, PA; SSB tax (2017); City level specific excise tax of 1.5 cents/oz sugary beverages with caloric sugar-based sweetener or artificial sugar substitute	
**WHO Southeast Asia Region (*n*=7 countries)**		
Bangladesh	SSB tax (2012); Domestic: National 25% value added ad valorem tax on carbonated sugary beverages, 35% value added ad valorem tax on energy drinks. Imports: National 150% value added ad valorem tax on sugary beverages	Dietary Guidelines for Bangladesh (2013)
India	SSB tax (2017); National 28% value added ad valorem tax on carbonated water containing added sugar plus 12% on processed and packaged beverages	Dietary Guidelines for Indians – A Manual (2011)
Maldives	SSB tax (2017, updated 2020); National mixed specific and ad valorem import tax of MVR 60.55 MVR/L ($3.92 USD) on energy drinks, 8 MVR/L ($0.52) on soft drinks; 15% on sugary and unsweetened water excluding 100% juices and juice-based drinks, milk-based drinks, and ready to drink sweetened teas and coffees	Food Based Dietary Guidelines for Maldives (2019)

Nepal	SSB tax (2002, updated 2022); National specific excise tax of Rs.11-50/L ($0.082-0.37 USD) (varies based on sugary beverage)	Food-Based Dietary Guidelines for Nepalese (2012)
Sri Lanka	SSB tax (2018, updated 2020); National specific excise tax of LKR 12/L ($0.04 USD) or 30 cents per gram of sugar, excluding first 6g per 100ml, whichever is higher on beverages sugary beverages and energy drinks	Food based dietary guidelines for Sri Lankans – Practitioner's Handbook (2021)
Thailand	SSB tax (2017); National mixed specific excise tax of 1-5 baht/L ($0.03-0.15) and ad valorem excise tax of 10-14% on sugary beverages (varies based on sugar content and sugary beverage)	Food-Based Dietary Guideline for Thai (1998, Second Edition 2007)
Democratic Republic of Timor-Leste	SSB tax (2023); National specific excise tax USD$3 per L on waters containing added sugar or other sweeteners, excluding fruit or vegetable juices	N/A
**WHO Western Pacific Region (*n*=19 countries)**		
American Samoa	SSB tax (2001); National specific excise tax of US$0.15/12oz (US$0.42/L) on carbonated soft drinks	N/A
Brunei	SSB tax (2017, updated 2023); National specific excise tax of BND 4.00 per 10 L ($0.30/L) on sugary beverages	National Dietary Guidelines for Healthy Eating Brunei Darussalam (2020)
Cambodia	SSB tax (2003, updated 2023); National 5-15% ad valorem excise tax (varies based on sugary beverages)	Development of Recommended Dietary Allowance and Food-Based Dietary Guidelines for School-Aged Children in Cambodia (2017)
Cook Islands	SSB tax (2008, updated 2014); National specific excise tax of NZ 9.37 per 1 kg added sugar ($0.0058/g) on sugary beverages excluding juices, milk products, tea, and coffee drinks	N/A
Fiji	SSB tax (1986, updated 2023); National specific excise tax of FJD 0.35/L ($0.16) local production tax on sugary drinks; 32% or FJD 2/L ($0.89) fiscal import tax on carbonated sugary drinks	Food and Health Guidelines for Fiji (2013)
French Polynesia	SSB tax (2004, updated 2020); National tiered specific excise tax of CFP/L 0-85 per L ($0.76 USD) (varies based on sugary beverage)	N/A
Kiribati	SSB tax (2014); National 40% ad valorem excise tax on sugary beverages	N/A
Malaysia	SSB tax (2019, updated 2023); National specific excise tax of MYR 0.40-.47/L ($0.083-0.098 USD) (varies based on sugary beverage)	Malaysian Dietary Guidelines (2020)
Marshall Islands	SSB tax (1989, updated 2016); National mixed specific import tax of US $0.020826/oz on carbonated sugary beverage and 30% ad valorem tax on non-carbonated sugary beverages and artificially sweetened beverages	RMI Guidelines for Healthy Living (2022)

Nauru	SSB tax (2007); National 30% ad valorem import tax on soft drinks containing added sugar, including sweetened juice and milk-based drinks	N/A
New Caledonia	SSB tax (2017, updated 2021); National 22% ad valorem value added tax on sugary beverages	N/A
Niue	SSB tax (1969, updated 2016); National 80% ad valorem import tariff on sugary beverages	N/A
Northern Mariana Islands	SSB tax (1995, updated 2011); US $0.005 per fl oz excise tax on non-alcoholic beverages, excluding milk-based drinks, fruit or vegetable juices, and unsweetened water	N/A
Philippines	SSB tax (2018); National specific excise tax of PHP 6/L ($ 0.10 USD) on sugary beverages; P12 per L ($0.21) on drinks with high-fructose corn syrup	Nutritional Guidelines for Filipinos (2012)
Samoa	SSB tax (1984, updated 2018); National specific excise tax of 52.5 sene/L ($0.19 USD) on waters containing added sugar	N/A
Tonga	SSB tax (2013, updated 2018); National specific tiered excise tax of T$0.05-4/L ($0.021-1.67 USD) (varies based on sugary beverage)	N/A
Tuvalu	SSB tax (2009, updated 2020); National 30% ad valorem excise tax on all soft drinks excluding juices, milk-based drinks, concentrates, and plain water	Tuvalu Guidelines for a Healthy Diet and Lifestyle (2021)
Vanuatu	SSB tax (2002, updated 2012, excise implemented 2015); National specific excise tax of 50 vatu/L excise ($0.40) + 75% ad valorem tariff on soft drinks with added sugar or other sweeteners	N/A
Wallis and Futuna	SSB tax (2017); National 30% ad valorem import tariff on sugary beverages	N/A

*Sugary beverages are broadly defined as beverages low in nutritional quality that contain free or added sugars, such as carbonated and non-carbonated soft drinks (i.e., soda), fruit drinks, sports drinks, and energy drinks. The sugary beverages included/excluded in the SSB may vary by country or jurisdiction.

Fl: fluid; g: grams; L: litre/liter; ml: milliliter; N/A: none available; oz: ounce; SSB: sugar-sweetened beverage.

ANZ: Azerbaijan Manat; BND: Brunei dollar; Bs: Bolivian boliviano; CAD: Canadian dollar; CFP: French Pacific Fran; FJD: Fijian Dollar; GTQ; Guatemalan Quetzal; HNL: Honduran Lempira; HUF: Hungarian Forint; LKR: Sri Lankan Rupees; Mt: Mozambican metical; MUR: Mauritian Rupee; MVR; Maldivian Rufiyaa; MYR: Malaysian Ringgits; Nfa: Eritrean Nakfa; NGN: Nigerian Naira; NZ; New Zealand Dollar; PHP: Philippine peso; PLN: Polish złoty; RM: Malaysian Ringgits; Rs: Indian Rupee; SCR: Seychellois Ruppe; THB: Thai Baht; TShs: Tanzanian Shilling; USD: United States Dollar; XAF: Central African CFA Franc; ZAR: South African rand; ZMWO: Zambian Kwacha

Table adapted from the Hattersley and Mandeville 2023, World Bank's Global SSB Tax Database, Global Food Research Program's Sweetened Soft Drinks Tax Maps (November 2023)

Supplemental Table 3. Graphic Food-Based Dietary Guidelines (FBDG) for Countries that Implemented Sugar Beverage Taxes of Levies (1998-2023).

<table>
<tr><td rowspan="2">WHO African Region*
(n=7)</td><td>Nigeria (2001)
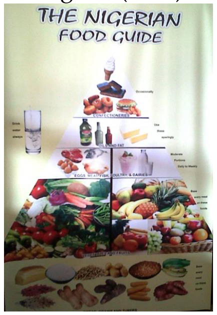
</td><td>Seychelles (2006)
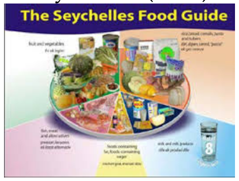
</td><td>South Africa (2013)
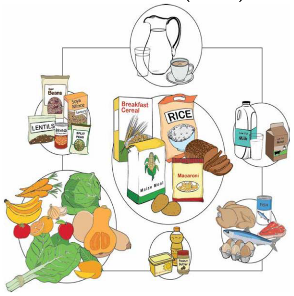
</td><td>Benin (2015)
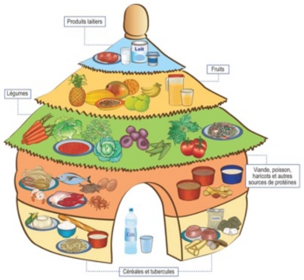
</td></tr>
<tr><td>Zambia (2021)
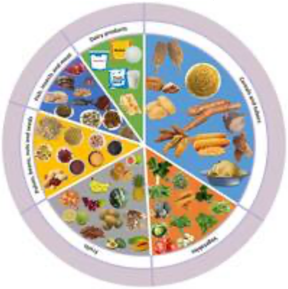</td><td>Ethiopia (2022)
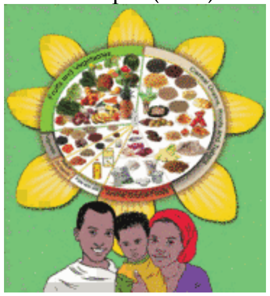</td><td>Ghana (2023)
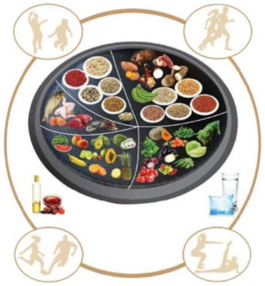</td><td></td></tr>
<tr><td rowspan="2">WHO Eastern Mediterranean Region**
(n=5)
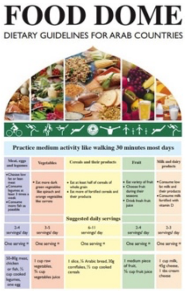
</td><td>Oman (2009)
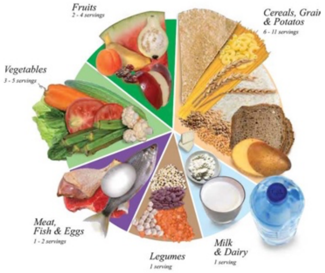
</td><td>Saudi Arabia (2012)
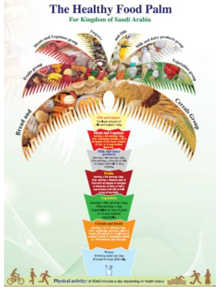
</td><td>Qatar (2015)
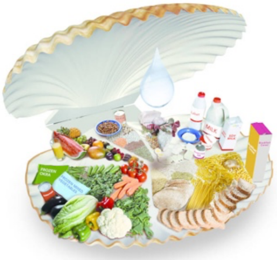</td><td>Pakistan (2018)
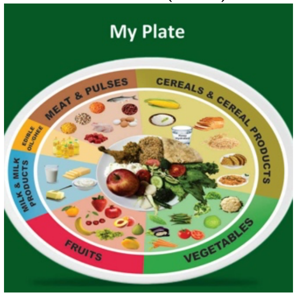
</td></tr>
<tr><td>United Arab Emirates (2019)</td><td></td><td></td><td></td></tr>
</table>

Food Dome for the six Arab Guld Countries (2012)	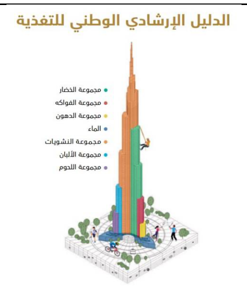			
**WHO European Region*** (*n*=13)**	Croatia (2002)<br>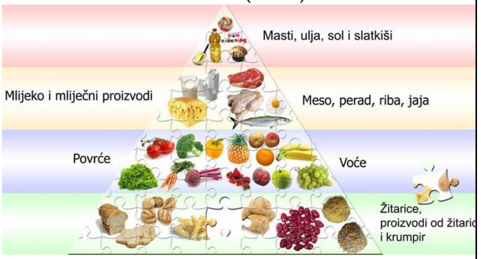	Hungary (2004)<br>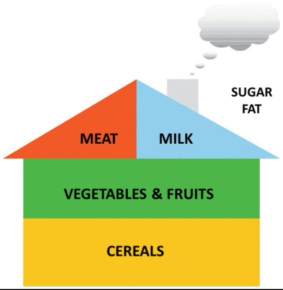	Portugal (2016)<br>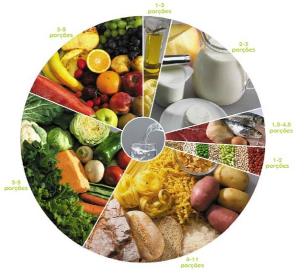	Türkiye (2006)<br>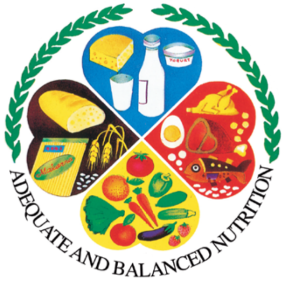
	Romania (2006)<br>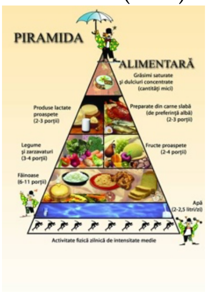	Finland (2014)	Ireland (2015-2016)<br>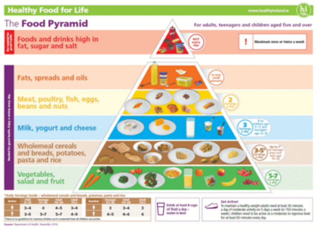	United Kingdom (2016)<br>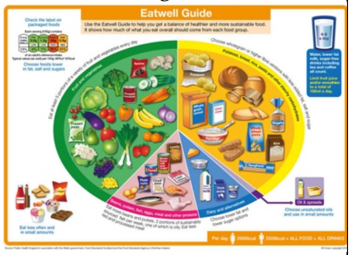

<table>
<tr><td></td><td></td><td>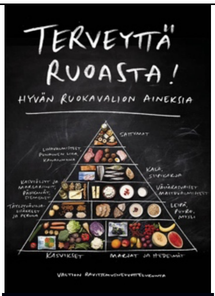

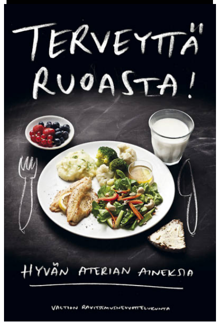
</td><td></td><td></td></tr>
<tr><td></td><td>Belgium (2019)
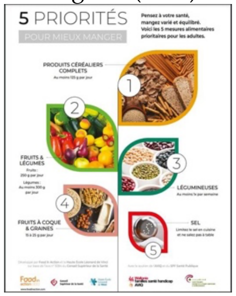

(visual representation for the French population)</td><td>Poland (2020)
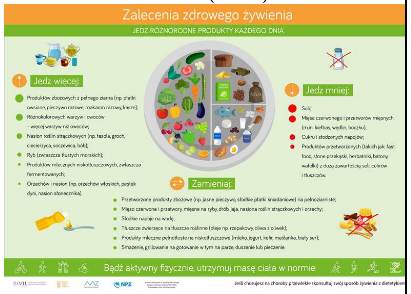
</td><td>Latvia (2020)
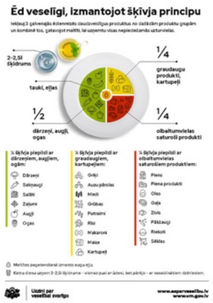
</td><td>Spain (2022)
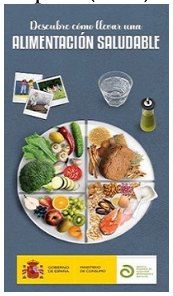
</td></tr>
</table>

<table>
<tr><td rowspan="2"></td><td>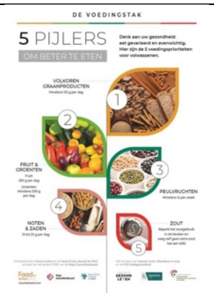

(visual representation for the Flemish population)
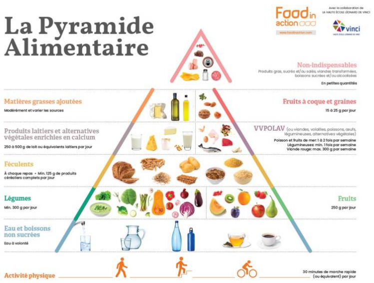

(the food pyramid for the French population)
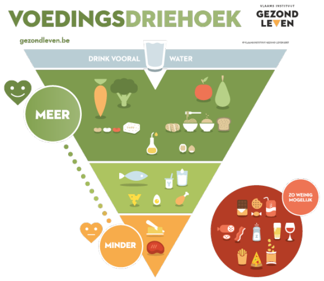

(the food triangle for the Flemish population)</td><td></td><td></td><td></td></tr>
<tr><td>France (2023)</td><td colspan="3"></td></tr>
</table>

<table>
<tr><td></td><td>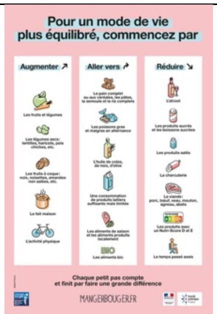
</td><td colspan="3"></td></tr>
<tr><td rowspan="3">WHO Americas Region***
(n=17)</td><td>Dominica (2007)
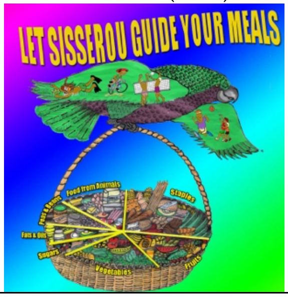
</td><td>St. Kitts and Nevis (2010)
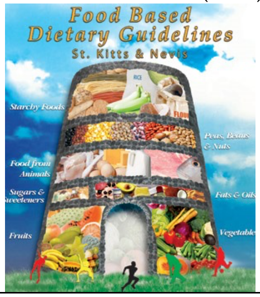
</td><td>Guatemala (2012)
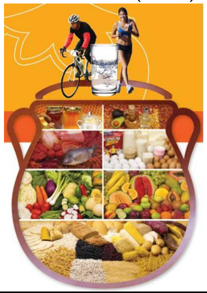</td><td>Honduras (2013)
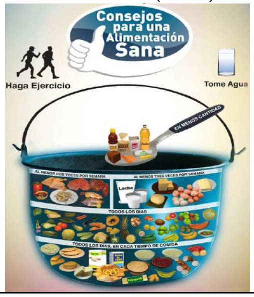
</td></tr>
<tr><td>Bolivia (2014)
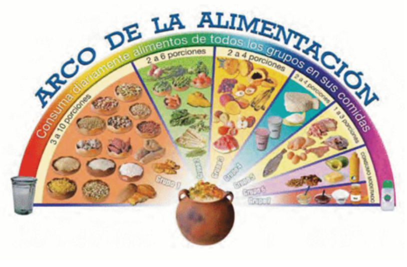
</td><td>Panama (2013)
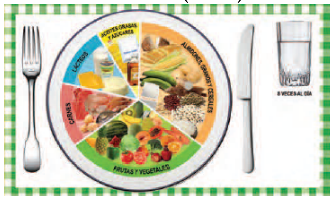</td><td>Barbados (2017)
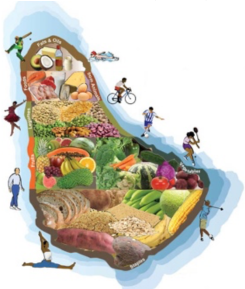</td><td>Bermuda (2017)
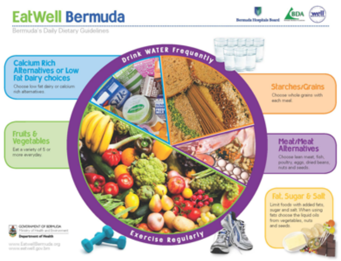
</td></tr>
<tr><td>Canada (2019)</td><td>Peru (2020)</td><td>Colombia (2020)</td><td>Ecuador (2020)
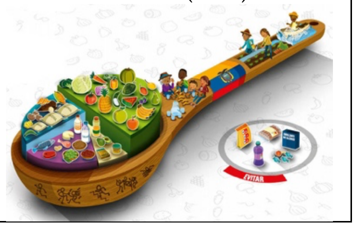</td></tr>
</table>

	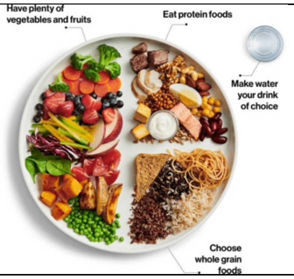	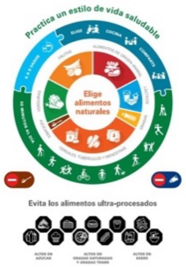	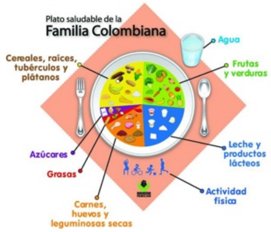	
	Grenada (2020) 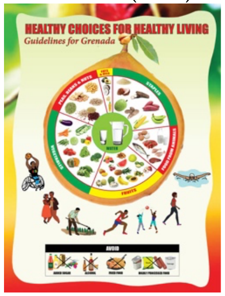	United States (2020) 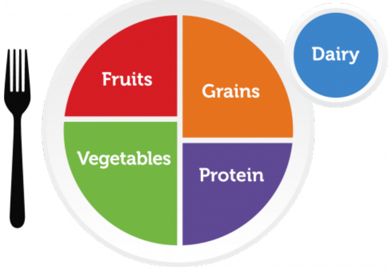	St. Vincent and the Grenadines (2021) 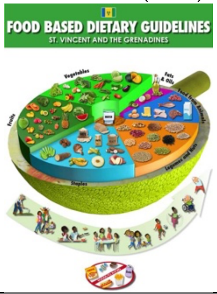	Chile (2022) 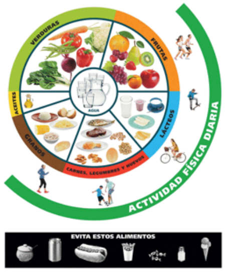
	Mexico (2023) 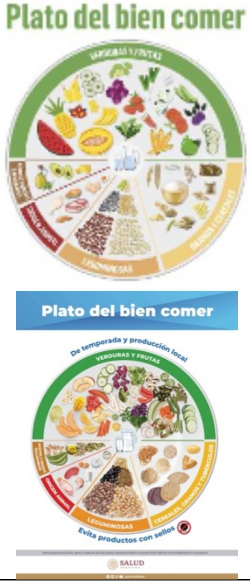			

**WHO Southeast Asian Region**** (*n*=5)**	Thailand (2007) 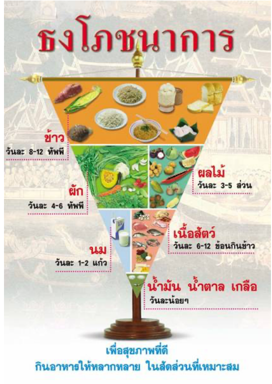	India (2011) 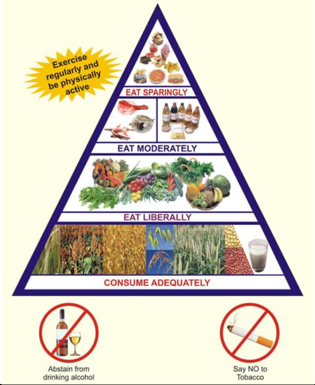	Bangladesh (2013) 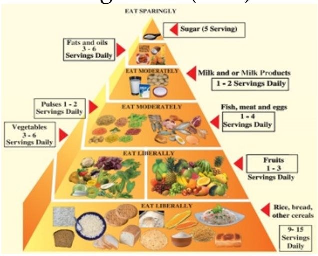	Maldives (2019) 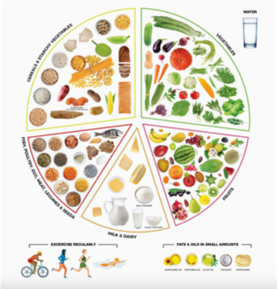
	Sri Lanka (2021) 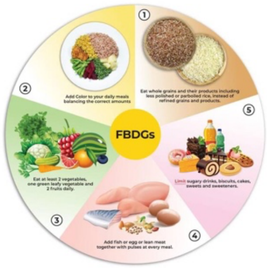			
**WHO Western Pacific Region****** (*n*=5)**	Philippines (2012) 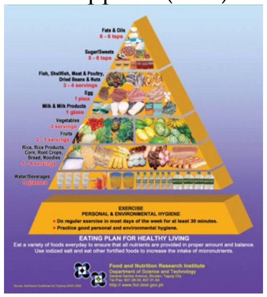 (daily nutritional guide for adults)	Fiji (2013) 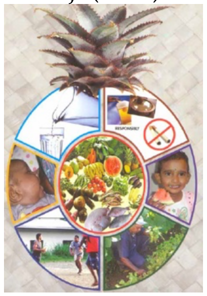	Cambodia (2017) 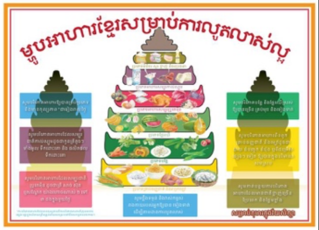	Malaysia (2020) 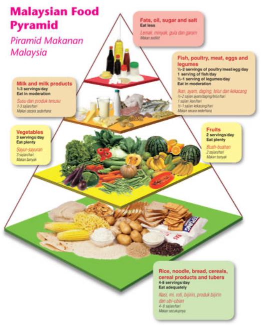

<table>
<tr>
<td rowspan="2">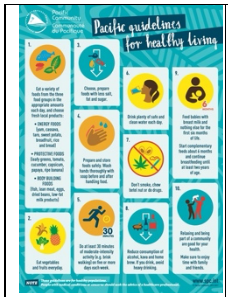
Pacific Guidelines for Healthy Living (2018)</td>
<td>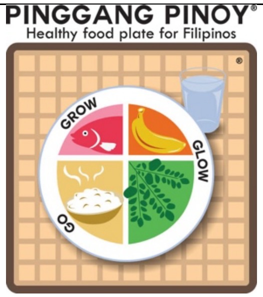
(healthy food plate for adults)</td>
<td></td>
<td></td>
<td>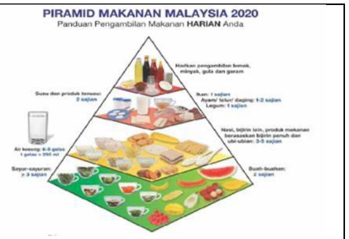
Note: The pyramid within the guidelines is different than the independent image, as it has a glass of water next to the pyramid.</td>
</tr>
<tr>
<td>Brunei (2020)
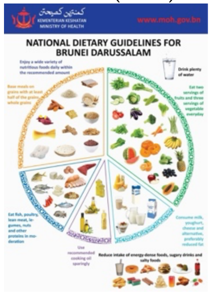</td>
<td colspan="3"></td>
</tr>
</table>

Graphic FBDGs were downloaded from the FAO website [43] or extracted from the technical FBDGs
Graphic FBDGs are not available for the following countries by region:
*WHO Africa Region: Burkina Faso, Cabo Verde, Central African Republic, Côte d'Ivoire, Democratic Republic of the Congo, Equatorial Guinea, Eritrea, Gabon, Mali, Mauritius, Mozambique, Niger, Sao Tome and Principe, and Togo.
**WHO Eastern Mediterranean Region: Bahrain, Morocco, and Tunisia.
***WHO European Region: Azerbaijan, Isle of Man, Monaco, Montenegro, Russian Federation, Tajikistan, and Saint Helena.
****PAHO/WHO Americas Region: El Salvador.
****WHO Southeast Asia Region: Nepal and Democratic Republic of Timor-Leste.
******WHO Western Pacific Region: Cook Islands, French Polynesia, Kiribati, Marshall Islands, Nauru, New Caledonia, Niue, Northern Mariana Islands, Samoa, Tonga, Tuvalu, Vanuatu, and Wallis and Futuna.

Supplemental Table 4. FBDG Healthy Hydration Recommendations for Countries with Sugary Beverage Tax Legislation in the WHO African Region.

WHO Region Country (Policy Coherence Score)	National Dietary Guidelines/graphic FBDG (year)	FBDG Healthy Hydration Recommendations***
**WHO African Region (*n*=21 countries)**		
Benin* (7)	Benin's Dietary Guideline (2015)	**<u>What:</u>**<br>**Water:** "Drink clean water every day to quench thirst. Increase your water intake when it's hot or you're exercising. Sugar-sweetened beverages are not encouraged." (pg. 2)<br><br>**Sugary Beverages:** "Drink clean water every day to quench thirst. Increase your water intake when it's hot or you're exercising. Sugar-sweetened beverages are not encouraged." (pg. 2)<br><br>**<u>Where:</u>**<br>**Water:** N/A<br><br>**Sugary Beverages:** "Consume soft drinks and other sugary drinks in moderation. These drinks only provide sugar and can promote obesity and diabetes." Key message/guideline. (pg. 1)<br><br>**<u>Why:</u>**<br>**Water:** N/A<br><br>**Sugary Beverages:** "Consume soft drinks and other sugary drinks in moderation. These drinks only provide sugar and can promote obesity and diabetes." (pg. 1)<br><br>**<u>How:</u>**<br>**Water:** "Drink clean water every day to quench thirst. Increase your water intake when it's hot or you're exercising." (pg. 2)<br><br>**Sugary Beverages:** "Consume soft drinks and other sugary drinks in moderation." (pg. 1)<br><br>**<u>Quantity/Frequency:</u>**<br>**Water:** N/A<br>**Sugary Beverages:** N/A

		**Image Content:**<br>Visual representation of **water**
Burkina Faso (0)	N/A	
Cabo Verde (0)	N/A	
Central African Republic (0)	N/A	
Côte d'Ivoire (0)	N/A	
Democratic Republic of the Congo (0)	N/A	
Ethiopia (11)	Ethiopia: Food-Based Dietary Guidelines (2022)	**What:**<br>**Water:** "Drink 8-10 large glasses of clean water daily." (pg. 44)<br><br>**Sugary Beverages:** "Limit intake of sugar, sweets and soft drinks to below 30 grams per day." (pg. 48)<br><br>**Where:**<br>**Water:** "Drink 8-10 large glasses of clean water daily." Key message/guideline. (pg. 44)<br><br>**Sugary Beverages:** "Limit intake of sugar, sweets and soft drinks to below 30 grams per day." Key message/guideline. (pg. 48)<br><br>**Why:**<br>**Water:**<br><br>"Water is very vital for good health." (pg. 33)<br><br>"We need a minimum of 8-10 large glasses of water daily for: helping with digestion; carrying nutrients and oxygen to our cells; preventing constipation; normalizing blood pressure; cushioning joints; protecting organs and tissues." (pg. 44)<br><br>**Sugary Beverages:** "Sugary, sweets, cakes, sugar-sweetened beverages and added sugars only provide energy but no other nutrients. Sweets and sugar increase the risk of overweight/obesity and lead to dental problems if eaten too often. Individuals who take too many sugary soft drinks and sugar-loaded sweets are more likely to become overweight and develop diabetes." (pg. 28)<br><br>**How:**

		**Water:** "Take water instead of soft drinks." (pg. 48)<br><br>**Sugary Beverages:**<br><br>"Limit intake of sugar, sweets and soft drinks to below 30 grams per day." (pg. 48)<br><br>"Do not add sugar or honey to your coffee or tea." (pg. 48)<br><br>**<u>Quantity/Frequency:</u>**<br>**Water:** "Drink 8-10 large glasses of clean water daily." (pg. 44)<br><br>**Sugary Beverages:**<br><br>"Free sugar should be limited to 5% of total intake in children and 10% for adults." (pg. 28)<br><br>"Limit intake of sugar, sweets and soft drinks to below 30 grams per day." (pg. 48)<br><br>**<u>Image Content:</u>**<br>Visual representation of **water**
Equatorial Guinea (0)	N/A	
Eritrea (0)	N/A	
Gabon** (3)	National Dietary Guidelines and Recommendations for Healthy Diets (2021)	**<u>What:</u>**<br>**Water: "**Drink water according to your thirst instead of alcoholic beverages, especially pregnant and breastfeeding women." FAO website<br><br>**Sugary Beverages:** N/A<br><br>**<u>Where:</u>**<br>**Water: "**Drink water according to your thirst instead of alcoholic beverages, especially pregnant and breastfeeding women." Key message/guideline. FAO website<br><br>**Sugary Beverages:** N/A<br><br>**<u>Why:</u>**<br>**Water:** N/A<br>**Sugary Beverages:** N/A

		**<u>How:</u>**<br>**Water: "**Drink water according to your thirst instead of alcoholic beverages, especially pregnant and breastfeeding women." FAO website<br><br>**Sugary Beverages:** N/A<br><br>**<u>Quantity/Frequency:</u>**<br>**Water:** N/A<br>**Sugary Beverages:** N/A<br><br>**<u>Image Content:</u>** N/A
Ghana (11)	Ghana: National Food-Based Dietary Guidelines (2023)	**<u>What:</u>**<br>**Water:** "Drink water regularly" (pg. 34)<br><br>**Sugary Beverages:** "It is recommended to limit drinking of sugar-sweetened beverages." (pg. 34)<br><br>**<u>Where:</u>**<br>**Water:**<br>"Drink water regularly" (pg. 34) Key guideline/message.<br><br>**Sugary Beverages:** A dietary and nutrition goal for Ghanaians is to "limit consumption of unhealthy food, particularly fried foods and sugar-sweetened beverages." Not a key recommendation, but this goal is bolded and in a text box. (pg. 8)<br><br>**<u>Why:</u>**<br>**Water:** "Water is important for human health. It is an essential nutrient needed for almost all the body's functions. Water also hydrate the body and quenches thirst." (pg. 34)<br><br>**Sugary Beverages:** "There is a trend towards increased promotion and consumption of energy drinks, and sugar-sweetened and flavoured beverages. This trend is displacing water as a means of hydration and contributing to excessive calorie consumption, linked with overweight, obesity and diet-related diseases." (pg. 34)<br><br>**<u>How:</u>**<br>**Water:**

		"Drink at least eight cups or glasses of water in a day (equivalent to four sachets). (pg. 34)<br><br>**Sugary Beverages:**<br>"Drink beverages with no or low amount of sugar." (pg. 34)<br><br>**<u>Quantity/Frequency:</u>**<br>**Water:** "Drink at least eight cups or glasses of water in a day (equivalent to four sachets)." (pg. 34)<br><br>**Sugary Beverages:** "Daily amount of table sugar added to drinks, and other foods (sweet snacks, cereals, poridges) should not exceed four table spoons (about 50 grams) for a person each day." (pg. 26)<br><br>**<u>Image Content:</u>**<br>Visual representation of **water**
Mali (0)	N/A	
Mauritius (0)	N/A	
Mozambique (0)	N/A	
Niger (0)	N/A	
Nigeria (5)	Food-Based Dietary Guidelines for Nigeria – A Guide to Healthy Eating (2001)	**<u>What:</u>**<br>**Water:** "Drink water as much as possible daily." (pg. 3)<br><br>**Sugary Beverages:** "Avoid sweetened carbonated (soft) drinks" "sugarcane sweet (Alewa) soft drink" "cocoa-based beverages" "chocolate drinks" and "cocoa-based beverages" Diet Related Diseases; (pg. 23-30)<br><br>**<u>Where:</u>**<br>**Water:** N/A<br>**Sugary beverages:** N/A<br><br>**<u>Why:</u>**<br>**Water:** N/A<br>**Sugary beverages:** N/A<br><br>**<u>How:</u>**<br>**Water:** "Drink water as much as possible daily." (pg. 3)

		**Sugary Beverages:** "Avoid sweetened carbonated (soft) drinks" "sugarcane sweet (Alewa) soft drink" "cocoa-based beverages" "chocolate drinks" and "cocoa-based beverages" Diet Related Diseases; (pg. 23-30)<br><br>**<u>Quantity/Frequency:</u>**<br>**Water:** N/A<br>**Sugary beverages:** N/A<br><br>**<u>Image Content:</u>**<br>Visual representation of **water**
Sao Tome and Principe (0)	N/A	
Seychelles (9)	The Seychelles Dietary Guidelines (2006)	**<u>What:</u>**<br>**Water:** "Drink at least 8 glasses of water every day" (pg. 1)<br><br>**Sugary Beverages:** "Consume sugar, sugary foods and sugary drinks in minimal amounts." (pg. 1)<br><br>**<u>Where:</u>**<br>**Water:** "Drink at least 8 glasses of water every day" (pg. 1) Key guideline/message.<br><br>**Sugary Beverages:** "Consume sugar, sugary foods and sugary drinks in minimal amounts." (pg. 1) Key guideline/message.<br><br>**<u>Why:</u>**<br>**Water:** N/A<br>**Sugary beverages:** N/A<br><br>**<u>How:</u>**<br>**Water:** "Drink at least 8 glasses of water every day" (pg. 1)<br><br>**Sugary Beverages:** "Consume sugar, sugary foods and sugary drinks in minimal amounts." (pg. 1)<br><br>**<u>Quantity/Frequency:</u>**<br>**Water:** "Drink at least 8 glasses of water every day" (pg. 1)<br><br>**Sugary beverages:** N/A

		**Image Content:**<br>Visual representation of **water**<br>Visual representation of **sugary beverages**
South Africa (10)	Food-Based Dietary Guidelines for South Africa (2013)	**What:**<br>**Water:** "Drink lots of clean, safe water" (pg. S77)<br><br>**Sugary Beverages**: "Use foods and drinks containing sugar sparingly and not between meals" (pg. S100)<br><br>**Where:**<br>**Water:** "Drink lots of clean, safe water" (pg. S77) Key guideline/message.<br><br>**Sugary Beverages**: N/A<br><br>**Why:**<br>**Water:** "Water is an essential nutrient and an important multifunctional constituent of the body, with roles as thermo-regulator, building material of cells in the body, a shock absorber, lubricant, solvent and carrier of various compounds, nutrients and waste products. Water balance and hydration status is precisely regulated by an array of sensitive physiological mechanisms which respond to changes in consumption and losses, and this changes in plasma osmolarity." (pg. S77)<br><br>**Sugary Beverages:** "Research studies that have been published over the past decade have provided a solid body of valuable evidence that indicates that a relatively high intake of added sugar, especially SSBs, plays a significant role in obesity and type 2 diabetes, and probably cardiovascular disease too." (pg. S103)<br><br>**How:**<br>**Water:** "Drink lots of clean, safe water" (pg. S77)<br><br>**Sugary Beverages**: "Use foods and drinks containing sugar sparingly and not between meals" (pg. S100)<br><br>**Quantity/Frequency:**<br>**Water:** "General recommendation for total daily water intake are between 2 and 3.7 L for women and men, 0.7 L for infants aged 0-6 months, 0.8 L for infants aged 7-12 months, 1.3 for children aged 1-3 years, and 1.7 l for children aged 4-8 years." (pg. S77)<br><br>**Sugary Beverages:**

		"We propose that an intake of added sugar of 10% of dietary energy is an acceptable upper limit." "This translates to a maximum intake of one serving (approximately 355 ml) of SSBs per day, if no other foods with added sugar are eaten." (pg. S100)<br><br>"A 335-ml tin of an SSB (one serving) contains approximately 40 g of sugar (150 calories, 630 kJ.) Drinking one tin per day translates to approximately 6-7% of energy." (pg. S103)<br><br>"Both adults and children should limit the consumption of SSBs to one tin per day, or the equivalent amount of added sugar from other foods." (pg. S103)<br><br>**Image Content:**<br>Visual representation of **water**
Togo (0)	N/A	
Zambia (8)	Zambia Food-Based Dietary Guidelines Technical Recommendations (2021)	**What:**<br>**Water:** "Choose water as the main drink instead of sugar-sweetened beverages like soda, juices and squashes." (pg. 95)<br><br>**Sugary Beverages:** "Choose water as the main drink instead of sugar-sweetened beverages like soda, juices and squashes." (pg. 95)<br><br>**Where:**<br>**Water:** "Choose water as the main drink instead of sugar-sweetened beverages like soda, juices and squashes." Highlighted in helpful tips text box. (pg. 95)<br><br>**Sugary Beverages:** "Choose water as the main drink instead of sugar-sweetened beverages like soda, juices and squashes." Highlighted in helpful tips text box (pg. 95)<br><br>**Why:**<br>**Water:** N/A<br><br>**Sugary Beverages:** "Free sugars – particularly in the form of sugar-sweetened beverages – increase the overall energy intake and may reduce the intake of foods containing more nutritionally adequate calories and nutrients, leading to micronutrient deficiencies." (pg. 94)<br><br>**How:**

		**Water:**<br>"Choose water as the main drink instead of sugar-sweetened beverages like soda, juices and squashes." (pg. 95)<br><br>**Sugary Beverages:**<br>"Choose water as the main drink instead of sugar-sweetened beverages like soda, juices and squashes." (pg. 95)<br><br>"Limit consumption of sugar-sweetened foods such as pastries, sodas and beverages, soft drinks and fruit flavored drinks, and sweetened yogurts." (pg. 95)<br><br>**<u>Quantity/Frequency:</u>**<br>**Water:** N/A<br><br>**Sugary Beverages:** "limit or avoid adding sugars to foods and drinks like tea, or taking foods and drinks high in added sugars. The WHO suggests further reducing the intake of sugar to less than 5 percent of total energy (25 g or 6 tablespoons) to protect dental health and for additional health benefits throughout life." (pg. 94)<br><br>**<u>Image Content:</u>**<br>No visual representation of water or sugary beverages

*FBDG technical document searched using Google translation of keywords: "water," "hydration," "sugar-sweetened beverage," "sugary beverages," "beverages" and "drink" and/or translated key messages and guidelines from the FAO website were reviewed.
**FBDG technical document did not allow for keyword searches or was unavailable. Translated key messages and guidelines extracted from FAO website.
*****These were selected examples from FBDGs and are not comprehensive of every relevant recommendation**

Supplemental Table 5. FBDG Healthy Hydration Recommendations for Countries with Sugary Beverage Tax Legislation in the WHO Eastern Mediterranean Region.

WHO Region Country (Policy Coherence Score)	National Dietary Guidelines/graphic FBDG (year)	FBDG Healthy Hydration Recommendations***
**WHO Eastern Mediterranean Region (*n*=8 countries)**		
Bahrain (4)	Naja et al. 2023, The Bahrani food-based dietary guidelines: a	**<u>What:</u>**<br>**Water:** "Stay hydrated with water and healthy fluids" (pg. 3)<br><br>**Sugary Beverages:** N/A

	holistic perspective to health and wellbeing	**<u>Where:</u>**<br>**Water:** "Stay hydrated with water and healthy fluids" (pg. 3) Key message/guideline.<br><br>**Sugary Beverages:** N/A<br><br>**<u>Why:</u>**<br>**Water:** "Drinking enough water is essential for physical health, as it is involved in most body functions" (pg. 4)<br><br>**Sugary Beverages:** N/A<br><br>**<u>How:</u>**<br>**Water:** "Stay hydrated with water and healthy fluids" (pg. 3)<br><br>**Sugary Beverages:** N/A<br><br>**<u>Quantity/Frequency:</u>**<br>**Water:** N/A<br>**Sugary Beverages:** N/A<br><br>**<u>Image Content:</u>**<br>N/A
Morocco (0)	N/A	
Oman (5)	The Omani Guide to Healthy Eating (2009)	**<u>What:</u>**<br>**Water:** "Be active, exercise regularly and drink plenty of water." (pg. 40)<br><br>**Sugary Beverages:** N/A<br><br>**<u>Where:</u>**<br>**Water:** "Be active, exercise regularly and drink plenty of water." (pg. 40) Key guideline/message.<br><br>**Sugary Beverages:** N/A<br><br>**<u>Why:</u>**<br>**Water:** N/A<br>**Sugary Beverages:** N/A

		**How:**<br>**Water:** "Be active, exercise regularly and drink plenty of water." (pg. 40)<br><br>**Sugary Beverages:** N/A<br><br>**Quantity/Frequency:**<br>**Water:** N/A<br><br>**Sugary Beverages:** "Free sugars should not exceed 10% of the total calories in the diet." (pg. 18)<br><br>**Image Content:**<br>Visual representation of **water**
Pakistan (11)	Pakistan Dietary Guidelines for Better Nutrition (2018)	**What:**<br>**Water:**<br>"Drink plenty of water each day" (pg. 56)<br><br>**Sugary Beverages:**<br>"Reduce sugar intake, and limit intake of soft drinks, confectionaries, bakery products and commercial fruit drinks" (pg. 54)<br><br>**Where:**<br>**Water:** "Drink plenty of water" (pg. 56) Key Message/guideline.<br><br>**Sugary Beverages:** "Reduce sugar intake, and limit intake of soft drinks, confectionaries, bakery products and commercial fruit drinks" (pg. 54) Key Message/guideline.<br><br>**Why:**<br>**Water:** "Drinking at least 8 glasses of clean and safe water per day if recommended for proper body functions." (pg. 56)<br><br>**Sugary Beverages:** "Soft drinks, confectionaries, bakery products and commercial fruit drinks are energy dense foods and mostly provide empty calories increasing the risk of overweight and obesity. These foods also contain large amount of sugar. Consumption of soft drinks and commercial sweet carbonated beverages has been strongly associated with increased weight gain, diabetes and pancreatic cancer." (pg. 54-55)<br><br>**How:**

		**Water:** "Drink plenty of water each day" (pg. 56)<br><br>**Sugary Beverages:** "To fight the epidemic of obesity, consumers are constantly reminded to reach out for fresh fruits and vegetables while avoiding sugary drinks and fried items" (pg. 20)<br><br>**<u>Quantity/Frequency:</u>**<br>**Water:**<br><br>"Drinking at least 8 glasses of clean and safe water per day is recommended for proper body functions." (pg. 56)<br><br>"For a reference person of 65 kg, minimum required amount of drinking water is 2 liters (8 glasses) including all fluids." (pg. 56)<br><br>**Sugary Beverages:** "Less than 10% calories from free sugar." (pg. 37)<br><br>**<u>Image Content:</u>**<br>Visual representation of **water**
Qatar (10)	Qatar Dietary Guidelines (2015)	**<u>What:</u>**<br>**Water:** "Drink plenty of water" (pg. 29)<br><br>**Sugary Beverages:** "Avoid sweetened beverages such as carbonated, energy and fruit drinks." (pg. 22)<br><br>**<u>Where:</u>**<br>**Water:** "Drink plenty of water" (pg. 29) Key message/guideline.<br><br>**Sugary Beverages:** "Avoid sweetened beverages such as carbonated, energy and fruit drinks." (pg. 22) Key message/guideline in text box.<br><br>**<u>Why:</u>**<br>**Water:** "Water is essential for life, as it is involved in many functions in the body" (pg. 29)<br><br>**Sugary Beverages:**<br><br>"Sugar sweetened drinks such as soda, energy drinks, fruit drinks, vitamin waters and sports drinks are the largest sources of added sugar for many people. Drinking a lot of these can cause weight gain, and is also related to increased dental cavities and reduced bone strength" (pg. 22)

		"Consuming sugar sweetened beverages is also associated with weight gain." (pg. 29)<br><br>**<u>How:</u>**<br>**Water:**<br>"When eating out, order sparkling water with a twist of lemon, laban, milk, fresh juice or water instead of soda." (pg. 22)<br><br>"Choose water as a drink with meals; Choose water instead of sugar-sweetened beverages to help maintain your weight and the health of your teeth; Add a wedge of lime or lemon to enhance the taste of water." (pg. 29)<br><br>"Drink more water in hot weather and when active." (pg. 29)<br><br>**Sugary Beverages:**<br><br>"Avoid sweetened drinks such as soda, energy drinks, fruit drinks, vitamin waters and sports drinks." (pg. 22)<br><br>**<u>Quantity/Frequency:</u>**<br>**Water:**<br><br>"Most health recommendations suggest about 8 cups water per day." (pg. 29)<br><br>"Drink 2- 3 litres (8-12 cups) of fluid each day, choosing water often." (pg. 29)<br><br>**Sugary Beverages:** N/A<br><br>**<u>Image Content:</u>**<br>Visual representation of **water**
Saudi Arabia (8)	Dietary Guidelines for Saudis: The healthy Food Palm (2012)	**<u>What:</u>**<br>**Water:** "Drink water" (pg. 26)<br><br>**Sugary Beverages:** "The Dietary Guidelines also aiming to make use of the food items enriched with nutrients important to the body such as proteins, vitamins, minerals, and fibers according to the recommended dietary allowance, and to avoid unhealthy food items such as fast food, soft drinks, food items rich with salt, sugar and saturated fat and modification of dietary habits." (pg. 3)<br><br>**<u>Where:</u>**<br>**Water:** "Drink water" (pg. 26) Key message/guideline.

		**Sugary Beverages:** N/A<br><br>**<u>Why:</u>**<br>**Water:**<br>"Water has numerous roles in the human body. It acts as a building material; as solvent, as a carrier for nutrients and waste products and in thermoregulation. Both water intake and water losses are controlled to reach water balance." (pg. 19)<br><br>"Water is an important fluid to eliminate waste and metabolism residues from the body; Improve the health of kidneys; Best way to quench your thirst and it is calorie free; To balance level of minerals and vitamins in the body." (pg. 26)<br><br>**Sugary Beverages:** N/A<br><br>**<u>How:</u>**<br>**Water:** "Drink water" (pg. 26)<br><br>**Sugary Beverages:** "The Dietary Guidelines also aiming to make use of the food items enriched with nutrients important to the body such as proteins, vitamins, minerals, and fibers according to the recommended dietary allowance, and to avoid unhealthy food items such as fast food, soft drinks, food items rich with salt, sugar and saturated fat and modification of dietary habits." (pg. 3)<br><br>**<u>Quantity/Frequency:</u>**<br>**Water:**<br><br>"Drinking water per day, at least 6 cups (240 ml)" (pg. 4)<br><br>"On an average, a sedentary adult should drink at least 1.5 litres per day." (pg. 19)<br><br>"It is important to drink (1.5 liter) daily which is approximately equal to (6 cups)" (pg. 26)<br><br>**Sugary Beverages:** N/A<br><br>**<u>Image Content:</u>**<br>Visual representation of **water**
Tunisia (0)	N/A	

United Arab Emirates (4)	United Arab Emirates Dietary Guidelines (2019)	**<u>What:</u>**<br>**Water:** "Drink adequate amount of fluid (including water, tea, clear soup, etc) every day" (pg. 2)<br><br>**Sugary Beverages:** N/A<br><br>**<u>Where:</u>**<br>**Water:** "Drink adequate amount of fluid (including water, tea, clear soup, etc) every day" (pg. 2) Key message/guideline.<br><br>**Sugary Beverages:** N/A<br><br>**<u>Why:</u>**<br>**Water:** N/A<br><br>**Sugary Beverages:** N/A<br><br>**<u>How:</u>**<br>**Water:** "Drink adequate amount of fluid (including water, tea, clear soup, etc) every day" (pg. 2)<br><br>**Sugary Beverages:** N/A<br><br>**<u>Quantity/Frequency:</u>**<br>**Water:** N/A<br>**Sugary Beverages:** N/A<br><br>**<u>Image Content:</u>**<br>Visual representation of **water**

*****These were selected examples from FBDGs and are not comprehensive of every relevant recommendation**

Supplemental Table 6. FBDG Healthy Hydration Recommendations for Countries with Sugary Beverage Tax Legislation in the WHO European Region.

**WHO Region Country (Policy Coherence Score)**	**National Dietary Guidelines/graphic FBDG (year)**	**FBDG Healthy Hydration Recommendations*****
**WHO European Region (*n*=20 countries)**		

Azerbaijan (0)	N/A	
Belgium (11)	Dietary Guidelines for Belgian Adult Population (2019)	<u>**What:**</u><br>**Water:** "Consume as few drinks with added sugars as possible and choose water instead." (pg. 63)<br><br>**Sugary Beverages:** "Consume as few drinks with added sugars as possible and choose water instead." (pg. 63)<br><br><u>**Where:**</u><br>**Water:** N/A<br><br>**Sugary Beverages:** Beverages and foods containing added sugars recommendations are highlighted in a summary box. (pg. 65)<br><br>"Drink as few beverages with added sugars as possible." (pg. 65) Key message/guideline.<br><br><u>**Why:**</u><br>**Water:**<br><br>"An adequate water intake is crucial for various functions of the body and must be in balance with fluid loss." (pg. 15)<br><br>"Our body is 60% water, and its water reserves needs to be replenished on a regular basis." (pg. 63)<br><br>**Sugary Beverages:** "Excess intake of certain types of sugar (mainly sucrose, glucose, and fructose) has been associated with tooth decay in children, especially in the event of poor dental hygiene. An association could be made between added sugar, especially fructose, and the development of non-alcoholic fatty liver disease, which can then progress to more severe liver diseases. Added sugar could also have an effect on the appearance of overweight, though no consensus has been reached to date on this subject. Yet it is generally acknowledged that added sugar in soft drinks has a negative impact on cardiometabolic risk factors." (pg. 15)<br><br><u>**How:**</u><br>**Water:**<br><br>"Consume as few drinks with added sugars as possible and choose water instead." (pg. 63)<br><br>"Choose drinks with no added sugars, water being the first choice." (pg. 65)<br><br>**Sugary Beverages:**

		"Consume as few drinks with added sugars as possible and choose water instead." (pg. 63)<br><br>"Choose drinks with no added sugars, water being the first choice." (pg. 65)<br><br>**<u>Quantity/Frequency:</u>**<br>**Water:** "To maintain a healthy water balance, adults and young people should consume 1 to 1.5 litres of water daily." (pg. 63)<br><br>**Sugary Beverages:** "Avoid sugary drinks with an energy content over 50 kcal per 227 ml serving or 22 kcal/100 ml, or that contain more than 5% sugar." (pg. 63)<br><br>Theoretical minimum-risk exposure levels for sugar-sweetened beverages is 0-5 g/day. (pg. 24)<br><br>**<u>Image Content:</u>**<br>Visual representation of **water**<br>Visual representation of **sugary beverages**
Croatia** (0)	Dietary Guidelines for Adults (2002)	**<u>What:</u>**<br>**Water:** N/A<br>**Sugary Beverages:** N/A<br><br>**<u>Where:</u>**<br>**Water:** N/A<br>**Sugary Beverages:** N/A<br><br>**<u>Why:</u>**<br>**Water:** N/A<br>**Sugary Beverages:** N/A<br><br>**<u>How:</u>**<br>**Water:** N/A<br>**Sugary Beverages:** N/A<br><br>**<u>Quantity/Frequency:</u>**<br>**Water:** N/A<br>**Sugary Beverages:** N/A

		**<u>Image Content:</u>**<br>No visual representation of water or sugary beverages
Finland (9)	Nordic Nutrition Recommendations (2012)	**<u>What:</u>**<br>**Water:** "Safe water for drinking and sanitation is critical to maintaining good health." (pg. 155)<br><br>**Sugary Beverages:** "Limit sugar-sweetened beverages." (pg. 123)<br><br>**<u>Where:</u>**<br>**Water:** Fluid and water balance is a distinct section of guidelines. (pg. 155)<br><br>**Sugary Beverages:** "Limit beverages and foods with added sugar" (pg. 124) Outlined in table to promote energy balances and health in Nordic populations.<br><br>**<u>Why:</u>**<br>**Water:**<br>"Safe water for drinking and sanitation is critical to maintaining good health." (pg. 155)<br><br>"Water is the main component of the human body and is vital for organ functions and for thermoregulation." (pg. 156)<br><br>**Sugary Beverages:**<br>"A limited consumption of SSB will contribute to an increased micronutrient density and a reduced intake of added sugars." (pg. 123)<br><br>"Consumption of sugar-sweetened drinks has been associated with an increased risk of type-2 diabetes and excess weight-gain and should, therefore, be limited" (pg. 272)<br><br>**<u>How:</u>**<br>**Water:** N/A<br><br>**Sugary Beverages:** "Limit sugar-sweetened beverages." (pg. 123)<br><br>**<u>Quantity/Frequency:</u>**<br>**Water:** "In NNR 2012 the guiding value for daily intake of drinking fluids for adults and children performing moderate physical activity and living under moderate temperate conditions is 1–1.5 litres of water in addition to the water derived from foods." (pg. 158)

		**Sugary Beverages:** "Added sugars (sucrose, fructose, and starch hydrolysates) should be kept below 10 E%." (pg. 272)<br><br>**Image Content:**<br>No visual representation of water or sugary beverages
France* (9)	50 Tips for Eating Better and Moving More (2023)	**What:**<br>**Water:** "Tap water: the right pipe. Consuming tap water means reducing waste and expenses." (pg. 29)<br><br>**Sugary Beverages:** "Reduce sugary drinks." (pg. 18)<br><br>**Where:**<br>**Water:** "Tap water: the right pipe." (pg. 29) Key message/guideline; Bolded and numbered.<br><br>**Sugary Beverages:** "Reduce sugary drinks." (pg. 18, pg. 32) Bolded and includes images.<br><br>**Why:**<br>**Water:** N/A<br><br>**Sugary Beverages:** "A quarter of the foods consumed during the day by adults in France is consumed between meals, as a snack or as an aperitif. These are often sweet or savory products but also sugary drinks, foods and alcohol, which promotes weight gain." (pg. 19)<br><br>**How:**<br>**Water:**<br><br>"Hydrate well to start the day well." (pg. 6)<br><br>"Remember that water is the only recommended drink." (pg. 8)<br><br>**Sugary Beverages:** "Reduce sugary drinks." (pg. 18)<br><br>**Quantity/Frequency:**<br>**Water:** N/A<br><br>**Sugary Beverages:** "All fruit juices, sugary drinks and sodas, even diet, and so-called "energy" drinks should be limited as much as possible. In any case, no more than one drink per day." (pg. 18)

		**Image Content:**<br>Visual representation of **sugary beverages**
Hungary* (7)	Dietary Guidelines for the Adult Population in Hungary (2004)	**What:**<br>**Water:** “To quench your thirst, drink water or mineral water instead of sugary drinks.” (pg. 16)<br><br>**Sugary Beverages:** “Excessive sugar intake is a risk factor for certain diseases. Consume sugars, sugar-rich sweets and drinks in moderation.” (pg. 16)<br><br>**Where:**<br>**Water:** N/A<br><br>**Sugary Beverages:**<br><br>“Reduce the consumption of foods and drinks rich in sugar.” (pg. 4) Key message/guideline.<br><br>“Excessive sugar intake is a risk factor for certain diseases. Consume sugars, sugar-rich sweets and drinks in moderation.” (pg. 16) Key message/guideline and in text box.<br><br>**Why:**<br>**Water:** N/A<br><br>**Sugary Beverages:** “Excessive sugar intake is a risk factor for certain diseases. Consume sugars, sugar-rich sweets and drinks in moderation.” (pg. 16)<br><br>**How:**<br>**Water:** “To quench your thirst, drink water or mineral water instead of sugary drinks.” (pg. 16)<br><br>**Sugary Beverages:** “Consume sugars, sugar-rich sweets and drinks in moderation.” (pg. 16)<br><br>**Quantity/Frequency:**<br>**Water:** “Drink 6–8 glasses of water or mineral water a day.” (pg. 25)<br><br>**Sugary Beverages:** N/A<br><br>**Image Content:**<br>No visual representation of water or sugary beverages

Ireland (9)	Healthy Food for Life – the Healthy Eating Guidelines (2015-2016)	**What:**<br>**Water:**<br><br>"Drink more water – at least 8 glasses or cups a day" (pg. 7)<br><br>"Drink at least 8 cups of fluid a day – water is best" (pg. 4)<br><br>**Sugary Beverages:**<br><br>"Don't eat the following foods and drinks every day: Sugary drinks" (pg. 2)<br><br>"Limit foods and drinks high in fat, sugar and salt to sometimes and only in small amounts; Not every day, maximum once or twice a week." (pg. 2)<br><br>**Where:**<br>**Water:** N/A<br><br>**Sugary Beverages:** Sugary drinks listed as something to avoid on table (pg. 2)<br><br>**Why:**<br>**Water:** N/A<br><br>**Sugary Beverages:**<br><br>"They are not needed for health and may promote overweight and obesity." – referring to foods and drinks high in fat, sugar, and salt (pg. 4)<br><br>"Food and drinks high in fat, sugar and salt. This is the smallest shelf and is at the top of the Food Pyramid so people need to choose very little of these – not every day, maximum once of twice a week only. They are needed for health and may promote overweight and obesity." (pg. 4)<br><br>**How:**<br>**Water:**<br><br>"Drink more water – at least 8 glasses or cups a day" (pg. 7)<br><br>"Drink at least 8 cups of fluid a day – water is best" (pg. 4)

		**Sugary Beverages:**<br><br>"Limit foods and drinks high in fat, sugar and salt to sometimes and only in small amounts." (pg. 2)<br><br>"Limit foods and drinks high in fat, sugar and salt to sometimes and only in small amounts; Not every day, maximum once or twice a week." (pg. 2)<br><br>**<u>Quantity/Frequency:</u>**<br>**Water:**<br><br>"Drink more water – at least 8 glasses or cups a day" (pg. 7)<br><br>"Drink at least 8 cups of fluid a day – water is best" (pg. 4)<br><br>**Sugary Beverages:** N/A<br><br>**<u>Image Content:</u>**<br>Visual representation of **water**<br>Visual representation of **sugary beverages**
Isle of Man (0)	N/A	
Latvia* (8)	Dietary Guidelines/Healthy Eating Recommendations for Adults (2020)	**<u>What:</u>**<br>**Water:** "Drink 1.5-2 litres of liquid, including water, every day" FAO website<br><br>**Sugary Beverages:**<br><br>"It is not recommended to choose sweetened drinks (including lemonade), juices for thirst quenching drinks or juice-based sweetened drinks and other similar drinks because they contain a lot added sugar and energy. For example, in a 300 ml sweetened soft drink (incl. in lemonades) can contain up to 40 g of sugar (8 teaspoons), which corresponds to 160 kcal, and exceeds the WHO's recommended daily intake of sugar." (pg. 4)<br><br>**<u>Where:</u>**<br>**Water:** "Drink 1.5-2 litres of liquid, including water, every day" Listed as a key message/guideline on FAO website<br><br>**Sugary Beverages:** N/A

		**Why:**<br>**Water:** N/A<br><br>**Sugary Beverages:** “It is not recommended to choose sweetened drinks (including lemonade), juices for thirst quenching drinks or juice-based sweetened drinks and other similar drinks because they contain a lot added sugar and energy. For example, in a 300 ml sweetened soft drink (incl. in lemonades) can contain up to 40 g of sugar (8 teaspoons), which corresponds to 160 kcal, and exceeds the WHO's recommended daily intake of sugar.” (pg. 4)<br><br>**How:**<br>**Water:** “Drink 1.5-2 litres of liquid, including water, every day” FAO website<br><br>**Sugary Beverages:** N/A<br><br>**Quantity/Frequency:**<br>**Water:** “Drink 1.5-2 litres of liquid, including water, every day” FAO website<br><br>**Sugary Beverages:** “In order to promote and improve health, it is recommended not to take more sugar in your daily diet by 5% of the daily energy intake, or on average up to 25 g per day. World Health Organization it is strictly not recommended to exceed 50 g of sugar per day. The recommended amount of sugar includes the sugar we use at home in tea, coffee or cooking, as well as the sugar we ingest along with ready-to-eat foods such as cookies, sweetened yogurts, breakfast flakes.” (pg. 10)<br><br>**Image Content:**<br>Visual representation of **water**
Monaco (0)	N/A	
Montenegro (0)	N/A	
Poland* (7)	Healthy Eating Recommendations: Plate of Healthy Eating (2020)	**What:**<br>**Water:** “Replace sweetened beverages with water” FAO website<br><br>**Sugary Beverages:** “Eat less sugar and sweetened beverages” FAO website<br><br>**Where:**<br>**Water:** “Replace sweetened beverages with water” FAO website. Key message/guideline.<br><br>**Sugary Beverages:** “Eat less sugar and sweetened beverages” FAO website. Key message/guideline.

		**Why:**<br>**Water:** N/A<br>**Sugary Beverages:** N/A<br><br>**How:**<br>**Water:** "Replace sweetened beverages with water" FAO website<br><br>**Sugary Beverages:** "Eat less sugar and sweetened beverages" FAO website<br><br>**Quantity/Frequency:**<br>**Water:** N/A<br>**Sugary Beverages:** N/A<br><br>**Image Content:**<br>Visual representation of **water**
Portugal* (9)	Food Wheel Guide (2016)	**What:**<br>**Water:**<br>"Although water is the best drink to quench your thirst, you can also use other drinks that do not contain added sugar, alcohol or caffeine." (pg. 3)<br><br>"Prefer Water to Beverages Containing Added Sugar, Alcohol, and Caffeine" FAO website<br><br>**Sugary Beverages:**<br>"Although water is the best drink to quench your thirst, you can also use other drinks that do not contain added sugar, alcohol or caffeine." (pg. 3)<br><br>"Prefer Water to Beverages Containing Added Sugar, Alcohol, and Caffeine" FAO website<br><br>**Where:**<br>**Water:**<br>"Eat foods from each group and drink water daily." (pg. 5) Key message/guideline.<br><br>"Prefer Water to Beverages Containing Added Sugar, Alcohol, and Caffeine" FAO website. Key message/guideline.

		**Sugary Beverages:**<br>"Prefer Water to Beverages Containing Added Sugar, Alcohol, and Caffeine" FAO website. Key message/guideline.<br><br>**<u>Why:</u>**<br>**Water:**<br>"As water is essential to life, it is essential that drink plenty daily." (pg. 4)<br><br>**Sugary Beverages:** N/A<br><br>**<u>How:</u>**<br>**Water:**<br>"Eat foods from each group and drink water daily." (pg. 5)<br><br>**Sugary Beverages:**<br><br>"Prefer Water to Beverages Containing Added Sugar, Alcohol, and Caffeine" FAO website<br><br>**<u>Quantity/Frequency:</u>**<br>**Water:**<br>"Water needs can vary between 1.5 and 3 liters per day." (pg. 4)<br><br>**Sugary Beverages:** N/A<br><br>**<u>Image Content:</u>**<br>Visual representation of **water**
Romania* (5)	Guidelines for a Healthy Diet (2006)	**<u>What:</u>**<br>**Water:** "Water is essential for survival" (pg. 61)<br><br>**Sugary Beverages:** N/A<br><br>**<u>Where:</u>**<br>**Water:** Water recommendations are a chapter dedicated to them (Chapter 4, pg. 61).<br><br>**Sugary Beverages:** N/A<br><br>**<u>Why:</u>**

		**Water:**<br><br>"Water is essential for survival. The human body contains a large amount of water, which is in a continuous movement between the intra and extra cellular pathways, ensuring the development of the processes necessary for survival." (pg. 61)<br><br>Function of water listed on pg. 62 – solvent, transport, body shape, body temperature, and lubricant.<br><br>**Sugary Beverages:** N/A<br><br>**<u>How:</u>**<br>**Water:** N/A<br>**Sugary Beverages:** N/A<br><br>**<u>Quantity/Frequency:</u>**<br>**Water:**<br>"Water from liquids: the daily consumption of water and other liquids is 1200-1500 ml." (pg. 63)<br><br>"In general, an intake of 1 ml of water per kcal for adults and 1.5 ml per kcal for children is recommended." (pg. 64)<br><br>**Sugary Beverages:** N/A<br><br>**<u>Image Content:</u>**<br>Visual representation of **water**
Russian Federation (0)	N/A	
Spain (6)	Healthy and Sustainable Dietary Recommendations Supplemented with Physical Activity Recommendations for the Spanish Population (2022)	**<u>What:</u>**<br>**Water:**<br>"Tap water always" (pg. 2)<br><br>"Water is the drink of choice in a healthy diet." (pg. 11)<br><br>**Sugary Beverages:**<br>"minimise or avoid the consumption of sugary and sweetened beverages" (pg. 12)

		**Where:**<br>**Water:** "Water always." (pg. 11) Key Message/guideline.<br><br>**Sugary Beverages:** N/A<br><br>**Why:**<br>**Water:** N/A<br>**Sugary Beverages:** N/A<br><br>**How:**<br>**Water:**<br>"Drink water whenever you are thirsty." (pg. 11)<br><br>"Always drink tap or running water. The environment impact of bottled water is much higher than that of tap water." (pg. 11)<br><br>**Sugary Beverages:** "minimise or avoid the consumption of sugary and sweetened beverages" (pg. 12)<br><br>**Quantity/Frequency:**<br>**Water:** N/A<br>**Sugary Beverages:** N/A<br><br>**Image Content:**<br>Visual representation of **water**
Saint Helena (0)	N/A	
Tajikistan (0)	N/A	
Türkiye (10)	Dietary Guidelines for Turkey (2006)	**What:**<br>**Water:** "Drink much water and sugar free herbal teas." (pg. 38)<br><br>**Sugary Beverages:** "Instead of drinking beverages containing sugar, water should be preferred." (pg. 43)<br><br>**Where:**<br>**Water:** "Water or other beverages are important in the maintaining of body water balance. It must be provided from the clean water resources for the life." Highlighted in text box. (pg. 51)

		**Sugary Beverages:** "Reduce the consumption of sugar beverages and sweets and choose foods containing less sugar." Highlighted in text box. (pg. 41)<br><br>**<u>Why:</u>**<br>**Water:**<br><br>"Water: It is required to digest foods, to transport them into tissues, to excrete harmful metabolites from body and to regulate body temperature. Availability of water in body is vital for life." (pg.14)<br><br>"Functions of water/fluid in the body: digestion of foods we ate and absorption of the nutrients and also their transportation to the cells; creation of necessary biochemical reactions in the cells for life and health; functioning of cells, tissues, organs and systems; transportation and discharge of harmful substances formed during metabolism; providing the regulation of body temperature; providing the lubrication of the joints." (pg. 51)<br><br>**Sugary Beverages:**<br>"Consuming much sugar and sugar added foods are the basic reason for taking excessive energy and causes to increase the body weight. This situation also reduces to consume foods that are rich with respect to nutritive value." (pg. 41)<br><br>"To consume frequently foods and drinks containing sugar causes tooth decays." (pg. 43)<br><br>**<u>How:</u>**<br>**Water:** "Drink much water and sugar free herbal teas." (pg. 38)<br><br>**Sugary Beverages:**<br><br>"Instead of drinking beverages containing sugar, water should be preferred." (pg. 43)<br><br>"Restrict the beverages and foods containing much sugar." (pg. 43)<br><br>**<u>Quantity/Frequency:</u>**<br>**Water:** "1200-1500 mL/day" (pg. 52)<br><br>**Sugary Beverages:** Table describing grams/portion of sugar (pg. 63)<br><br>**<u>Image Content:</u>**<br>No visual representation of water or sugary beverages

United Kingdom (9)	Eatwell Guide (2016)	**<u>What:</u>**<br>**Water:** "Aim to drink 6-8 glasses of fluid every day. Water, lower fat milk and sugar-free drinks including tea and coffee all count." (pg. 6)<br><br>**Sugary Beverages:** "Swap sugary soft drinks for diet, sugar-free or no added sugar varieties to reduce our sugar intake in a simple step." (pg. 6)<br><br>**<u>Where:</u>**<br>**Water:** "Drink 6-8 cups/glasses of fluid a day." Key Message/guideline (pg. 2)<br><br>**Sugary Beverages:** N/A<br><br>**<u>Why:</u>**<br>**Water:** N/A<br><br>**Sugary Beverages:**<br>"Sugary drinks are one of the main contributors to excess sugar consumption amongst children and adults in the UK." (pg. 6)<br><br>"Regularly consuming foods and drinks high in sugar increases your risk of obesity and tooth decay." (pg. 7)<br><br>**<u>How:</u>**<br>**Water:** "Aim to drink 6-8 glasses of fluid everyday. Water, lower fat milk and sugar-free drinks including tea and coffee all count." (pg. 6)<br><br>**Sugary Beverages:**<br><br>"We should aim to swap sugary drinks for water, lower fat milk or sugar-free drinks including tea and coffee." (pg. 8)<br><br>"Swap sugary soft drinks for diet, sugar-free or no added sugar varieties to reduce our sugar intake in a simple step." (pg. 6)<br><br>**<u>Quantity/Frequency:</u>**<br>**Water:** "Aim to drink 6-8 glasses of fluid everyday. Water, lower fat milk and sugar-free drinks including tea and coffee all count." (pg. 6)

		**Sugary Beverages:**<br><br>"Fruit juice and smoothies also count towards your fluid consumption, although they are a source of free sugars and so you should limit consumption to no more than a combined total of 150ml per day." (pg. 6)<br><br>"Ideally, no more than 5% of energy we consume should come from free sugars." (pg. 7)<br><br>**Image Content:**<br>Visual representation of **water**

*FBDG technical document searched using Google translation of keywords: "water," "hydration," "sugar-sweetened beverage," "sugary beverages," "beverages" and "drink" and/or translated key messages and guidelines from the FAO website were reviewed.
**FBDG technical document did not allow for keyword searches or was unavailable. Translated key messages and guidelines extracted from FAO website.
*****These were selected examples from FBDGs and are not comprehensive of every relevant recommendation**

Supplemental Table 7. FBDG Healthy Hydration Recommendations for Countries with Sugary Beverage Tax Legislation in the PAHO/WHO Americas Region.

**WHO Region Country (Policy Coherence Score)**	**National Dietary Guidelines/graphic FBDG (year)**	**FBDG Healthy Hydration Recommendations*** **
**WHO Americas Region (*n*=18)**		
Barbados (7)	Food Based Dietary Guidelines for Barbados (2017)	**What:**<br>**Water:** "Make water your main beverage." (pg. 4)<br><br>**Sugary Beverages:** "Choose food and beverages with less added sugar every day." (pg. 4)<br><br>**Where:**<br>**Water:** N/A<br><br>**Sugary Beverages:** "Choose food and beverages with less added sugar every day." (pg. 4) Key message/guideline.<br><br>**Why:**<br>**Water:** N/A

		**Sugary Beverages:** "Many foods and beverages with added sugar are high in calories and have little nutritional value. These extra calories can contribute to overweight and obesity. High sugar intake can also increase blood pressure and risk of death from cardiovascular disease."(pg. 4)<br><br>**<u>How:</u>**<br>**Water:** "Make water your main beverage." (pg. 4)<br><br>**Sugary Beverages:** "Choose food and beverages with less added sugar every day." (pg. 4)<br><br>**<u>Quantity/Frequency:</u>**<br>**Water:** N/A<br>**Sugary Beverages:** N/A<br><br>**<u>Image Content:</u>**<br>Visual representation of **water**
Bermuda (5)	EatWell Bermuda: Bermuda's Daily Dietary Guidelines (2017)	**<u>What:</u>**<br>**Water:** "Drink WATER frequently"<br><br>**Sugary Beverages:** N/A<br><br>**<u>Where:</u>**<br>**Water:** "Drink WATER frequently" Key message/guideline.<br><br>**Sugary Beverages:** N/A<br><br>**<u>Why:</u>**<br>**Water:** N/A<br>**Sugary Beverages:** N/A<br><br>**<u>How:</u>**<br>**Water:** "Drink WATER frequently"<br><br>**Sugary Beverages:** N/A<br><br>**<u>Quantity/Frequency:</u>**<br>**Water:** 8 glasses included in graphic FBDG

		**Sugary Beverages:** N/A<br><br>**<u>Image Content:</u>**<br>Visual representation of **water**
Bolivia* (12)	Food-Based Dietary Guidelines for the Bolivian Population (2014)	**<u>What:</u>**<br>**Water:** "Drink daily 6-8 glasses of water complementary to the meals." (pg. 47)<br><br>**Sugary Beverages:** "Avoid excessive consumption of sugar, sweets, carbonated drinks, and alcoholic beverages." (pg. 47)<br><br>**<u>Where:</u>**<br>**Water:** "Drink daily 6-8 glasses of water complementary to the meals." (pg. 47, 56) Key message/guideline.<br><br>**Sugary Beverages:** Avoid excessive consumption of sugar, sweets, carbonated drinks, and alcoholic beverages." (pg. 47) Key message/guideline.<br><br>**<u>Why:</u>**<br>**Water:** "Water is vital for the body. It is not possible to live for a prolonged period without consuming it." (pg. 56)<br><br>**Sugary Beverages: "**Excessive consumption of these products can lead to cavities and deteriorate health, so it is important to reduce their consumption." (pg. 58)<br><br>**<u>How:</u>**<br>**Water:** "Drink daily 6-8 glasses of water complementary to the meals." (pg. 47)<br><br>**Sugary Beverages:** Avoid excessive consumption of sugar, sweets, carbonated drinks, and alcoholic beverages." (pg. 47)<br><br>**<u>Quantity/Frequency:</u>**<br>**Water:** "It is recommended to consume 2 liters of liquids, half of which should be water." (pg. 56)<br><br>**Sugary Beverages:** "The intake of simple sugars such as sucrose should be limited to a maximum of 10% of energy, except when it is necessary to increase the energy density." (pg. 86)

		**Image Content:**<br>Visual representation of **water**<br>Visual representation of **sugary beverages**
Canada (10)	Canada's Dietary Guidelines (2019)	**What:**<br>**Water:** "Water should be the beverage of choice." (pg. 9)<br><br>**Sugary Beverages:** "Processed or prepared food and beverages that contribute to excess sodium, free sugars, or saturated fat undermine healthy eating and should not be consumed regularly." (pg. 22)<br><br>**Where:**<br>**Water:** "Water should be the beverage of choice." (pg. 9) Key message/guideline.<br><br>**Sugary Beverages:** "Processed or prepared food and beverages that contribute to excess sodium, free sugars, or saturated fat undermine healthy eating and should not be consumed regularly." (pg. 22) Key message/guideline.<br><br>**Why:**<br>**Water:** "Health Canada recommends water as the beverage of choice to support health and promote hydration without adding calories to the diet. Water is vital for life—in fact it is the largest single component of the human body. It is essential for metabolic and digestive processes." (pg. 12)<br><br>**Sugary Beverages:** "Beverages that contain free sugars (including 100% fruit juice) have been associated with a higher risk of dental decay in children. Further, the intake of foods or beverages with added sugars has been associated with an increased risk of weight gain, overweight and obesity, and type 2 diabetes." (pg. 23)<br><br>**How:**<br>**Water:** "Make water your drink of choice." (pg. 49)<br><br>**Sugary Beverages:** "Replace sugary drinks with water." (pg. 49)<br><br>**Quantity/Frequency:**<br>**Water:** N/A<br><br>**Sugary Beverages:** "Recommended limits: Free sugars: Less than 10% of total energy intake." (pg. 23)<br><br>**Image Content:**<br>Visual representation of **water**

Chile* (11)	Food Guidelines for Chile (2022)	**<u>What:</u>**<br>**Water:** "Drink water several times a day, do not replace it with juices or beverages." (pg. 36)<br><br>**Sugary Beverages:** "Drink water several times a day, do not replace it with juices or beverages." (pg. 36)<br><br>**<u>Where:</u>**<br>**Water:** "Drink water several times a day, do not replace it with juices or beverages." (pg. 36) Key message/guideline.<br><br>**Sugary Beverages:** "Drink water several times a day, do not replace it with juices or beverages." (pg. 36) Key message/guideline.<br><br>**<u>Why:</u>**<br>**Water:**<br><br>"Water is essential for our organism, and it is present in the majority of the processes that allow us to live and carry out our activities." (pg. 37)<br><br>"Water promotes hydration and elasticity in the skin." (pg. 37)<br><br>**Sugary Beverages:** "Beverages, juices, sports drinks, and energy drinks, for the most part, contain sugar, increasing the amount of calories and sugars in your diet, promoting obesity, stimulating appetite, and leading to the onset of dental cavities and metabolic diseases such as diabetes and osteoporosis." (pg. 38)<br><br>**<u>How:</u>**<br>**Water:** "Drink water several times a day, do not replace it with juices or beverages." (pg. 36)<br><br>**Sugary Beverages:** "Drink water several times a day, do not replace it with juices or beverages." (pg. 36)<br><br>**<u>Quantity/Frequency:</u>**<br>**Water:** "Drink at least 6 to 8 glasses of water or more per day." (pg. 40)<br><br>**Sugary Beverages:** N/A<br><br>**<u>Image Content:</u>**<br>Visual representation of **water**<br>Visual representation of **sugary beverages**

Colombia* (5)	Food-based Dietary Guidelines for the Colombian Population Over 2 Years of Age (2020)	**What:**<br>**Water:** N/A<br><br>**Sugary Beverages: "**To maintain a healthy weight, reduce consumption of "packaged products," fast food, sodas, and sugary beverages." (pg. 280)<br><br>**Where:**<br>**Water:** N/A<br><br>**Sugary Beverages: "**To maintain a healthy weight, reduce consumption of "packaged products," fast food, sodas, and sugary beverages." (pg. 280) Key message/guideline.<br><br>**Why:**<br>**Water:** N/A<br><br>**Sugary Beverages:**<br><br>"The increase in the consumption of sugary beverages is a determining factor in weight gain in girls, boys, and adults." (pg. 92)<br><br>"Reducing the intake of sugary beverages promotes weight loss. Increased body fat is associated with high sugar intake." (pg. 92)<br><br>"Swapping/Replacing the consumption of a sugary beverage for a non-sugary one contributes to weight reduction." (pg. 92)<br><br>"For every increase in the consumption of sugary beverages of 12 ounces, there is an increase of 0.06 units in BMI among children and adolescents, and a weight gain of 0.12-0.22 kg in a year among adults." (pg. 92)<br><br>**How:**<br>**Water:** N/A<br><br>**Sugary Beverages:** "Limit the consumption of sodas and sugary drinks; prefer the consumption of natural juice low in sugar." (pg. 107)<br><br>**Quantity/Frequency:**<br>**Water:** N/A

		**Sugary Beverages:** N/A<br><br>**<u>Image Content:</u>**<br>Visual representation of **water**
Dominica (6)	Dominica Food Based Dietary Guidelines (2007)	**<u>What:</u>**<br>**Water:** "Drink water several times a day" (pg. 1)<br><br>**Sugary Beverages:** "Choose less sweet foods and drinks" (pg. 1)<br><br>**<u>Where:</u>**<br>**Water:** "Drink water several times a day" (pg. 1) Key message/guideline.<br><br>**Sugary Beverages:** "Choose less sweet foods and drinks" (pg. 1) Key message/guideline.<br><br>**<u>Why:</u>**<br>**Water:** N/A<br>**Sugary Beverages:** N/A<br><br>**<u>How:</u>**<br>**Water:** "Drink water several times a day" (pg. 1)<br><br>**Sugary Beverages:** "Choose less sweet foods and drinks" (pg. 1)<br><br>**<u>Quantity/Frequency:</u>**<br>**Water:** N/A<br>**Sugary Beverages:** N/A<br><br>**<u>Image Content:</u>**<br>No visual representation of water or sugary beverages
Ecuador* (11)	The Technical Document of the Food-Based Dietary Guidelines of Food of Ecuador (2020)	**<u>What:</u>**<br>**Water:** "Let's drink eight glasses of safe water daily for proper body function." (pg. 154)<br><br>**Sugary Beverages:** "Let's protect our health: let's avoid the consumption of processed products, fast food, and sugary drinks." (pg. 154)

		**<u>Where:</u>**<br>**Water:** "Let's drink eight glasses of safe water daily for proper body function." (pg. 154) Key message/guideline.<br><br>**Sugary Beverages:** "Let's protect our health: let's avoid the consumption of processed products, fast food, and sugary drinks." (pg. 154) Key message/guideline.<br><br>**<u>Why:</u>**<br>**Water:**<br><br>"Let's drink eight glasses of safe water daily for proper body function." (pg. 154)<br><br>"Let's drink water every day to hydrate and detoxify the body." (pg. 152)<br><br>**Sugary Beverages:** Table IV.I Development of technical recommendations - problems by excess (pg. 68)<br><br>**<u>How:</u>**<br>**Water:** "Consume safe water." (pg. 73)<br><br>**Sugary Beverages:** "Let's protect our health: let's avoid the consumption of processed products, fast food, and sugary drinks." (pg. 154)<br><br>**<u>Quantity/Frequency:</u>**<br>**Water:** "Let's drink eight glasses of safe water daily for proper body function." (pg. 154)<br><br>**Sugary Beverages:** N/A<br><br>**<u>Image Content:</u>**<br>Visual representation of **water**<br>Visual representation of **sugary beverages**
El Salvador* (9)	Dietary Guidelines for Salvadorian Families (2012)	**<u>What:</u>**<br>**Water:** "Consume at least 6 to 8 glasses of water per day" (pg. 18)<br><br>**Sugary Beverages:**<br><br>"Instead of carbonated drinks order/request water or drinks made with natural fruits." (pg. 20)<br><br>"Avoid eating sugary foods and drinks, fried foods, sausages, sweets, fast foods and canned foods." (pg. 17)

		**<u>Where:</u>**<br>**Water:** "Consume at least 6 to 8 glasses of water per day" (pg. 18) Key message/guideline.<br><br>**Sugary Beverages:** "Avoid eating sugary foods and drinks, fried foods, sausages, sweets, fast foods and canned foods." (pg. 17) Key message/guideline.<br><br>**<u>Why:</u>**<br>**Water:**<br><br>"Some special physiological situations such as pregnancy, breastfeeding, and during sports require an increased intake of water. Likewise, in certain illnesses such as diarrhea, vomiting, and fever, which can lead to dehydration, it is necessary to hydrate the person frequently." (pg. 18-19)<br><br>**Sugary Beverages: "**Other beverages such as soda and beer provide extra calories." (pg. 19)<br><br>**<u>How:</u>**<br>**Water:** "Instead of carbonated drinks order/request water or drinks made with natural fruits." (pg. 20)<br><br>**Sugary Beverages:** "Instead of carbonated drinks order/request water or drinks made with natural fruits." (pg. 20)<br><br>**<u>Quantity/Frequency:</u>**<br>**Water:** "For children, we recommend consuming 6 glasses of water, and for adults, 8 glasses of water throughout the day." (pg. 18)<br><br>**Sugary Beverages:** N/A<br><br>**<u>Image Content:</u>**<br>N/A
Grenada (9)	Healthy Choices for Healthy Living: Guidelines for Grenadians (2020)	**<u>What:</u>**<br>**Water:** "Drink more water every day, it helps your body function better."(pg. 1)<br><br>**Sugary Beverages:** "Avoid highly processed foods, snacks and drinks; they are high in fat, salt and sugar." (pg. 1)

		**<u>Where:</u>**<br>**Water:** "Drink more water every day, it helps your body function better." (pg. 1) Key message/guideline.<br><br>**Sugary Beverages:** "Avoid highly processed foods, snacks and drinks; they are high in fat, salt and sugar." (pg. 1) Key message/guideline.<br><br>**<u>Why:</u>**<br>**Water:** "Drink more water every day, it helps your body function better." (pg. 1)<br><br>**Sugary Beverages:** N/A<br><br>**<u>How:</u>**<br>**Water:** "Drink more water every day, it helps your body function better." (pg. 1)<br><br>**Sugary Beverages:** "Avoid highly processed foods, snacks and drinks; they are high in fat, salt and sugar." (pg. 1)<br><br>**<u>Quantity/Frequency:</u>**<br>**Water:** N/A<br>**Sugary Beverages:** N/A<br><br>**<u>Image Content:</u>**<br>Visual representation of **water**<br>Visual representation of **sugary beverages**
Guatemala* (10)	Dietary Guidelines for Guatemala Recommendations for Healthy Eating (2012)	**<u>What:</u>**<br>**Water: "**Drink at least eight glass of water per day." (pg. 35)<br><br>**Sugary Beverages:** "Avoid the consumption of carbonated waters, energy drinks, artificially flavored bottled drinks, packaged juices, etc., because they contain excessive sugar, preservatives, colorants that are harmful to health." (pg. 36)<br><br>**<u>Where:</u>**<br>**Water: "**Drink 8 glasses of safe water (boiled or chlorinated) per day." (pg. 35) Bolded.<br><br>**Sugary Beverages:** "Avoid the consumption of carbonated waters, energy drinks, artificially flavored bottled drinks, packaged juices, etc., because they contain excessive sugar, preservatives, colorants that are harmful to health." (pg. 36) Highlighted through text box.

		<u>**Why:**</u><br>**Water:** "Water is an essential element for the body; it helps maintain body temperature, transport nutrients within the body, and eliminate toxins from the body." (pg. 35)<br><br>**Sugary Beverages:** "Avoid the consumption of carbonated waters, energy drinks, artificially flavored bottled drinks, packaged juices, etc., because they contain excessive sugar, preservatives, colorants that are harmful to health." (pg. 36)<br><br><u>**How:**</u><br>**Water: "**Drink at least eight glass of water per day." (pg. 13)<br><br>**Sugary Beverages:** "Avoid the consumption of carbonated waters, energy drinks, artificially flavored bottled drinks, packaged juices, etc., because they contain excessive sugar, preservatives, colorants that are harmful to health." (pg. 36)<br><br><u>**Quantity/Frequency:**</u><br>**Water:** "Drink 8 glasses of safe water (boiled or chlorinated) per day." (pg. 35)<br><br>**Sugary Beverages:** N/A<br><br><u>**Image Content:**</u><br>Visual representation of **water**
Honduras* (10)	Dietary Guidelines for Honduras Tips for Healthy Eating (2013)	<u>**What:**</u><br>**Water:** "Drink at least 8 glasses of water a day for the proper functioning of your body." (pg. 15)<br><br>**Sugary Beverages:** "Avoid consuming sodas, energy drinks, bottled beverages, packaged juices, and soft drinks, etc., as they generally contain large amounts of sugar, colorants, and preservatives that are harmful to health." (pg. 34)<br><br><u>**Where:**</u><br>**Water:** "Drink at least 8 glasses of water a day for the proper functioning of your body." (pg. 15) Key message/guideline.<br><br>**Sugary Beverages:** N/A

		**<u>Why:</u>**<br>**Water:** "Water helps with digestion and improves nutrient absorption; it helps to eliminate toxins and waste products from the body; water regulates body temperature; it keeps the skin hydrated; water helps lubricate muscles and joints, reducing cramps; it prevents the formation of kidney stones." (pg. 33)<br><br>**Sugary Beverages**: "Avoid consuming sodas, energy drinks, bottled beverages, packaged juices, and soft drinks, etc., as they generally contain large amounts of sugar, colorants, and preservatives that are harmful to health." (pg. 34)<br><br>**<u>How:</u>**<br>**Water:** "Drink at least 8 glasses of water a day for the proper functioning of your body." (pg. 15)<br><br>**Sugary Beverages:** "Avoid consuming sodas, energy drinks, bottled beverages, packaged juices, and soft drinks, etc., as they generally contain large amounts of sugar, colorants, and preservatives that are harmful to health." (pg. 34)<br><br>**<u>Quantity/Frequency:</u>**<br>**Water:** "It is recommended to drink approximately 6 to 8 glasses of water per day." (pg. 33)<br><br>**Sugary Beverages:** N/A<br><br>**<u>Image Content:</u>**<br>Visual representation of **water**<br>Visual representation of **sugary beverages**
Mexico* (10)	Food Guides for the Mexican Population (2023)	**<u>What:</u>**<br>**Water:** "Let's drink natural water throughout the day and with all our meals, instead of sugary drinks like sodas, juices, water mixed with powdered packets, and sports drinks, which can harm our health." (pg. 59)<br><br>**Sugary Beverages:** "Let's drink natural water throughout the day and with all our meals, instead of sugary drinks like sodas, juices, water mixed with powdered packets, and sports drinks, which can harm our health." (pg. 59)<br><br>**<u>Where:</u>**<br>**Water:** "Let's drink natural water throughout the day and with all our meals, instead of sugary drinks like sodas, juices, water mixed with powdered packets, and sports drinks, which can harm our health." (pg. 59) Key message/guideline.

		**Sugary Beverages:** “Let's drink natural water throughout the day and with all our meals, instead of sugary drinks like sodas, juices, water mixed with powdered packets, and sports drinks, which can harm our health.” (pg. 59) Key message/guideline.<br><br>**<u>Why:</u>**<br>**Water:** “Water is essential for health. Drinking natural water is the best way to hydrate.” (pg. 61)<br><br>**Sugary Beverages:**<br>“The consumption of sugary drinks is strongly linked to childhood obesity. For each glass of sugary drink consumed per day, the likelihood of a child being obese increases by 60%.” (pg. 61)<br><br>“Reducing the consumption of sugary drinks reduces the risk of overweight and obesity, diabetes, cardiovascular disease, and dental caries.” (pg. 61)<br><br>“The consumption of drinks with artificial sweeteners (for example, "diet" sodas) is not recommended, as they also damage teeth and bones, and can lead to heart disease, diabetes, and other illnesses.” (pg. 61)<br><br>**<u>How:</u>**<br>**Water:**<br><br>“Take meals only with natural water. Aim to always have a pitcher of natural water on the table and within reach of children.” (pg. 63)<br><br>“Drink water during and after physical activity/work outs” (pg. 64)<br><br>**Sugary Beverages:** “Let's drink natural water throughout the day and with all our meals, instead of sugary drinks like sodas, juices, water mixed with powdered packets, and sports drinks, which can harm our health.” (pg. 59)<br><br>**<u>Quantity/Frequency:</u>**<br>**Water:** “The recommendation from these guidelines for teenagers and adults is to drink 8 to 13 glasses of plain water per day, depending on gender and age. For school-age girls and boys, it is recommended to drink 5 to 8 glasses of water depending on gender.” (pg. 63)<br><br>**Sugary Beverages:** N/A<br><br>**<u>Image Content:</u>**

		Visual representation of **water**
Panama* (10)	The Guidelines for Panama (2013)	**<u>What:</u>**<br>**Water:** "Drink water throughout the day and enjoy it." (pg. 13)<br><br>**Sugary Beverages:** "Avoid sodas, iced tea, and sugary drinks. Prefer water natural juices without added sugar." (pg. 10)<br><br>**<u>Where:</u>**<br>**Water:** "Drink water throughout the day and enjoy it." (pg. 13) Key message/guideline.<br><br>**Sugary Beverages:** "Avoid sodas, iced tea, and sugary drinks. Prefer water natural juices without added sugar." (pg. 10) Key message/guideline.<br><br>**<u>Why:</u>**<br>**Water:** "Water, an essential liquid for life, transports within the human body all the nutrients we consume through food. More than half of the human body is composed of water." (pg. 13)<br><br>**Sugary Beverages:** "Daily or frequent consumption of sodas and sugary drinks contributes to the development of obesity, diabetes, hypertension, high triglycerides, cardiovascular, and renal diseases." (pg. 10)<br><br>**<u>How:</u>**<br>**Water:** "Drink water throughout the day and enjoy it." (pg. 13)<br><br>**Sugary Beverages:** "Avoid sodas, iced tea, and sugary drinks. Prefer water natural juices without added sugar." (pg. 10)<br><br>**<u>Quantity/Frequency:</u>**<br>**Water:** "Drink at least 8 glasses (2 liters) of water per day." (pg. 13)<br><br>**Sugary Beverages:** N/A<br><br>**<u>Image Content:</u>**<br>Visual representation of **water**
Peru* (12)	Dietary Guidelines for the Peruvian Population (2020)	**<u>What:</u>**<br>**Water:** "Stay healthy by drinking 6 to 8 glasses of water a day." (pg. 33)

		**Sugary Beverages:** "Take care of your health; avoid overweight by reducing the consumption of sugars in your meals and drinks." (pg. 30)<br><br>**<u>Where:</u>**<br>**Water:** "Stay healthy by drinking 6 to 8 glasses of water a day." (pg. 33) Key message/guideline.<br><br>**Sugary Beverages:** "Take care of your health; avoid overweight by reducing the consumption of sugars in your meals and drinks." (pg. 30) Key message/guideline.<br><br>**<u>Why:</u>**<br>**Water:** "Water is essential for the proper functioning of our body, and since it is not stored in the body, it needs to be replenished throughout the day. Therefore, a minimum intake of 2 to 2.5 liters of water per day is recommended." (pg. 33)<br><br>**Sugary Beverages:** "Take care of your health; avoid overweight by reducing the consumption of sugars in your meals and drinks." (pg. 30)<br><br>**<u>How:</u>**<br>**Water:**<br><br>"Avoid packaged sugary drinks and consume natural water." (pg. 26)<br><br>**Sugary Beverages:**<br>"Avoid packaged sugary drinks and consume natural water." (pg. 26)<br><br>"Replace sodas, packaged fruit juices, and packaged sugary juices with water or natural sugar-free juices." (pg. 30)<br><br>**<u>Quantity/Frequency:</u>**<br>**Water:** "Stay healthy by drinking 6 to 8 glasses of water a day." (pg. 33)<br><br>**Sugary Beverages: "**According to the WHO, the sugar intake should be reduced to at last 10% of the total energy that is needed daily. This is equivalent to 10 teaspoons per day." (pg. 30)<br><br>**<u>Image Content:</u>**<br>Visual representation of **water**<br>Visual representation of **sugary beverages**

Saint Kitts and Nevis (8)	Food Based Dietary Guidelines for St. Kitts and Nevis (2010)	**What:**<br>**Water:** "Replace sweet drinks with water." (pg. 15)<br><br>**Sugary Beverages:** "Limit the use of foods and drinks with added salt and sugar." (pg. 15)<br><br>**Where:**<br>**Water:** N/A<br><br>**Sugary Beverages:** "Limit the use of foods and drinks with added salt and sugar." Key message/guideline. (pg. 15)<br><br>**Why:**<br>**Water:** "Water functions: serves as a transport systems for all nutrients; washes out waste from the body; helps body temperature to keep stable."(pg. 12)<br><br>**Sugary Beverages:** "Benefits: better weight control; good control of blood pressure levels; better control of blood sugar levels; less problems with dental caries/tooth decay; more energy and vitality." (pg. 15)<br><br>**How:**<br>**Water:** "Replace sweet drinks with water." (pg. 15)<br><br>**Sugary Beverages:** "Limit the use of foods and drinks with added salt and sugar." (pg. 15)<br><br>**Quantity/Frequency:**<br>**Water:** N/A<br><br>**Sugary Beverages:** Recommendations for sugars and sweeteners (pg. 23)<br><br>**Image Content:**<br>No visual representation of water or sugary beverages
Saint Vincent and the Grenadines (10)	Food Based Dietary Guidelines of St. Vincent and the Grenadines (2021)	**What:**<br>**Water:** "Water is essential! Drink at least 8 glasses daily instead of sugary drinks." (pg. 2)<br><br>**Sugary Beverages: "**Water is essential! Drink at least 8 glasses daily instead of sugary drinks." (pg. 2)<br><br>**Where:**

		**Water:** "Water is essential! Drink at least 8 glasses daily instead of sugary drinks." Key message/guideline. (pg. 2)<br><br>**Sugary Beverages:** "Water is essential! Drink at least 8 glasses daily instead of sugary drinks." Key message/guideline. (pg. 2)<br><br>**<u>Why:</u>**<br>**Water:** "Water is the liquid of life, it is a calorie-free way to quench your thirst, consume it from clean, safe sources." (pg. 2)<br><br>**Sugary Beverages:** N/A<br><br>**<u>How:</u>**<br>**Water:** "Water is essential! Drink at least 8 glasses daily instead of sugary drinks." (pg. 2)<br><br>**Sugary Beverages:** "Water is essential! Drink at least 8 glasses daily instead of sugary drinks." (pg. 2)<br><br>**<u>Quantity/Frequency:</u>**<br>**Water:** "Water is essential! Drink at least 8 glasses daily instead of sugary drinks. At least 8 ounces per glass" (pg. 2)<br><br>**Sugary Beverages:** N/A<br><br>**<u>Image Content:</u>**<br>Visual representation of **water**<br>Visual representation of **sugary beverages**
United States (8 jurisdictions) (7)	Dietary Guidelines for Americans (2020-2025)	**<u>What:</u>**<br>**Water:** "Beverages that are calorie-free—especially water—or that contribute beneficial nutrients, such as fat-free and low-fat milk and 100% juice, should be the primary beverages consumed." (pg. 35)<br><br>**Sugary Beverages:**<br>"Limit foods and beverages higher in added sugars, saturated fat, and sodium, and limit alcoholic beverages." (pg. 17)<br><br>"Sugar-sweetened beverages (e.g., regular soda, juice drinks [not 100% fruit juice], sports drinks, and flavored water with sugar) should not be given to children younger than age 2." (pg. 62)

		"Intake of sugar-sweetened beverages should be limited to small amounts and most often replaced with beverage options that contain no added sugars, such as water." (pg. 103)<br><br>**<u>Where:</u>**<br>**Water:** N/A<br><br>**Sugary Beverages:** "Limit foods and beverages higher in added sugars, saturated fat, and sodium, and limit alcoholic beverages." Key message/guideline. (pg. 17)<br><br>**<u>Why:</u>**<br>**Water:** "It is important that older adults drink plenty of water to prevent dehydration and aid in the digestion of food and absorption of nutrients." (pg. 129) (this recommendation is only clearly for older adults)<br><br>**Sugary Beverages:**<br><br>"Frequent consumption of these and other beverages containing added sugars can contribute to excess calorie intake" (pg. 103)<br><br>"Sugar-sweetened beverages (e.g., soda, sports drinks, energy drinks, fruit drinks) and sweetened coffees and teas (including ready-to-drink varieties) contribute over 40 percent of daily intake of added sugars." (pg. 103)<br><br>**<u>How:</u>**<br>**Water:** N/A<br><br>**Sugary Beverages:** "Limit foods and beverages higher in added sugars, saturated fat, and sodium, and limit alcoholic beverages." (pg. 17)<br><br>**<u>Quantity/Frequency:</u>**<br>**Water:** N/A<br><br>**Sugary Beverages:** "Added sugars—Less than 10 percent of calories per day starting at age 2. Avoid foods and beverages with added sugars for those younger than age 2" (pg. 18)<br><br>**<u>Image Content:</u>** No visual representation of water or sugary beverages

*FBDG technical document searched using Spanish translation of keywords: “water,” “hydration,” “sugar-sweetened beverage,” “sugary beverages,” “beverages” and “drink” (i.e., “agua,” “hidratación,” “bebida,” “azucarada,” “bebida endulzada” and “bebida”. The terms “refresco” and “soda” were also searched among the FBDGs published in Spanish.)
*****These were selected examples from FBDGs and are not comprehensive of every relevant recommendation**

Supplemental Table 8. FBDG Healthy Hydration Recommendations for Countries with Sugary Beverage Tax Legislation in the WHO Southeast Asia Region.

**WHO Region Country (Policy Coherence Score)**	**National Dietary Guidelines/graphic FBDG (year)**	**FBDG Healthy Hydration Recommendations*****
**WHO Southeast Asian Region (*n*=7)**		
Bangladesh (10)	Dietary Guidelines for Bangladesh (2013)	<u>**What:**</u><br>**Water:** “Drink plenty of water daily.” (pg. 20)<br><br>**Sugary Beverages:** “Take less sugar, sweets or sweetened drinks.” (pg. 18)<br><br><u>**Where:**</u><br>**Water:** “Drink plenty of water daily.” Key message/guideline. (pg. 20)<br><br>**Sugary Beverages:** “Take less sugar, sweets or sweetened drinks.” Key message/guideline. (pg. 18)<br><br><u>**Why:**</u><br>**Water:** “Water is an essential nutrient. All biochemical reactions occur within the water components in our body. Water is also required for digestion, absorption, transportation, dissolving nutrients, elimination of waste products and thermos-regulation.” (pg. 20)<br><br>**Sugary Beverages:** “Sugar is mainly used to sweeten food and beverages like tea, coffee and desserts. A high intake of sugar may also contribute to excess energy intake leading to obesity, which in turn is a risk factor for coronary heart disease and diabetes mellitus.” (pg. 18)<br><br><u>**How:**</u><br>**Water:** “Drink plenty of water daily.” (pg. 20)<br><br>**Sugary Beverages:** “Take less sugar, sweets or sweetened drinks.” (pg. 18)

		**Quantity/Frequency:**<br>**Water:** "Drink 1.5 to 3.5 liters (6-14 glasses) pure drinking water daily." (pg. 20)<br><br>**Sugary Beverages:**<br><br>"Less than 10% of total energy should be provided from free sugars" (pg. 18)<br><br>"Consume not more than 25g (5 teaspoons) of sugar per day." (pg. 19)<br><br>**Image Content:**<br>No visual representation of water or sugary beverages
India (9)	Dietary Guidelines for Indians: A Manual (2011)	**What:**<br>**Water:** "Drink plenty of water and take beverages in moderation" (pg. 10)<br><br>**Sugary Beverages:** "Drink plenty of water and take beverages in moderation" (pg. 10)<br><br>**Where:**<br>**Water:** "Drink plenty of water and take beverages in moderation" (pg. 10) Key message/guideline.<br><br>**Sugary Beverages:** "Drink plenty of water and take beverages in moderation" (pg. 10) Key message/guideline.<br><br>**Why:**<br>**Water:** "Water is the most important nutrient of all and helps in the upkeep of our health" (pg. 70)<br><br>**Sugary Beverages:** N/A<br><br>**How:**<br>**Water:**<br>"Drink enough of safe and wholesome water to meet daily fluid requirements." (pg. 74)<br><br>"Consume adequate water to avoid dehydration." (pg. 81)<br><br>**Sugary Beverages:** "Drink natural and fresh fruit juices instead of carbonated beverages." (pg. 74)<br><br>**Quantity/Frequency:**<br>**Water:** "A normal healthy person needs to drink about 8 glasses (2 litre) of water per day." (pg. 70)

		**Sugary Beverages:** Table describing grams/portion of sugar (pg. 86)<br><br>**Image Content:**<br>No visual representation of water or sugary beverages
Maldives (11)	Food Based Dietary Guidelines for Maldives (2019)	**What:**<br>**Water:** "Drink plenty of water and choose water over sugary drinks" (pg. 13)<br><br>**Sugary Beverages:** "Drink plenty of water and choose water over sugary drinks" (pg. 13)<br><br>**Where:**<br>**Water:** "Drink plenty of water and choose water over sugary drinks" (pg. 13) Key message/guideline.<br><br>**Sugary Beverages:** "Drink plenty of water and choose water over sugary drinks" (pg. 13) Key message/guideline.<br><br>**Why:**<br>**Water:** "Water is involved in many functions of the body hence is essential for life." (pg. 41)<br><br>**Sugary Beverages:** "Consuming too much sugar and too many foods and drinks high in sugar can lead to weight gain, which in turn increases the risk of heart disease, type 2 diabetes, stroke and some cancers. It is also linked to tooth decay in both children and adults." (pg. 33)<br><br>**How:**<br>**Water:** "Drink plenty of water and choose water over sugary drinks." (pg. 40)<br><br>**Sugary Beverages:**<br><br>"Drink plenty of water and choose water over sugary drinks." (pg. 40)<br><br>"Always choose water as your preferred drink rather than juices, fizzy drinks or other sugary drinks." (pg. 41)<br><br>"Avoid excessive intake of sugar sweetened beverages." (recommendation for pregnancy) (pg. 55)<br><br>**Quantity/Frequency:**<br>**Water:** "Adults under normal circumstances need 1.5 to 2 litres of fluid per day." (pg. 41)

		**Sugary Beverages:** "Reducing the intake of free sugars to less than 25 grams or 6 teaspoons per day (5% of total energy intake) would provide additional health benefits." (pg. 33)<br><br>**<u>Image Content:</u>**<br>Visual representation of **water**
Nepal** (6)	Food-Based Dietary Guidelines for Nepalese (2012)	**<u>What:</u>**<br>**Water:** "Eat clean foods and drink safe drinking water." FAO website<br><br>**Sugary Beverages: "**Consume less sugar, sweets and sweetened drinks." FAO website<br><br>**<u>Where:</u>**<br>**Water:** "Eat clean foods and drink safe drinking water." Key message/guideline. FAO website<br><br>**Sugary Beverages: "**Consume less sugar, sweets and sweetened drinks." Key message/guideline. FAO website<br><br>**<u>Why:</u>**<br>**Water:** N/A<br>**Sugary Beverages:** N/A<br><br>**<u>How:</u>**<br>**Water:** "Eat clean foods and drink safe drinking water." FAO website<br><br>**Sugary Beverages: "**Consume less sugar, sweets and sweetened drinks." FAO website<br><br>**<u>Quantity/Frequency:</u>**<br>**Water:** N/A<br>**Sugary Beverages:** N/A<br><br>**<u>Image Content:</u>**<br>N/A
Sri Lanka (11)	Food-Based Dietary Guidelines for Sri Lankans – Practitioner's Handbook (2021)	**<u>What:</u>**<br>**Water:** "Water is the healthiest drink: Drink 8 to 10 glasses (1.5-2.0 liters) throughout the day." (pg. 59)<br><br>**Sugary Beverages:** "Limit sugary drinks, biscuits, cakes, sweets and sweeteners." (pg. 55)<br><br>**<u>Where:</u>**

		**Water:** "Water is the healthiest drink: Drink 8 to 10 glasses (1.5-2.0 liters) throughout the day." Key message/guideline. (pg. 59)<br><br>**Sugary Beverages:** "Limit sugary drinks, biscuits, cakes, sweets and sweeteners." Key message/guideline. (pg. 55)<br><br>**<u>Why:</u>**<br>**Water:** "It is essential to drink water throughout the day to balance the amount of water losses through sweat, urine and feces. Water accounts for approximately 70% of body weight. It plays an integral role in many vital functions of our body. It is a part of blood and other body fluids and helps in excreting body wastes and maintaining body temperature." (pg. 59)<br><br>**Sugary Beverages:**<br><br>"Consumption of sugary sweetened beverages (SSB), biscuits, cakes and other sweets promotes excess energy intake. The sugar (table sugar/sucrose) that we add to food and beverages provides empty calories which do not have other nutrients." (pg. 55)<br><br>"Excess sugar is associated with weight gain and tooth decay." (pg. 55)<br><br>"Weight gain in turn predisposes to other noncommunicable diseases such as diabetes and cardiovascular diseases." (pg. 55)<br><br>**<u>How:</u>**<br>**Water:** "Water is the healthiest drink: Drink 8 to 10 glasses (1-5-2.0 liters) throughout the day." (pg. 59)<br><br>**Sugary Beverages:** "Limit sugary drinks, biscuits, cakes, sweets and sweeteners." (pg. 55)<br><br>**<u>Quantity/Frequency:</u>**<br>**Water:** "Water is the healthiest drink: Drink 8 to 10 glasses (1-5-2.0 liters) throughout the day." (pg. 59)<br><br>**Sugary Beverages:**<br><br>"Limit intake of sugar: Do not exceed 25g/ 6 teaspoons per person per day." (pg. 55)

		"Reduce the intake of calories from free sugars* to less than 10% of the daily recommended total calorie requirement. A further reduction to below 5% (roughly 25 grams or 6 teaspoons) provides additional health benefits." (pg. 55)<br><br>Cut-off values for sugar content in sugar sweetened beverages. (pg. 84)<br><br>**<u>Image Content:</u>**<br>Visual representation of **sugary beverages**
Thailand (1)	Food-Based Dietary Guideline for Thai (1998, Second Edition 2007)	**<u>What:</u>**<br>**Water:** N/A<br>**Sugary Beverages:** N/A<br><br>**<u>Where:</u>**<br>**Water:** N/A<br>**Sugary Beverages:** N/A<br><br>**<u>Why:</u>**<br>**Water:** N/A<br>**Sugary Beverages:** N/A<br><br>**<u>How:</u>**<br>**Water:** N/A<br>**Sugary Beverages:** N/A<br><br>**<u>Quantity/Frequency:</u>**<br>**Water:** N/A<br><br>**Sugary Beverages:** "One should obtain no more than 10% of his/her total food energy from sugar." (pg. 28)<br><br>**<u>Image Content:</u>**<br>No visual representation of water or sugary beverages
Democratic Republic of Timor-Leste (0)	N/A	

**FBDG technical document did not allow for keyword searches or was unavailable. Translated key messages and guidelines extracted from FAO website.
***These were selected examples from FBDGs and are not comprehensive of every relevant recommendation**

Supplemental Table 9. FBDG Healthy Hydration Recommendations for Countries with Sugary Beverage Tax Legislation in the WHO Western Pacific Region.

**WHO Region Country (Policy Coherence Score)**	**National Dietary Guidelines/graphic FBDG (year)**	**FBDG Healthy Hydration Recommendations*** **
**WHO Western Pacific Region (n=19)**		
American Samoa (0)	N/A	
Brunei (12)	National Dietary Guidelines for Healthy Eating Brunei Darussalam (2020)	**What:**<br>**Water:** "Drink at least eight glasses of water a day." (pg. 102)<br><br>**Sugary Beverages:** "Limit intake of sugar-sweetened beverages." (pg. 102)<br><br>**Where:**<br>**Water:** "Drink at least eight glasses of water a day." (pg. 102) Key message/guideline.<br><br>**Sugary Beverages:** "Limit intake of sugar-sweetened beverages." (pg. 102) Key message/guideline.<br><br>**Why:**<br>**Water:** "Choose water instead of sugary drinks during main meals and when eating out. This helps to save money and reduce calorie intake." (pg. 102)<br><br>**Sugary Beverages:**<br><br>"Due to the less satiety effect of sugar-sweetened beverages, those who consumed them tend to continue consuming other foods or beverages, leading to a positive energy balance." (pg. 40)<br><br>"Numerous evidence suggested an association between sugar-sweetened beverages and type 2 diabetes." (pg. 101)<br><br>**How:**<br>**Water:**<br><br>"Drink at least eight glasses of water a day." (pg. 102); "Choose water instead of sugary drinks during main meals and when eating out." (pg. 102); "Encourage children to drink water if thirsty." (pg. 102); "Always carry a

		water bottle including schoolchildren." (pg. 102) ; "Make water as the main choice during family gathering such as birthday celebrations, weddings and any formal events." (pg. 102); "Add a wedge of lime or lemon to water for taste if necessary." (pg. 102)<br><br>**Sugary Beverages:**<br><br>"Avoid of limit drinks with high sugar (i.e., containing more than 6 g of sugar per 100 ml)." (pg. 102)<br><br>**<u>Quantity/Frequency:</u>**<br><br>**Water:** "Water is the best choice- drink at least eight glasses (2 litre) of water a day." (pg. 102)<br><br>**Sugary Beverages:** "Avoid or limit drinks with high sugar (i.e., containing more than 6 g of sugar per 100 ml)." (pg. 102)<br><br>**<u>Image Content:</u>**<br>Visual representation of **water**<br>Visual representation of **sugary beverages**
Cambodia (4)	Development of Recommended Dietary Allowance and Food-Based Dietary Guidelines for School-Aged Children in Cambodia (2017)	**<u>What:</u>**<br>**Water:** N/A<br><br>**Sugary Beverages:** "Reduce food high in salt, sugar and fat: Try to limit sweetened drinks. You can choose to stay healthy through selecting foods that are low in fat, salt and sugar." (pg. 53)<br><br>**<u>Where:</u>**<br>**Water:** N/A<br><br>**Sugary Beverages:** "Reduce food high in salt, sugar and fat: Try to limit sweetened drinks. You can choose to stay healthy through selecting foods that are low in fat, salt and sugar." Key message/guideline. (pg. 53)<br><br>**<u>Why:</u>**<br>**Water:** N/A<br><br>**Sugary Beverages:** "Many people eat too much saturated fat (especially animal fat), added salt (fish sauce and salty condiments) and added sugar (sugary beverages). Reducing these by small amounts can make use healthier by helping use manage our weight and reducing our risk of diseases." (pg. 60)

		**How:**<br>**Water:** N/A<br><br>**Sugary Beverages:** "Reduce food high in salt, sugar and fat: Try to limit sweetened drinks. You can choose to stay healthy through selecting foods that are low in fat, salt and sugar." (pg. 53)<br><br>**Quantity/Frequency:**<br>**Water:** N/A<br>**Sugary Beverages:** N/A<br><br>**Image Content:**<br>No visual representation of water or sugary beverages
Cook Islands (0)	N/A	
Fiji (10)	Food & Health Guidelines for Fiji (2013)	**What:**<br>**Water:** "Drink clean and safe water." (pg. 20)<br><br>**Sugary Beverages:** "Choose and prepare food and drinks with less salt, sugar, fat and oil." (pg. 4)<br><br>**Where:**<br>**Water:** "Drink clean and safe water." (pg. 20) Key message/guideline.<br><br>**Sugary Beverages:** "Choose and prepare food and drinks with less salt, sugar, fat and oil." (pg. 4) Key message/guideline.<br><br>**Why:**<br>**Water:**<br>"Water quenches your thirst and helps you feel full without any added calories." (pg. 11)<br><br>"Water is valuable for life. It is an essential nutrient which fulfills important functions in our body." (pg. 20)<br><br>**Sugary Beverages:** "Simple sugars such as table sugar and sugary foods and fizzy drinks raise blood sugar levels very quicky which is not good." (pg. 4)<br><br>**How:**<br>**Water:**<br>"Drink fresh water, coconut juice, unsweetened fruit juice, low fat or soy milk instead." (pg. 5)

		"Drink water often." (pg. 11)<br><br>**Sugary Beverages:**<br>"Reduce drinking fizzy drinks and other sugary soft drinks." (pg. 5)<br><br>"Fizzy and sugary drinks are also not a good way to get your fluid. The high sugar content can cause unwanted weight gain, or take the place of more nutritious food in your diet." (pg. 20)<br><br>**<u>Quantity/Frequency:</u>**<br>**Water:** "Therefore, we must drink at least 1 ½ to 2 litres of water a day (more for adults and older children)." (pg. 20)<br><br>**Sugary Beverages:** N/A<br><br>**<u>Image Content:</u>**<br>Visual representation of **water**
French Polynesia (0)	N/A	
Kiribati (0)	N/A	
Malaysia (11)	Malaysian Dietary Guidelines (2020)	**<u>What:</u>**<br>**Water:** "Drink plenty of water daily." (pg. 196)<br><br>**Sugary Beverages:** "Limit sugar intake in foods and beverages." (pg. 178)<br><br>**<u>Where:</u>**<br>**Water:** "Drink plenty of water daily." (pg. 196) Key message/guideline.<br><br>**Sugary Beverages:** "Limit sugar intake in foods and beverages." (pg. 178) Key message/guideline.<br><br>**<u>Why:</u>**<br>**Water:**<br>"Water is essential for many body functions for example regulating body temperature and digestion." (pg. 4)<br><br>"Plain water is encouraged to be taken to replace sugary drinks to help reduce calorie intake and control body weight and dental caries" (pg. 26)

		**Sugary Beverages:** "Studies proved that regular sugar sweetened beverages (SSB) consumption has been positively associated with increased body weight and risk of obesity" (pg. 181)<br><br>**<u>How:</u>**<br>**Water:**<br>"Drink plain water or unsweetened beverages with the meal." (pg. 12)<br><br>"Always choose plain water" (pg. 185)<br><br>**Sugary Beverages:**<br>"Drink plain water instead of sugary drinks." (pg. 46)<br><br>"Request for plan water to go with your meals instead of sweet sugary drinks such as cordials, Air Batu Campur (ABC), carbonated sodas, cendol, drinks with whipped cream, milk shakes and bubble tea." (pg. 88)<br><br>"Limit intake of ultra-processed beverages such as carbonated and non-carbonated sugar-sweetened beverages (soft drink, syrup and cordial)." (pg. 185)<br><br>"Limit intake of beverages with added sugar and sweetened condensed milk (teh tarik, coffee, air batu campur, bubble tea) and premix beverages." (pg. 185)<br><br>"Avoid consuming sugary beverages during morning and afternoon snack and close to bedtime." (pg. 185)<br><br>"Limit intake of beverages that sugar listed as the first ingredient in the ingredient list on the food label." (pg. 185)<br><br>**<u>Quantity/Frequency:</u>**<br>**Water:** "Drink at least 6-8 glasses of plain water daily." (pg. 26)<br><br>**Sugary Beverages:** "Nevertheless, it is strongly recommended that free sugar intake should remain below 10% of total energy intake to prevent excess of calories" (pg. 184)<br><br>**<u>Image Content:</u>**<br>Visual representation of **water** (The pyramid within the guidelines is different than the independent image, as it has a glass of water next to the pyramid).

Marshall Islands (10)	RMI Guidelines for Healthy Living: A Handbook for Community Members (2022)	**What:**<br>**Water:** "Drink clean, safe water. Avoid soda or sugar-sweetened beverages." (pg. 13)<br><br>**Sugary Beverages:** "Drink clean, safe water. Avoid soda or sugar-sweetened beverages." (pg. 13)<br><br>**Where:**<br>**Water:** "Drink clean, safe water. Avoid soda or sugar-sweetened beverages." (pg. 13) Key message/guideline.<br><br>**Sugary Beverages:** "Drink clean, safe water. Avoid soda or sugar-sweetened beverages." (pg. 13) Key message/guideline.<br><br>**Why:**<br>**Water:** "Water helps to keep the body cool, remove waste, lubricate joints, digest food, prevent constipation and carry oxygen and nutrients to body cells." (pg. 13)<br><br>**Sugary Beverages:** "A high intake of free sugars in the form of sugar-sweetened beverages is linked to obesity and dental caries." (pg. 10)<br><br>**How:**<br>**Water:** "Drink clean, safe water. Avoid soda or sugar-sweetened beverages." (pg. 13)<br><br>**Sugary Beverages:** "Drink clean, safe water. Avoid soda or sugar-sweetened beverages." (pg. 13)<br><br>**Quantity/Frequency:**<br>**Water:** "To maintain a steady state of fluid balance, a daily intake of about 2.5 L (10 cups) of fluid from drinks and food is recommended to match losses in urine, feces, sweat and breathing." (pg. 13)<br><br>**Sugary Beverages:** "Everyone should reduce intake of free sugars to less than 10% of daily calorie intake, and a further reduction to below 5% for additional benefits." (pg. 10)<br><br>**Image Content:**<br>N/A
Nauru (0)	N/A	
New Caledonia (0)	N/A	
Niue (0)	N/A	

Northern Mariana Islands (0)	N/A	
Philippines (11)	Nutritional Guidelines for Filipinos (2012)	**<u>What:</u>**<br>**Water:**<br><br>"Consume safe foods and water to prevent diarrhea and other food and water-borne diseases." (pg. 73)<br><br>"For good health, water must be consumed every day to replace the continuous loss of water in urine, perspiration, exhaled air, and feces." (pg. 22)<br><br>**Sugary Beverages:**<br><br>"Soft drinks are refreshing beverages that provide water and energy, but like coffee and tea should be consumed in moderation." (pg. 25)<br><br>"Choose and prepare foods and beverages with fewer added sugars to help control daily caloric intake." (pg. 97)<br><br>**<u>Where:</u>**<br>**Water:** "Consume safe foods and water to prevent diarrhea and other food and water-borne diseases." (pg. 73) Key Message/guideline.<br><br>**Sugary Beverages:** N/A<br><br>**<u>Why:</u>**<br>**Water:** "Water is the most essential of all nutrients in the sense that the absence of water causes death more quickly than the absence of any other nutrient." (pg. 22)<br><br>**Sugary Beverages:**<br><br>"Other sugars contribute calories which help provide energy and few other nutrients. Depending on the individual's choices, certain foods and beverages with added sugar might replace more nutrient-dense foods, along with the vitamins and minerals they provide." (pg. 95)<br><br>"A diet high in sucrose is associated with an elevation of plasma triglyceride concentrations." (pg. 96)<br><br>**<u>How:</u>**<br>**Water:** "Drink safe water." (pg. 83)

		**Sugary Beverages:**<br><br>"Drink water or other unsweetened beverages instead." (pg. 97)<br><br>"Choose and prepare foods and beverages with fewer added sugars to help control daily caloric intake." (pg. 97)<br><br>"Avoid sugary, non-diet sodas." (pg. 97)<br><br>**<u>Quantity/Frequency:</u>**<br>**Water:** "The water requirement for adults under average conditions of energy expenditure and environmental exposure is recommended to be 1 ml/kcal of energy expenditure. Thus, a person whose energy expenditure is 2000 kcal would require 2000 ml or 2 L of water (about 8 cups)." (pg. 22)<br><br>**Sugary Beverages:** "American women should consume no more than 100 calories a day from added sugar or from any source. It adds that most American men should consume no more than 150 calories a day from added sugar. This is equivalent to about 6 teaspoons of added sugar for women and 9 for men." (pg. 97)<br><br>**<u>Image Content:</u>**<br>Pyramid:<br>Visual representation of **water**<br>Visual representation of **sugary beverages**<br><br>**Plate:**<br>Visual representation of **water**
Samoa (0)	N/A	
Tonga (0)	N/A	
Tuvalu (9)	Tuvalu Guidelines for a Healthy Diet and Lifestyle (2021)	**<u>What:</u>**<br>**Water:** "Drink plenty of safe, clean water every day." (pg. 22)<br><br>**Sugary Beverages:** "Energy food to avoid: sugar-sweetened beverages." (pg. 7)<br><br>**<u>Where:</u>**<br>**Water:** "Drink plenty of safe, clean water every day." (pg. 22) Key message/guideline.<br><br>**Sugary Beverages:** N/A

		**Why:**<br>**Water:** "Water helps to keep the body cool, remove waste, lubricate joints, digest food, prevent constipation and carry oxygen and nutrients to body cells." (pg. 22)<br><br>**Sugary Beverages:** "A high intake of free sugars in the form of sugar-sweetened beverages is linked to obesity and dental caries." (pg. 17)<br><br>**How:**<br>**Water:**<br>"Drink plenty of safe, clean water every day." (pg. 22)<br><br>"Make safe drinking water available; boil it first if necessary" (pg. 23)<br><br>**Sugary Beverages:**<br>"Choose foods and drinks that are low in sugar." (pg. 18)<br><br>"Try reducing the amount of sugar added to coffee and tea from two teaspoons to one and eventually to none." (pg. 18)<br><br>"Avoid drinking too many high-sugar drinks." (pg. 23)<br><br>**Quantity/Frequency:**<br>**Water:** "To maintain a steady state of fluid balance, a daily intake of about 2.5 L (6-8 cups) of fluid from drinks and food is recommended to match losses in urine, faeces, sweat and breathing." (pg. 23)<br><br>**Sugary Beverages:** "Everyone should reduce intake of free sugars to less than 10% of daily calorie intake, and a further reduction to below 5% for additional benefits." (pg. 17)<br><br>**Image Content:** N/A
Vanuatu (0)	N/A	
Wallis and Futuna (0)	N/A	

*****These were selected examples from FBDGs and are not comprehensive of every relevant recommendation**
